# Carbon-based archiving: current progress and future prospects of DNA-based data storage

**DOI:** 10.1093/gigascience/giz075

**Published:** 2019-06-20

**Authors:** Zhi Ping, Dongzhao Ma, Xiaoluo Huang, Shihong Chen, Longying Liu, Fei Guo, Sha Joe Zhu, Yue Shen

**Affiliations:** 1 Guangdong Provincial Key Laboratory of Genome Read and Write, Shenzhen Engineering Laboratory for Innovative Molecular Diagnostics, Guangdong Provincial Academician Workstation of BGI Synthetic Genomics, BGI-Shenzhen, Shenzhen 518083, China; 2 Big Data Institute, University of Oxford, Li Ka Shing Centre for Health Information and Discovery, Old Road Campus, Oxford OX3 7LF, UK

**Keywords:** DNA digital storage, binary-DNA encoding scheme, *in vivo* DNA digital storage, *in vitro* DNA digital storage

## Abstract

The information explosion has led to a rapid increase in the amount of data requiring physical storage. However, in the near future, existing storage methods (i.e., magnetic and optical media) will be insufficient to store these exponentially growing data. Therefore, data scientists are continually looking for better, more stable, and space-efficient alternatives to store these huge datasets. Because of its unique biological properties, highly condensed DNA has great potential to become a storage material for the future. Indeed, DNA-based data storage has recently emerged as a promising approach for long-term digital information storage. This review summarizes state-of-the-art methods, including digital-to-DNA coding schemes and the media types used in DNA-based data storage, and provides an overview of recent progress achieved in this field and its exciting future.

## Introduction

The concept of DNA-based data storage was introduced by computer scientists and engineers in the 1960s [1]. In one pioneering attempt, made in 1988 by Joe Davis in his seminal artwork “Microvenus” [2], an icon was converted into a string of binary digits, encoded into a 28-bp synthetic DNA molecule, and was later successfully sequenced to retrieve the icon [2]. Although Microvenus was originally designed for interstellar communications, it demonstrated that non-biological information could also be stored in DNA. Later, in the early 2000s, Bancroft et al. proposed a simple way to use codon triplets for encoding alphabets, suggesting great potential for DNA as a storage medium [3]. Now we ask the question: what makes DNA so inimitable for data storage?

Four unique biological features make DNA the focus of the next generation of digital information storage. First, DNA is remarkably stable compared with other storage media. With its double-helix structure and base-stacking interactions, DNA can persist 1,000 times longer than a silicon device [4], and survive for millennia, even in harsh conditions [5–8]. Second, DNA possesses a high storage density. Theoretically, each gram of single-stranded DNA can store up to 455 exabytes of data [9]. As storage strategies continue to improve, scientists have now achieved a density that could reach this theoretical limit. Third, DNA can be easily and rapidly replicated through the PCR, thereby providing the possibility for large-scale data backup. It should not be neglected that living cells are also perfect tools for *in vivo* information replication and backup. Last but not least, the biological properties of DNA enable current sequencing and chemical synthesis technologies to read and write the information stored in DNA, thereby making it an excellent material to store and retrieve data [9].

The recently announced Lunar Library^TM^ project aims to create a DNA archive of a collection of 10,000 images and 20 books for long-term backup storage on the Moon. This highlights the advantage and immense potential of DNA as a medium for long-term digital data storage.

The accessibility of DNA-based data storage is mainly driven by 2 empowering techniques: DNA synthesis for “encoding,” and DNA sequencing for “decoding” [14]. Typically, digital information is first transcoded into ATCG sequences using a predeveloped coding scheme. These sequences are then synthesized into oligonucleotides (oligos) or long DNA fragments to allow long-term storage. To retrieve the data, a DNA sequencing method is applied to obtain the original ATCG sequence from the synthesized DNA.

## Overview of Current Coding Schemes for DNA-Based Data Storage

To summarize the findings of earlier studies, an optimal coding scheme usually outperforms in achieving 3 main features:

1. High fidelity—during data retrieval, there is a trade-off between accuracy and redundancy. While additional redundancy helps to improve accuracy, it also increases data size. Hence, to strike a balance, appropriate coding scheme and error correction strategies are applied to avoid and rectify errors induced during DNA synthesis or sequencing.
2. High coding efficiency—by having 4 elementary bases, DNA has the theoretical coding potential to store at least twice as much information in quaternary scaffolds as binary codes.
3. Flexible accessibility—from a computer science standpoint, stored data are expected to have random access. Lack of random access hampers attempts to scale up the data size because it will be impractical to sequence and decode the whole dataset each time when we only want to retrieve a small amount of data.

**Figure 1:**
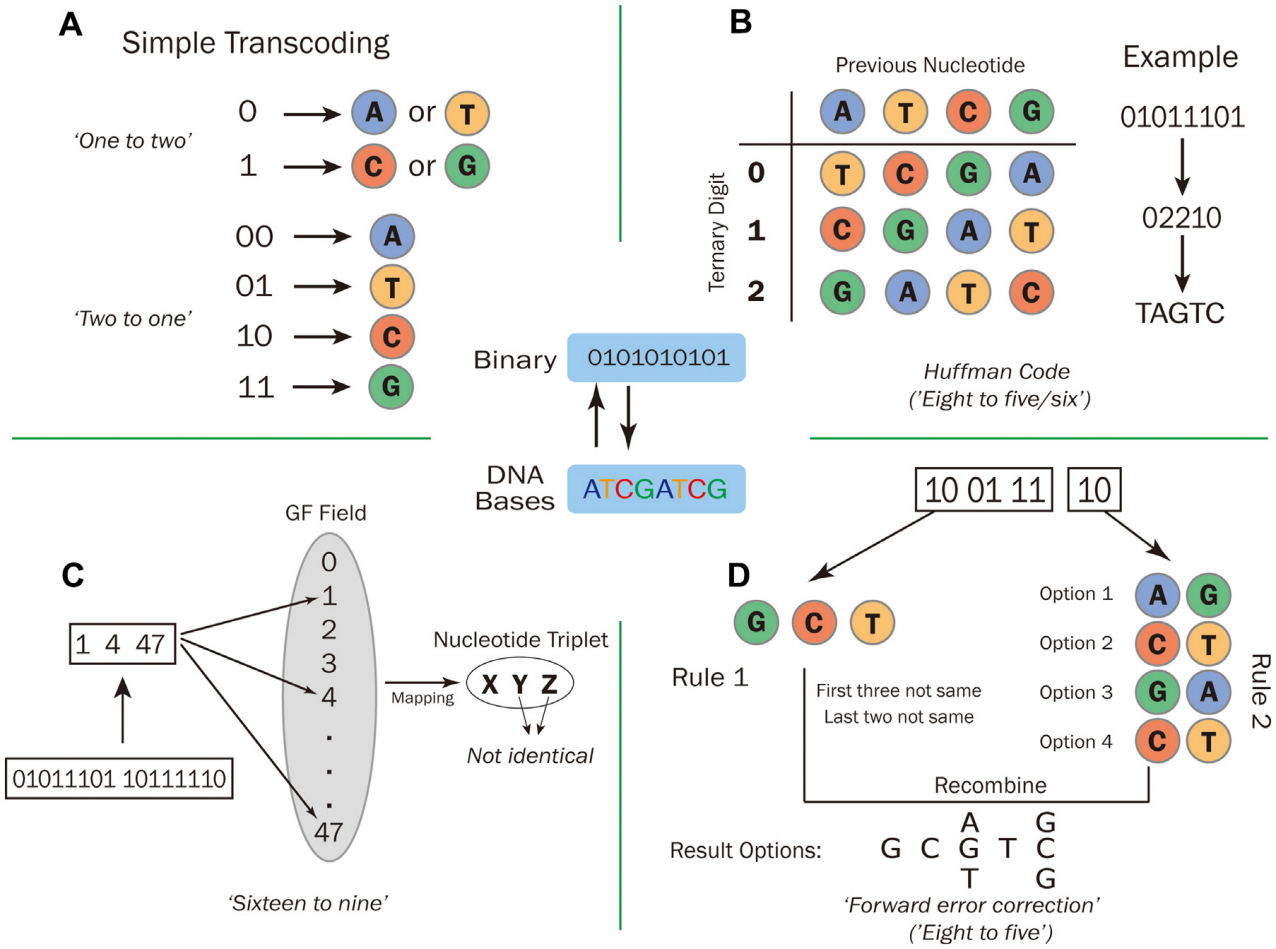
Binary transcoding methods used in DNA-based data storage schemes. (A) One binary bit is mapped to 2 optional bases [9]. Two binary bits are mapped to 1 fixed base [10]. (B) Eight binary bits are transcoded through Huffman coding and then transcoded to 5 or 6 bases [11]. (C) Two bytes (16 binary bits) are mapped to 9 bases [12]. (D) Eight binary bits are mapped to 5 bases [13].

**Figure 2:**
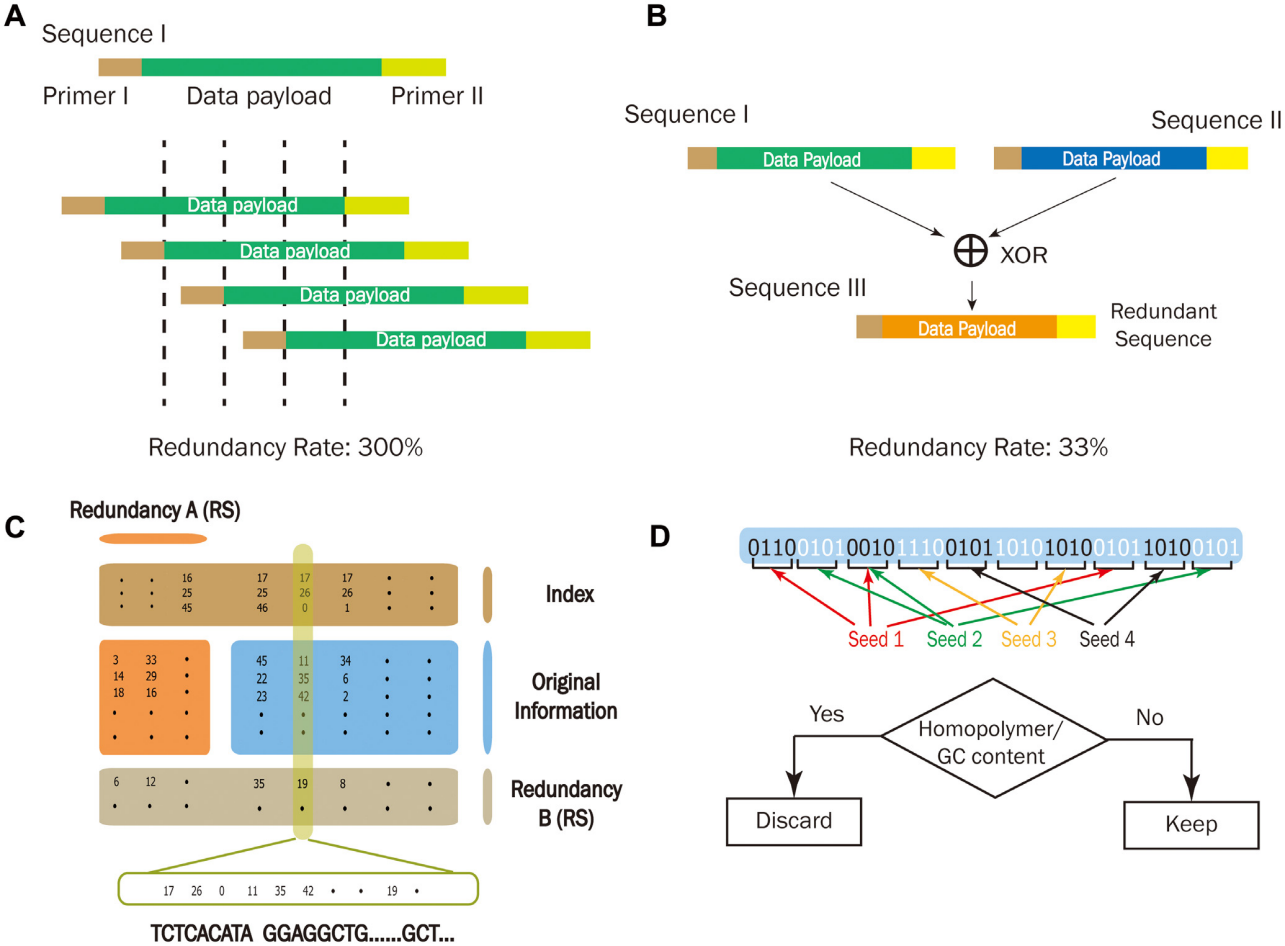
Redundancy types used in DNA-based data storage schemes. (A) Increasing redundancy by repetition. (B) Increasing redundancy by an exclusive-or (XOR) calculation. (C) Increasing redundancy using Reed-Solomon (RS) code for 2 rounds. (D) Increasing redundancy using fountain code.

Correspondingly, proposed coding schemes are usually designed to fulfill all of the above characteristics. Generally, DNA-based data storage coding schemes can be differentiated by their binary transcoding methods (Fig. 1), or by the ways in which they add redundancy to increase fidelity (Fig. 2).

**Figure 3:**
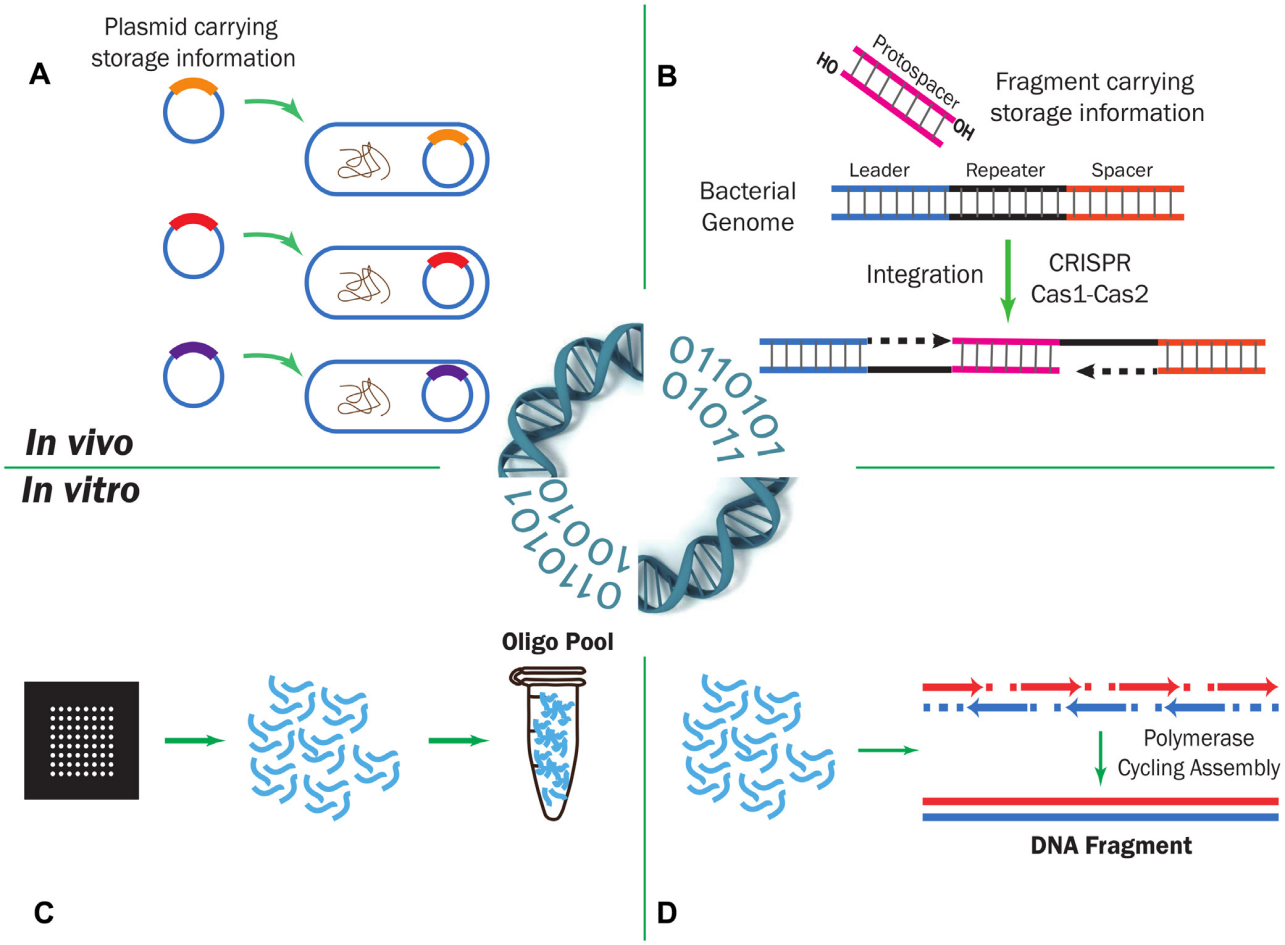
Two categories of DNA-based data storage application. (A) and (B) demonstrate 2 methods of *in vivo* DNA-based data storage; (C) and (D) demonstrate 2 methods of *in vitro* DNA-based data storage. (A) Array-based high-throughput DNA oligo analysis. DNA oligos carrying digital information are stored in the form of oligo pool. (B) DNA fragments synthesized by polymerase cycling assembly will carry the information to be stored. (C) Digital information inserted into a plasmid; plasmids are then transferred into bacterial cells. (D) DNA fragments carrying digital information are inserted into the bacterial genome using the CRISPR system using Cas1-Cas2 integrase.

### "Simple” code coding scheme

In 2012, Church et al. proposed a simple code to tackle errors generated by DNA sequencing and synthesis (e.g., repeated sequences, secondary structure, and abnormal GC content) [9]. By using the free base swap strategy (a “one-to-two” binary transcoding method; Fig. 1A), Church and colleagues encoded ∼0.65 MB data into ∼8.8 Mb DNA oligos of 159 nucleotides (nt) in length. Given the large amount of digital data that were successfully stored in DNA, this was considered to be a milestone study [15], and it also demonstrated the potential of DNA-based data storage to cope with the challenge of the information explosion. However, to allow its base swapping flexibility, this coding scheme sacrifices information density by transcoding each binary code into 1 base. Later researchers have developed other coding strategies to overcome this issue while maintaining comparable performance.

### Huffman coding scheme

Huffman code, developed by David Huffman in the 1950s, is considered to be an optimal prefixed code that is commonly used for lossless data compression. In 2013, Goldman and colleagues adopted the Huffman code in their coding scheme, which effectively improved the coding potential to 1.58 bits/nt [12]. Before transcoding into DNA nucleotides, binary data were first converted into ternary Huffman code, and then transcoded to DNA sequences by referring to a rotating encoding table (Fig. 1B). Each byte of the resulting data was substituted by 5 or 6 ternary digits (comprising the digits “0,” “1,” and “2” only) by Huffman's algorithm [16]. Encoding in this way, as per the rotating table, eliminates the generation of mononucleotide repeats and can compress the original data by 25–37.5%. For ASCII (American Standard Code for Information Interchange) text format files, this type of compression further outperforms by mapping the most common characters to 5-digit ternary strings [12]. However, the transcoding algorithm cannot prevent abnormal GC distribution when dealing with certain binary patterns. In addition, this coding scheme uses simple parity check coding to detect errors, and maintains a 4-fold coverage redundancy to prevent error and data loss (Fig. 2A). However, while the simple parity check coding can detect errors, it cannot correct them. Moreover, increased redundancy inevitably lowers the coding efficiency. Although not perfect, this work not only improved coding efficiency and prevented nucleotide homopolymers, but also introduced a strategy to ensure fidelity by adding redundancy.

### Improved Huffman coding scheme

In 2016, Bornholt et al. improved Goldman's encoding scheme with an exclusive-or (XOR) encoding principle [13], using an XOR (⊕) operation to yield redundancy. As shown in Fig. 2B, every 2 original sequences, A and B, will generate a redundant sequence C by A ⊕ B. Therefore, with any 2 sequences (AB, AC, or BC), one can easily recover the third sequence. This coding scheme also provides the flexibility of redundancy according to the level of significance of particular data strands, namely, “tunable redundancy.” It decreased the redundancy of the original data from 3-fold to half, providing an efficient way to ensure fidelity. In practice, this coding scheme successfully encodes 4 files with a total size of 151 KB and recovers 3 out of 4 files without manual intervention [13].

The need to amplify target files in a large-scale database suggests a necessity for random access in DNA-based data storage. Therefore, in 2018, Bornholt et al. put forward another error-free coding scheme that allowed users to randomly reach and recover individual files in a large-scale system. In this coding scheme, unique PCR primers are assigned to individual files after rigorous screening, thereby allowing users to randomly access their target file(s). A total of 200 MB data was successfully stored and recovered in their study, which set a new milestone by complementing the feasibility of storing large-scale data in DNA [14].

### A coding scheme based on Galois field and Reed-Solomon code

With special emphasis on error detection and correction, a coding scheme based on the Galois field (GF) and Reed-Solomon (RS) code [15] was proposed by Grass and colleagues in 2015 [17], improving potential data density to ∼1.78 bits/nt. With the 2-byte (8 × 2 bits) fundamental information block, this coding scheme introduced a finite field (the GF) of DNA nucleotide triplets as its elements (Fig. 1C). To prevent mononucleotide repeats of >3 nt during encoding, the last 2 nucleotides of the triplet are varied, which can give 48 different triplets. A GF of 47 was used because 47 is the largest prime number smaller than 48. The information block is then mapped to the 3 elements in GF (47), i.e., 256^2^ to 47^3^. The RS code is applied in this scheme to detect and correct errors. As shown in Fig. 2C, 2 rounds of RS coding are applied horizontally and vertically to the matrix generated by GF transcoding, respectively.

In this pilot study, 83 KB of text data were encoded *in silico* [17]. Although the data size was not impressive, it underlined the necessity to apply error correction coding, and significantly enhanced coding efficiency. Moreover, error correction code from the information communication field was applied to DNA-based data storage for the first time.

### A “forward error correction” coding scheme

Blawat and colleagues proposed a coding scheme to particularly tackle the errors generated during DNA sequencing, amplification, and synthesis (e.g., insertion, deletion, and substitution) [18]. The potential coding density was 1.6 bits/nt. Two reference coding tables are specified in advance. A 1-byte (8 bits) fundamental information block is assigned to a 5-nt DNA sequence, and the third and fourth nucleotide are swapped (Fig. 1D). Two other criteria are also applied to prevent mononucleotide repeats during this process: (i) the first 3 nucleotides should not be the same; and (ii) the last 2 nucleotides should not be the same. Consequently, an 8-bit data block (i.e., 2^8^ = 256 permutations for binary data) is transcoded into 704 different DNA blocks (4^5^ − 4^3^ − 4^4^) [18]. These can be categorized into 3 clusters: clusters A and B of complete blocks (256 each), and cluster C of 192 incomplete blocks. Data can then be mapped to DNA blocks A and B as required, e.g., alternately mapped to A or B.

In this study, 22 Mb of data were successfully encoded and stored in an oligo pool. Those data were retrieved without error, thereby proving the feasibility of the “forward error correction” coding scheme. However, this was not the case for detecting and correcting single mutations. For example, “11100011” could be mapped to a DNA block “TGTAG.” but if an A-to-T transversion occurs, the DNA block will be changed to “TGTTG,” which will give an error byte “11101111” after decoding.

### Fountain code−based DNA-based data storage coding scheme

In 2017, Erilich and Zielinski used fountain code in their coding scheme [19]. Fountain code is a widespread method of coding information in communication systems and is well known for its robustness and high efficiency [20]. Fountain code is also known as a rateless erasure code, in which data to be stored are divided into *k* segments, namely, resource packets. A potentially limitless number of encoded packets can be derived from these resource packets. When it returns *n* (*n* > *k*) encoded packets, the original resource data will be perfectly recovered. In practice, *n* only needs to be slightly larger than *k* to yield greater coding efficiency and robustness for information communication [21].

Binary data nucleotide sequence transcoding is also carried out. A fundamental 2-bit to 1-nt transcoding table is adopted, in which [00, 01, 10, 11] is mapped to [A, C, G, T], respectively (Fig. 1A). First, original binary information is segmented to small blocks. These blocks are chosen according to a pre-designed pseudorandom sequence of numbers. A new data block is then created by the bitwise addition of selected blocks with random seeds attached and transcoded to nucleotide blocks according to the transcoding table. Mononucleotide repeats and abnormal GC content are prevented by a final verification step (Fig. 2D) [19].

The oligos in this coding scheme are correlated and have grid-like topology to realize extremely low but necessary redundancy. This study increased the theoretical limit of coding potential to an unprecedentedly high value of 1.98 bits/nt, and remarkably reduced the desired redundancy for error-free recovery of the source file. Moreover, the mechanism of random selection and validity verification ensures that long single-nucleotide homopolymers do not appear in the encoded sequence. However, in this coding scheme, the complexity level of encoding and decoding is not linearly correlated to the data size. Thus, decoding can be complicated and may require more resources and a longer computation time. However, although it is claimed that a 4% loss of total packets would not affect the recovery of the original file in the report, in terms of the features of DNA fountain code, loss of more packets may cause complete failure of recovery. If the ultimate aim is to permanently store the data, the amount of redundancy must be increased to ensure information integrity.

If we consider DNA-based data storage solely as an archiving process with high fidelity, then DNA fountain coding appears to be the only communication-based coding scheme. In DNA-based data storage and retrieval, the most common error is caused by a single-nucleotide mutation. To address this issue, most coding schemes create high redundancy to tackle the challenging conditions of current communication channels. However, these error correction algorithms require complex decoding procedures and large amounts of computing resources. Here, the use of a fountain coding scheme first shows that it is unnecessary to use error detection/correction algorithms, and this provides us with an alternative solution for improving the performance of DNA coding.

## Overview of DNA-Based Data Storage Media

Currently, DNA-based data storage uses 2 main types of media to store encoded DNA sequences: *in vivo* and *in vitro*.

### In vivo

*In vivo* DNA-based data storage was commonly adopted in pioneering DNA-based data storage work, such as the Microvenus project, which used bacteria as the storage medium [2]. In the 2000s, other research teams also proposed simple techniques for *in vivo* DNA-based data storage, e.g., the use of codon triplets to encode alphabets [22] or bits [23] by either transferring plasmids or introducing site-directed mutagenesis. Typically, encoded DNA sequences are first cloned into a plasmid and then transferred into bacteria (Fig. 3A). Therefore, the DNA sequences, and the information they carry, can be maintained in tiny bacteria and their billions of descendants.

Nevertheless, the capacity of bacteria for carrying plasmids is limited by the type and size of plasmid. In addition, plasmid mutation is quite common in bacteria. During bacterial replication, take *Escherichia coli* as an example, the spontaneous mutation rate is 2.2 × 10^−10^ mutations per nucleotide per generation, or 1.0 × 10^−3^ mutations per genome per generation [24], with a generation time of 20–30 minutes, which—after a few years—might ultimately alter the information stored.

Recently, Shipman et al. demonstrated a novel method to encode an image and a short movie clip into the bacterial genome using the clustered regularly interspaced short palindromic repeats−CRISPR-associated protein (CRISPR-Cas) system with Cas1-Cas2 integrase (Fig. 3B) [25]. Although, reportedly, the CRISPR-Cas system is not equally efficient to all sequences, this work greatly improved the capability of *in vivo* DNA-based data storage.

### In vitro

*In vitro* DNA-based data storage is seen more frequently than the *in vivo* version in recent studies. The oligo library is one of the most popular forms (Fig. 3C), primarily because of the maturation of the array-based high-throughput oligo synthesis technique [26], which makes the synthesis of large numbers of DNA oligos more cost-effective.

During the synthesis process, each oligo is assigned a short tag, or index, because all oligos are mixed together for high-throughput synthesis and sequencing. The current oligo synthesis technique can generate, at most, 200-mers, with relatively high accuracy and purity [27]. Hence, the index should be as short as possible to save the information capacity in each oligo. Apparently, many more indices will be needed if more DNA oligo sequences are generated and mixed. However, similar to *in vivo* DNA-based data storage, the larger data size demands more DNA oligos for *in vitro* DNA-based data storage. This increases the size of indices in oligo and thus lowers the storage capacity and efficiency.

To overcome these problems, longer DNA fragments can be used instead of DNA oligos (Fig. 3D). In 2017, Yazdi et al. successfully encoded 3,633 bytes of information (2 images) into 17 DNA fragments, and recovered the image using homopolymer error correction [28]. Nevertheless, the current cost of DNA fragment synthesis is higher than that of oligo synthesis, which increases the overall cost of DNA fragment−based storage.

Above all, both *in vivo* and *in vitro* strategies have been used in current DNA-based data storage research. However, the nature of these 2 strategies demonstrates the use of different techniques and different application scenarios (Table 1). Although *in vivo* storage is a more complicated procedure than oligo pool synthesis in terms of backup cost, *in vivo* DNA-based data storage is more cost-effective. The cost of the *invitro* method has been reduced with the development of array-based oligo synthesis and high-throughput sequencing. Considering long-term storage, DNA in an *in vivo* condition will degrade more slowly than *in vitro*. Nevertheless, errors induced by mutations during replication *in vivo* are more significant than those induced by synthesis because of the high accuracy of current DNA synthesis technology.

**Table 1:** Comparison of *in vivo* and *in vitro* DNA-based data storage

Parameter	*In vivo*	*In vitro*
Medium	PlasmidBacterial genome	Oligo libraryLong DNA fragment
Information writing	Cloning and gene editing	Oligo synthesis
Main cause for error generation	MutationSequencing	Error in synthesis/sequencing
Advantage	Long-term storageCost-effective backup	High throughputLow error rateEasy for manipulation
Disadvantage	Limited DNA sizeMutation during replication	DNA degradationCost of index region

Other pioneering work goes beyond the aforementioned DNA-based data storage system. Song and Zeng proposed a strategy that they claim is able to detect and correct errors in each byte [29]. They transformed a short message into *E. coli* stellar competent cells and proved the reliability of their strategy; this was one of the first studies to evaluate the stability of *in vivo* storage. Lee et al. incorporated enzymatic DNA synthesis and DNA-based data storage principles, reporting an enzymatic DNA-based data storage strategy [30]. Nevertheless, the recent recombinase and CRISPR-Cas9 techniques cannot be neglected because they might also drive *in vivo* DNA-based data storage in diversiform. All of this research has laid a sound foundation for the global application of this novel storage medium.

## Challenges of DNA-Based Data Storage

**Figure 4:**
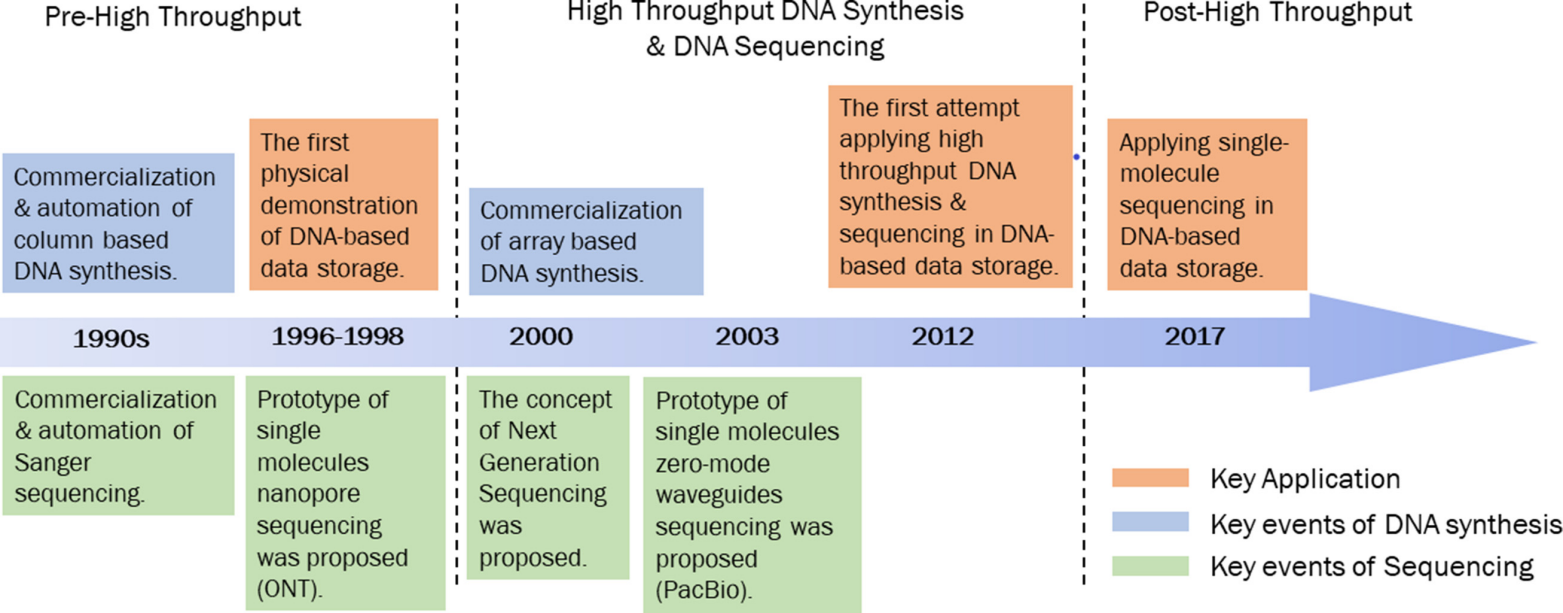
Key events in DNA synthesis and DNA sequencing, and their key applications in DNA-based data storage. PacBio: Pacific Biosciences.

Although DNA sequencing and DNA synthesis techniques largely facilitated the increase in DNA-based data storage, challenges co-derived and spontaneously evolve as each paradigm shift occurs in these fields. Fig. 4 shows a timeline briefly summarizing the key breakthroughs in DNA synthesis and sequencing that have transformed the development of DNA-based data storage.

In the pre–high-throughput period, column-based oligo synthesis [31] and Sanger sequencing [32, 33] represented the dominant DNA synthesis and DNA sequencing techniques, respectively. At this stage, the high cost ($0.05–0.15 USD per nucleotide in 100-nt synthesis; $1 USD per 600–700 bp per sequencing read) and time-consuming nature of DNA sequencing (an automated Sanger sequencing machine reads 1,000 bases per day) [10, 26] remain the major challenges for DNA-based data storage, preventing its application on larger datasets. Therefore, studies during that time were only conducted as a proof-of-concept on a relatively small scale [2].

From 2000 onwards, on the completion of the Human Genome Project, both DNA synthesis and DNA sequencing techniques were transformed to the high-throughput scale. Array-based oligo synthesis gradually superseded column-based oligo synthesis and was widely commercialized [34–36], largely because of its relatively low cost ($0.00001–0.001 USD per nucleotide synthesis [10]). However, as oligo length increases—presumably because of potential false cross-hybridization during synthesis—the error rate also increases. Moreover, the length of synthesized oligonucleotides is limited to <200-mers; this is because the product yield drops as oligos are elongated thanks to limitations in the efficiency of chemical interactions. Although gene size (200–3,000 bp or above) array-based synthesis has been developed [37], these usually require additional steps for error correction, causing the final cost and time consumed to be high. Consequently, for cost-saving purposes and to reduce the complexity of DNA synthesis, the primary storage unit used in DNA-based data storage is <200 nt.

The concept of massively parallel sequencing (or next-generation sequencing [NGS]), a high-throughput sequencing method, was proposed in 2000 [38]. In the following years, sequencing by ligation and by synthesis became major players in the sequencing field. Multiple NGS platforms became commercially available (e.g., 454, Solexa, Complete Genomics), which paved the way for high-throughput DNA-based data storage. However, this emerging technique also comes with limitations. Most NGS platforms require *in vitro* template amplification with primers to generate a complex template library for sequencing. During this process, copying errors, sequence-dependent biases (e.g., in high-GC and low-GC regions and at long mononucleotide repeats), and information loss (e.g., methylation) are produced [9].

In 2012, Church and colleagues successfully demonstrated the first application of high-throughput DNA synthesis and NGS in DNA-based data storage [9]. It initiated rapid development of coding schemes incorporating NGS. Two of the most common goals at this stage were how to improve coding efficiency and how to correct sequencing errors.

While NGS remains dominant, real-time, single-molecule sequencing (or third-generation sequencing) is continually evolving [39, 40]. Despite its relatively high sequencing error rate (∼10%), it is reportedly capable of long read-length sequencing, high-GC tolerant, and generates only random errors [28]. These characteristics mean it outperforms NGS counterparts and make it ideal for data retrieval in DNA-based data storage. In 2017, Yazdi et al. used Oxford Nanopore MinION technology to retrieve data stored in DNA, showing optimal robustness and high efficiency [28]. This study implies a possible shift from NGS to single-molecule sequencing because of its potential for compactness and stand-alone DNA data storage systems [13, 30]. Table 2 summarizes the frequently used sequencing platforms in DNA-based data storage. Recently, Oxford Nanopore Technologies announced plans to develop a “DNA writing” technique using their Nanopore technology. Using the same platform to both read and write, they claim it will be possible to selectively modify native bases and stimulate localized reactions, such as light pulses for encoding, which will provide real-time read and write capabilities for DNA-based data storage [41].

**Table 2:** Summary of frequently used sequencing platforms in DNA-based data storage (data retrieved from [42])

Platform	Error rate (%)	Runtime	Instrument cost (US$)	Cost per Gb (US$)	Reference
Illumina MiSeq	0.10	4–56 hours*	99,000	110–1,000*	[12, 15, 18, 25]
Illumina HiSeq 2000	0.26†	3–10 days*	654,000	41	[8, 11]
Illumina HiSeq 2500	0.10	7 hours–6 days^†,*^	690,000	30–230*	[17]
Illumina NextSeq	0.20†	11–29 h*	250,000	33–43*	[13]
Oxford Nanopore MinION	8.0†	≤48 h	1,000	70†	[13, 28]

†Latest data retrieved from the industrial report (may be different from previous literature); *varied by read length and reagent kit version.

In 2018, Oxford Nanopore also launched a high-throughput sequencing platform, PromethION, stating that it has the potential to yield up to 20 Tb of data in 48 hours [43, 44]. The first metagenomics data published using the PromethION demonstrated that it is already possible to obtain 150 Gb of data from 2 flowcells in a 64-hour run [45]. Further developments and improvements are in progress. Because the performance of this technology is getting closer to that of its NGS counterparts, it may play a more prominent role in the future study of DNA-based data storage.

## Perspectives on DNA-Based Data Storage

Taken together, DNA-based data storage techniques provide us with the great possibility to manipulate DNA as a carbon-based archive with excellent storage density and stability. Imperfect as it is, it may become the ultimate solution to the current data storage market for long-term archiving. We are also excited to see that multidisciplinary research companies have already joined this revolution to make DNA-based archiving commercially viable.

In terms of coding schemes, although the current theoretical limit of bit-base transcoding is 2 bits/base, newly discovered unnatural nucleic acids could expand the choice of bases for transcoding and thus increase the theoretical limit. X and Y are 2 classical unnatural nucleic acids that have demonstrated the capability to be integrated into normal cells, and in pairing, replication, and amplification [46]. Moreover, recent synthetic biology research reported 4 new synthetic nucleic acids: 6-Amino-5-nitropyridin-2-one (Z), 5-Aza-7-deazaguanine (P), Isocytosine (S), and Isoguanine (B) [47]. These new nucleic acid candidates could help to increase the coding efficiency for DNA digital storage in the not-too-far future.

Enterprises with a strong DNA synthesis background are most commonly seen, given that DNA-based data storage can significantly benefit from the breakthroughs achieved in DNA synthesis. It could be foreseen that with continuously improving enzymatic DNA synthesis techniques, DNA oligo synthesis could break the limit of 200-mers in the near future, providing us with a longer primary storage unit. This will undoubtedly improve net coding efficiency with the same lengths of PCR primers and shorter index sequences. In 1 model for the DNA-based storage of a 1-GB file under theoretical limitation, 1 DNA base represented 2 binary bits. For each DNA oligo, the length of forward and reverse primers was set at 20. In this case, we can deduce the equation representing the relationship between index length *i* and DNA oligo length *l*:

(1) \begin{eqnarray*}
\mathrm{log}_2( {l - 40 - i} ) + i = 32.
\end{eqnarray*}

Hence, we could obtain the correlation between an optimal index length and DNA oligo length.

**Figure 5:**
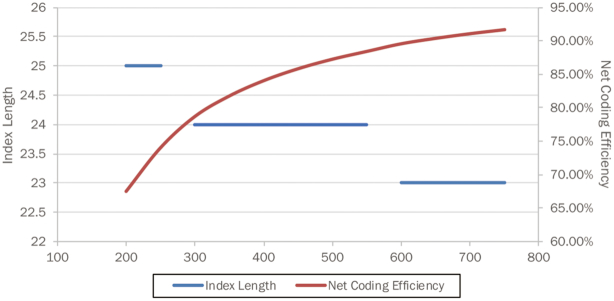
Interrelationship between DNA oligo length, optimal index length, and net coding efficiency in a model of 1-GB digital file transcoding.

As Fig. 5 shows, as DNA oligo length increases, the index length decreases, while net coding efficiency increases. Some start-up companies are now reportedly aiming to develop industrial enzymatic DNA synthesis technology. If they can successfully synthesize oligos >200-mers, the efficiency of DNA-based data storage will markedly improve.

In addition, the scale of DNA synthesis also affects the information capacity of DNA-based data storage per unit mass. With the development of array-based DNA synthesis technology, high-throughput oligo synthesis is currently directed to the microscale level. In DNA-based data storage, the information capacity of a certain mass of DNA sequences also relates to the copy number of each DNA molecule. The correlation between information capacity *C* and copy number *N_m_* of each oligo can be calculated from:

(2) \begin{eqnarray*}
C\ = \ n \times {( {{N_m}\mu \delta \gamma } )^{ - 1}},
\end{eqnarray*}

where *n* represents the number of bytes carried by each oligo (normally 10–20 bytes/molecule according to different coding schemes), *μ* is the number of nucleotides per molecule, *δ* is 320 Da/nt, and *γ* is 1.67 × 10^−24^ g/Da. To date, the copy number of oligos is ∼10^7^ molecules in on-chip high-throughput synthesis (without dilution) [19]. According to Equation 2, this will give an information capacity level of ∼10^13^ bytes/g. If the copy number is decreased to 10^4^ molecules per oligo, the information capacity will increase to ∼10^16^ bytes/g. Additionally, synthesis in microscale will also reduce the cost by several orders of magnitude and save the dilution step.

At present, several DNA synthesis companies are taking the lead in this field, based on their related expertise, and providing services related to DNA-based data storage. Twist Biosciences has reportedly already collaborated with Microsoft in a DNA-based data storage project, providing them with oligo pool services [14] using their high-throughput, array-based DNA synthesis technique. Microsoft, together with the University of Washington, launched the “Memories in DNA” project and will collaborate with the Arch Mission Foundation to construct the first Molecular Collection of the aforementioned Lunar Library. Given that these companies are starting to push this business forward, it will be interesting to see how commercial and social applications develop in the future.

Apart from companies with biology backgrounds, information technology (IT)-based industries are also playing an important role in this revolution. Because the coding schemes used in DNA-based data storage must yet be improved to yield higher coding efficiency and fidelity, efforts from the IT field could be of critical importance. For example, from random access data retrieval to scaling up data storage [13], Microsoft successfully implemented its IT philosophy in DNA-based data storage and is marching steadily towards its goal announced in 2017: a proto-commercial system in 3 years to storing some amount of data on DNA [48]. A recent paper written in collaboration with a scientist from the University of Washington described an automated end-to-end DNA-based data storage device, in which 5 bytes of data were automatically processed by the write, store, and read cycle [49]. Further efforts to speed up the coding and decoding process for daily storage applications are still essential.

We expect more entities and research organizations to join this cohort to eventually make carbon-based archiving a reality, and, furthermore, to attain immediate access storage or biological computation. Nevertheless, it remains a priority to maintain a safe and ethical framework for the development of DNA-based data storage. Because DNA is the basic building block of genetic information for living organisms, situations might arise in which synthesized sequences are introduced into living host organisms, and this could lead to biological incompatibility caused by unknown toxicity or other growth stresses. Hence, it is necessary to evaluate the safety of sequences prior to their synthesis. We long to see the day when the safety, capacity, and reliability of DNA means it will become the next-generation digital information storage medium of choice.

## Abbreviations

ATCG: adenine, thymine, cytosine, guanine; bp: base pairs; CRISPR: clustered regularly interspaced short palindromic repeats; Da: dalton; Gb: gigabase pairs; GF: Galois field; IT: information technology; KB: kilobytes; MB: megabytes; Mb: megabase pairs; NGS: next-generation sequencing; nt: nucleotide; oligos: oligonucleotides; RS: Reed-Solomon; XOL: exclusive-or.

## Competing interests

Z.P., D.Z.M., X.L.H., S.H.C., L.Y.L., F.G., and Y.S. are employees of BGI Shenzhen. The authors declare that they have no other competing interests.

## Funding

This work was supported by the Guangdong Provincial Academician Workstation of BGI Synthetic Genomics (No. 2017B090904014), Guangdong Provincial Key Laboratory of Genome Read and Write (No. 2017B030301011), and Shenzhen Engineering Laboratory for Innovative Molecular Diagnostics (DRC-SZ[2016]884).

## Authors’ contributions

Z.P., D.Z.M., and X.L.H. collected materials, reviewed the literature, and co-wrote the paper. S.H.C., L.Y.L., and F.G. supported materials collection and revised the paper. S.J.Z. and Y.S. supervised this review and co-wrote the paper. All authors read and approved the final manuscript.

## Supplementary Material

giz075_GIGA-D-18-00466_Original_Submission

giz075_GIGA-D-18-00466_Revision_1

giz075_GIGA-D-18-00466_Revision_2

giz075_Response_to_Reviewer_Comments_Original_Submission

giz075_Response_to_Reviewer_Comments_Revision_1

giz075_Reviewer_1_Report_Revision_1 Jeff Nivala -- 1/22/2019 Reviewed

giz075_Reviewer_2_Report_Revision_1 King L Chow, PhD -- 4/19/2019 Reviewed

giz075_Supplemental_Files
