# Supplementary material for: Carbon-based archiving: current progress and future prospects of DNA-based data storage
Source: Gigascience. 2019 Jun 20;8(6):giz075. doi: 10.1093/gigascience/giz075 (PMC6586197; doi:10.1093/gigascience/giz075)
Supplement: giz075_GIGA-D-18-00466_Original_Submission [file giz075_giga-d-18-00466_original_submission.pdf]

GigaScience

Carbon-based archiving: the current progress and future prospects of DNA-based data storage

--Manuscript Draft--

**Manuscript Number:**	GIGA-D-18-00466
**Full Title:**	Carbon-based archiving: the current progress and future prospects of DNA-based data storage
**Article Type:**	Review
**Funding Information:**	Guangdong Provincial Academician Workstation of BGI Synthetic Genomics (2017B090904014) — Dr. YUE SHEN
**Abstract:**	The information explosion has led to rapid increases in the amount of data to be physically stored. Yet one of the essential challenges is still looking for a better solution: How to store these large amounts of data in a space-efficient and stable way? DNA-based storage is a promising approach for long-term digital information storage as DNA holds great potentials because of its unique bio-properties. This review summarizes the state-in-art methods including digit-to-DNA coding schemes and the media types used in DNA storage to provide a general overview of the most recent progress achieved in this field.
**Corresponding Author:**	YUE SHEN<br><br>CHINA
**Corresponding Author Secondary Information:**	
**Corresponding Author's Institution:**	
**Corresponding Author's Secondary Institution:**	
**First Author:**	Zhi Ping
**First Author Secondary Information:**	
**Order of Authors:**	Zhi Ping
	Dongzhao Ma
	Xiaoluo Huang
	Shihong Chen
	Longying Liu
	YUE SHEN
	Sha Joe Zhu
**Order of Authors Secondary Information:**	
**Additional Information:**	
**Question**	**Response**
Are you submitting this manuscript to a special series or article collection?	No
**Experimental design and statistics**<br><br><br>Full details of the experimental design and statistical methods used should be given	Yes

Powered by Editorial Manager® and ProduXion Manager® from Aries Systems Corporation

in the Methods section, as detailed in our Minimum Standards Reporting Checklist. Information essential to interpreting the data presented should be made available in the figure legends.<br><br>Have you included all the information requested in your manuscript?	
**Resources**<br><br>A description of all resources used, including antibodies, cell lines, animals and software tools, with enough information to allow them to be uniquely identified, should be included in the Methods section. Authors are strongly encouraged to cite Research Resource Identifiers (RRIDs) for antibodies, model organisms and tools, where possible.<br><br>Have you included the information requested as detailed in our Minimum Standards Reporting Checklist?	Yes
**Availability of data and materials**<br><br>All datasets and code on which the conclusions of the paper rely must be either included in your submission or deposited in publicly available repositories (where available and ethically appropriate), referencing such data using a unique identifier in the references and in the "Availability of Data and Materials" section of your manuscript.<br><br>Have you have met the above requirement as detailed in our Minimum Standards Reporting Checklist?	Yes

Powered by Editorial Manager® and ProduXion Manager® from Aries Systems Corporation

Main text with figures and tables

Click here to download Manuscript Carbon-based Archiving - Draft-20181121-final version.docx

Carbon-based archiving: the current progress and future prospects of DNA-based data storage

Zhi Ping[1,†], Dongzhao Ma[1,†], Xiaoluo Huang[1,†], Shihong Chen[1], Longying Liu[1], Sha Joe Zhu[2,*], Yue Shen[1,*]

[*]Correspondence: shenyue@genomics.cn (Y.S.), joe.zhu@bdi.ox.ac.uk (S.J.Z.)

[†]These authors contributed equally to this work.

[1]BGI-Shenzhen, Shenzhen, 518083, China

[2]Big data Institute, University of Oxford, Li Ka Shing Centre for Health Information and Discovery, Old Road Campus, Oxford, OX3 7LF, United Kingdom

Abstract

The information explosion has led to rapid increases in the amount of data to be physically stored. Yet one of the essential challenges is still looking for a better solution: How to store these large amounts of data in a space-efficient and stable way? DNA-based storage is a promising approach for long-term digital information storage as DNA holds great potentials because of its unique bio-properties. This review summarizes the state-in-art methods including digit-to-DNA coding schemes and the media types used in DNA storage to provide a general overview of the most recent progress achieved in this field.

Keywords:

DNA digital storage, Binary-DNA encoding scheme, *in vivo*/*in vitro* DNA digital storage

Introduction to DNA-based storage

The concept of DNA-based storage was initially introduced by computer scientists and engineers in 1960s [1]. One of the pioneering attempts was made in 1988 by Joe Davis. At his seminal art work – "Microvenus" [2], Davis converted an icon into a string of binary digits, encoded them into a 28 base-pair (bp) synthetic DNA and later successfully sequenced to retrieve the "icon" [2]. Although Microvenus was originally designed for interstellar communications, it demonstrated that non-biological information could be also stored in DNA.

Three unique bio-features making DNA the focus of the next generation of digital-information storage. First, DNA is remarkably stable comparing to other storage media. With its double-helix-structure and base stacking interaction, DNA can last for a thousand times longer than a silicon device [3] and survive at harsh conditions over millennia [4,5,6,7]. Second, DNA possesses high storage density. Intuitively, each gram(g) of single-stranded DNA can maintain data up to 455 exabytes [8]. As the storage strategy is continuously optimized, scientists have already achieved a density that is very close to this theoretic limit. Last but not least, the biological property of DNA provides access to natural reading and writing enzyme which enables information stored in it remains accessible for millennia [8]. A recent announced project called "the Lunar Library™ project" aims to make a DNA archive with the collection of 10,000 images and 20 books for long-term backup storage on the Moon. This showcase suggests the potential and advantage of DNA as a medium in long-term digital storage.

The accessibility of DNA-based storage is mainly driven by two enabling techniques - DNA synthesis and DNA sequencing [9], of which the former serves for "encoding" and the later for "decoding". Typically, digital information is first transcoded into "ATCG" sequence using developed coding scheme. These sequences are then synthesized into oligo-nucleotides(oligos)

or long DNA fragments to allow long-term storage. To retrieve data, DNA sequencing is applied to obtain the original “ATCG” sequence from synthesized DNA and so the information stored in DNA.

Overview of current coding schemes for DNA storage

According to previous studies, we can summarize that an optimal coding scheme usually outperformances in achieving: 1. High fidelity. In data retrieval, there is an obvious trade-off between accuracy and redundancy. Hence, to strike a balance, appropriate coding scheme and error correcting strategy are applied to avoid and to correct errors induced by DNA synthesis or sequencing. 2. High coding efficiency. With four elementary bases, DNA has the theoretical coding potential to store information in quaternary scaffold at least twice as much as that of binary codes. 3. Flexible accessibility. From a computer science standpoint, data be stored is expected to have random access. Correspondingly, all proposed coding schemes are designed to fulfill the above features.

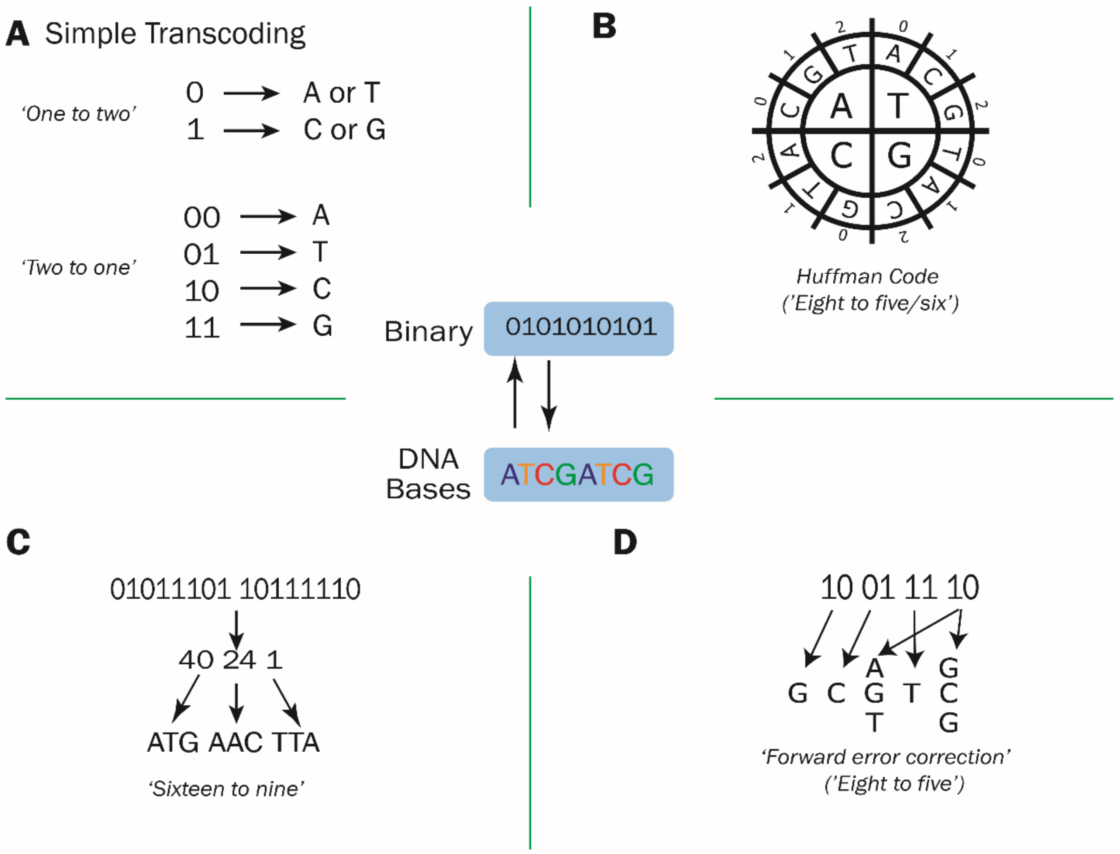

Figure 1 The different binary transcoding methods of reviewed DNA storage schemes. A) One binary bit is mapping to two optional bases [8]. B) Two binary bits are mapping to one fixed base [10]. C) Eight binary bits are transcoded through Huffman coding and then transcoded to five or six bases [11]. D) Two bytes (16 binary bits) are mapping to nine bases [12]. E) Eight binary bits are mapping to five bases [13].

- "Simple" code coding scheme

A "simple" code that aimed to tackle errors generated from DNA sequencing and synthesis (*e.g.* repeated sequences, secondary structure and abnormal GC content) was first proposed by Church et. al in 2012 [8]. By employing the free base swap strategy, Church and his colleagues encoded approximately 0.65 Mb data into ~8.8 Mb DNA oligos of 159 nt in length. It is considered as a milestone study in DNA storage given that large amount of digital data was successfully stored in DNA [14], which demonstrates the potential of DNA storage in coping with the challenge of information explosion. Yet, to allow its base swapping flexibility, this coding scheme sacrifices the information density - each binary code is transcoded into

one base (Fig. 1A). Researchers have later developed other coding strategies to overcome this issue while maintain comparable performance.

- Huffman coding scheme

In 2013, Goldman and colleagues adopted Huffman code in their coding scheme, which effectively improve the potential coding potential to 1.58 bits/nt [11]. Before transcoded into DNA nucleotides, binary data was first converted into ternary Huffman code and then transcoded to DNA sequence referred to a rotating encoding table (Fig. 1B). Every Byte of the resulting data would be substituted by five or six ternary digits (comprises “0”, “1”, “2” only), which can prevent generating mononucleotide repeats and compress the original data by 25% to 37.5%. Besides, for ASCII text format files, compression further outperformances by mapping the most common characters to five-digits ternary strings [11]. In addition, this coding scheme employed simple parity-check coding for error detection and maintained a four-fold coverage redundancy to prevent error and data loss (Fig. 2A). Nevertheless, it is noted that the simple parity-check coding can only detect but not correct the errors and the increased redundancy inevitably lower the coding efficiency.

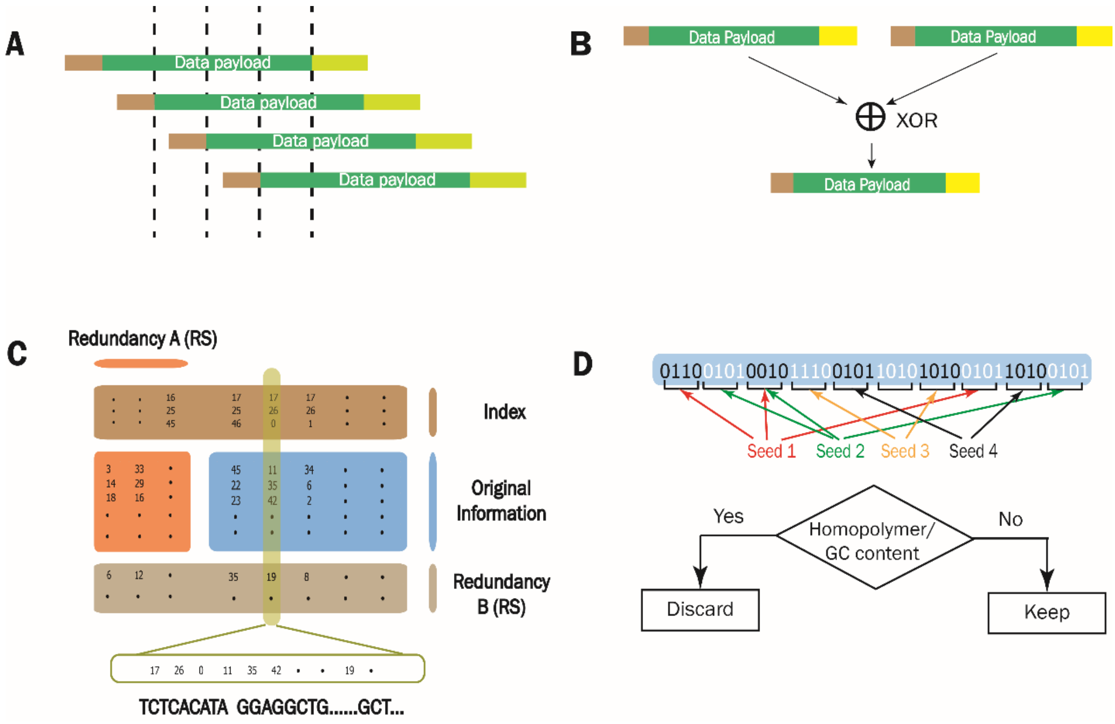

Figure 2. The different redundancy types used in the reviewed DNA storage schemes: A) Increasing redundancy by repetition; B) Increasing redundancy by an exclusive-or (XOR) calculation; C) Increasing redundancy by using Reed-Solomon code for two rounds; D) Increasing redundancy by using fountain code.

- Improved Huffman coding scheme

In 2016, Bornholt et. al improved Goldman's encoding scheme by an XOR encoding principle [12], which employed an exclusive-or (XOR, '⨁') operation to yield redundancy. As shown in Fig. 2B, every two original sequences, A and B, will generate a redundant sequence C by A⨁B. Therefore, with any two sequences (AB, AC or BC), one can easily recover the third sequence. Moreover, this coding scheme also provides the flexibility in providing redundancy according to the level of significance of particular data strands, namely "tunable redundancy". This coding scheme successfully encoded 4 files with the total size of 151 Kb and recovered 3 out 4 files without manual intervention [12].

Moreover, the need of amplifying target files in large-scale database suggested the necessity of random-access in DNA storage. Therefore, in 2018, the same team put forward another

error-free coding scheme that allowed the users to randomly reach and recover individual files in a large-scale system. In this coding scheme, unique polymerase chain reaction (PCR) primers are assigned to individual files after rigorous screening, therefore, it allows users to randomly access their target file(s). 200 Mb data was successfully stored and recovered in this study, which set a new milestone for providing the feasibility of DNA storage of large scale [13].

- A coding scheme based on Galois Field and Reed-Solomon Code

Focused on error detection and correction, a coding scheme based on Galois field and Reed-Solomon (RS) code [14] was proposed by Grass and colleagues in 2015 [15]. Meanwhile, the potential data density was improved to ~1.78bits/nt. With the two-byte (8×2 bits) fundamental information block, this coding scheme introduced a finite field (Galois field or GF) of DNA nucleotide triplets as its elements (Fig. 1C). To prevent mononucleotide repeat > 3nt during encoding, the last two nucleotides of the triplet are varied, which can give 48 different triplets. They indeed employed a GF(47) since 47 is the largest prime number smaller than 48 The information block is then mapped to the three elements in GF(47), *i.e.* 256^2 to 47^3. When conducted error detection and correction, RS code was applied in this scheme. As shown in Fig. 2C, two rounds of RS coding were applied horizontally and vertically to the matrix generated by GF transcoding respectively.

In this pilot study, 83 kilobytes of text data were encoded *in silico* [15]. Although the data size was not quite impressive it underlines the necessity of applying error-correction coding and significantly enhances the coding efficiency.

- A “forward error correction” coding scheme

Blawat and colleagues proposed a coding scheme focusing on tackling errors generated from DNA sequencing, amplification and synthesis (*e.g.* insertion, deletion and swapping). The potential coding density was 1.6bits/nt. Two reference coding tables are specified in advance. The one-byte (8 bits) fundamental information block is assigned to a 5 nt DNA sequence and

the 3rd and 4th nucleotide are swapped (Fig. 1D). Two other criteria are applied to prevent mononucleotide repeat during this process: 1) the first three nucleotides should not be the same; 2) the last two nucleotides should not be the same. Consequently, an 8-bits data block (i.e. 2^8 = 256 permutations for binary data) is transcoded into 704 different DNA blocks (4^5- 4^3- 4^4) [16]. They can be categorized into three clusters: clusters A & B of complete blocks, 256 per each and cluster C of 192 incomplete blocks. Data can then be mapped to DNA blocks A and B as required, e.g. alternately mapped to A or B.

In this study, 22Mbytes of data were successfully encoded and stored in an oligo pool. The data had been retrieved with no error, which proved the feasibility of this coding scheme. Yet, this is not the case for detecting and correcting single-mutation. For example, "11100011" could be mapped to a DNA block "TGTAG". However, if an A-to-T transversion occurs, the DNA block will change to "TGTTG", which will give an error byte "11101111" after decoding.

- Fountain code-based DNA storage coding scheme

In 2017, Erilich and Zielinski employed fountain code in their coding scheme [17]. Fountain code is a widespread coding method of information communication system known for its robustness and high efficiency [18]. Fountain code is also known as a rateless erasure code, in which data to be stored is divided into k segments, namely resource packets. Potentially limitless number of encoded packets can then derive from the resource packets. When it returns n ($n > k$) encoded packets, the original resource data should be perfectly recovered. In practice, n only need to be slightly larger than k to yield e great coding efficiency as well as robustness for information communication [19].

Similarly, binary data-nucleotide sequence encryption is carried out. A fundamental two-bit to one-nucleotide transcoding table is adopted, in which [00, 01, 10, 11] mapped to [A, C, G, T], respectively (Fig. 1A). At first, original binary information is segmented to small blocks. These blocks are chosen according to a pre-designed pseudorandom sequence of numbers. A

new data block is then created by bitwise addition of the selected blocks with random seeds attached and transcoded to nucleotide blocks according to the transcoding table. Mononucleotide repeats and abnormal GC content are prevented by a final verification (Fig. 2D) [17].

The oligos in this coding scheme are correlated and have grid-like topology to realize extremely low but necessary redundancy. This study enables the theoretical limit of coding potential unprecedentedly high, reaching1.98bits/nt and remarkably reduces the requested redundancy for an error-free recovery of source file. Moreover, the mechanism of random selection and validity verification ensures that long single-nucleotide homopolymers would not appear in the encoded sequence. However, in this coding scheme, the complexity level of encoding and decoding is not linearly correlated to the data size. Thus, decoding could be complicated and may require more resource and longer time for computation. However, although it is claimed that a 4% loss of total packets would not affect the recovery of original file in the report, in terms of the features of DNA Fountain code, loss of more packet may cause the complete failure of recovery. If it is aiming to store for permanent preservation, the amount of redundancy must be raised to ensure the information integrity when encounter spoiled oligo pools.

If DNA storage could be viewed as merely a storage process with high fidelity, DNA fountain coding is the present only communication-based coding scheme. In DNA data storage and retrieval, the most common error is caused by single nucleotide mutation. To address this issue, most coding scheme will create high redundancy in order to tackle the mal-condition of current communication channels, however, these error correction algorithms require complex decoding procedures and much computing time. Here, fountain-coding scheme firstly show that it is unnecessary to employ error detection/correction algorithms, which provide us an alternative solution towards improving the performance of DNA coding.

Overview of current media for DNA storage:

Current DNA storage employed different media to store the encoded DNA sequences. In sum, there are two types: *in vivo* and *in vitro*.

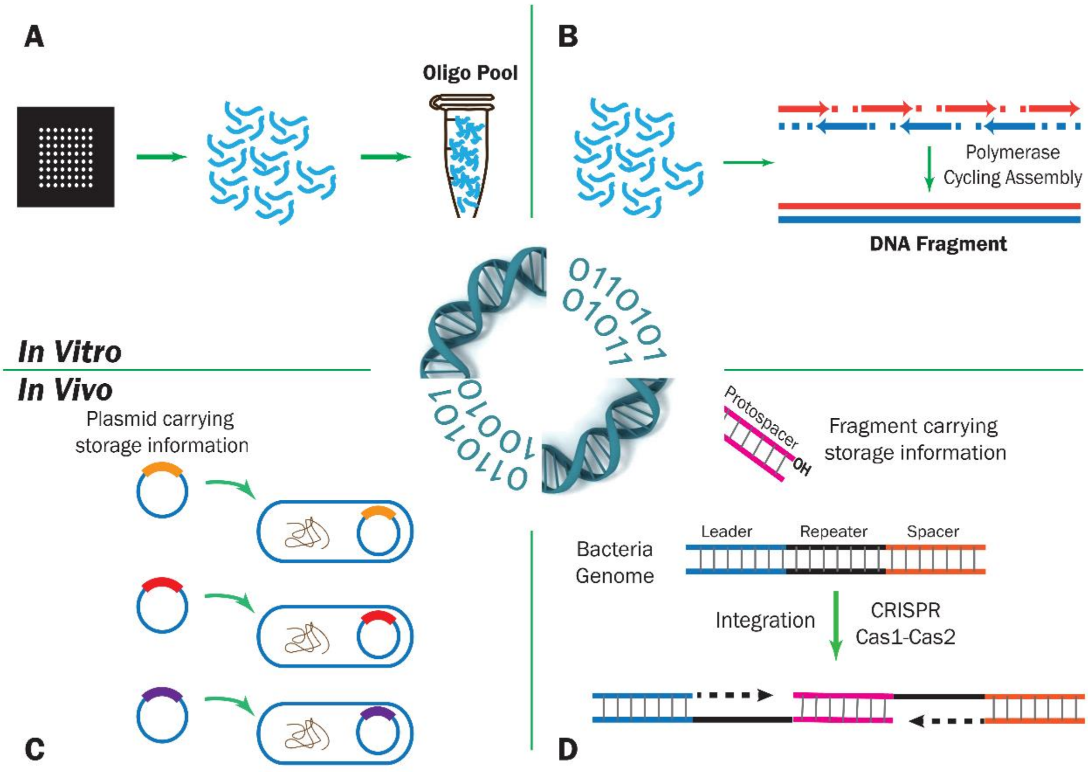

Figure 3. Two categories of DNA storage application. Panel A) and B) demonstrate the two ways of in vitro DNA storage; panel C) and D) demonstrate two ways of in vivo DNA storage. A) Chip-based high throughput DNA oligo analysis. DNA oligos carrying digital information are stored in the form of oligo pool. B) DNA fragments synthesized by polymerase cycling assembly (PCA), the fragments will carry the information to be stored. C) Digital information inserted into plasmid and then the plasmids are transferred into bacteria cells. D) DNA fragments carrying digital information is inserted into bacteria genome by employing CRISPR system using Cas1-Cas2 integrase.

In vivo DNA storage

In vivo DNA storage is commonly adopted in the pioneer works of DNA storage, such as the *Microvenus* project, which used bacteria as the storage medium. Typically, encoded DNA sequences are first cloned into plasmid and then transferred into the bacteria. Therefore, the

DNA sequences and so does the information it carries can be maintained in the tiny bacteria and their billions of descendants.

Nevertheless, the capacity of bacteria for carrying plasmid is limited by the type of plasmids and their corresponding size. In addition, the mutation of plasmid in bacteria is quite common. During bacteria replication, the spontaneous mutation may ultimately alter the information stored in them after a few years.

Recently, Church *et. al* demonstrated a novel method to encode an image and a short movie clip into the bacteria genome using the CRISPR-Cas system with Cas1-Cas2 integrase [20]. Although it is reported that CRISPR-Cas system is not equally efficient to all the sequences, this work greatly improved the capability of *in vivo* DNA storage.

***In vitro* DNA storage**

Apart from *in vivo* DNA storage, *in vitro* DNA storage is more frequently seen in recent studies. One of the most popular form is oligo library. This is largely due to the maturation of chip-based high-throughput oligo synthesis technique [21], making the synthesis of large amount of DNA oligos more cost-effective.

When synthesis, each oligo is given a short tag, or index, as all the oligos would be completely mixed for high throughput synthesis and sequencing. Current oligo synthesis technique is able to generate at most 200-mers in relatively high accuracy and purity [22]. Hence, the index should be as short as possible to save the information capacity in each oligo. Apparently, much more indices will be needed if more DNA oligo sequences are generated and mixed. However, similar to *in vivo* DNA storage, the larger the data size is, the more DNA oligos is demanded for *in vitro* DNA storage, which will increase the size of indices in oligo and thus lower the storing capacity and efficiency.

Alternatively, longer DNA fragments can be used instead of DNA oligos to avoid these problems. In 2017, Yadzi *et. al* successfully encoded 3633 bytes of information (two images)

into 17 DNA fragments and recovered the image using homopolymer error correction [23]. Nevertheless, the current cost of DNA fragment synthesis is higher than that of oligo synthesis, which increases the overall cost of DNA fragment-based storage.

Some other pioneer work also goes beyond our aforementioned DNA storage system. Song and Zeng proposed a strategy which is claimed to be able to detect and correct error in each byte [24]. They transformed short message into *E.coli* stellar competent cells and proved the reliability of their strategy. Lee *et. al* incorporated enzymatic DNA synthesis and DNA storage principles, reported an enzymatic-based DNA storage strategy [25]. All these researches laid a sound foundation for world-wide application of this novel storage medium.

Challenges of DNA-based storage

Limited size of synthetic DNA

As mentioned above, information encoding in DNA depends on DNA synthesis. Based on the final product size, DNA synthesis includes oligo synthesis (≤ 200 mer) and gene synthesis (200-3,000 bp or above), while DNA oligos usually serve as basic building blocks for gene synthesis. For cost saving purpose and to reduce complexity of DNA synthesis, primary storage unit size is often limited below 200nt [21].

Due to this limit, information needs to be fragmented and indexed before encoded into DNA to allow oligo synthesis (encoding) and pool sequencing (decoding) to reconstruct data in the correct order. Thus, when the amount of information grows, not only the number of fragments increase, but the indexing information also accumulates subsequently. Except for optimizing the index length (see “DNA storage in beyond” below), techniques for synthesizing longer oligo are considered to be the major challenge before we can push the envelope.

DNA sequencing-induced errors

Currently, there are two major types of DNA sequencing techniques: real-time, single-molecule sequencing and massively parallel (or next generation) sequencing. The latter is a high-throughput sequencing method and is dominant for short-read (<700bp, depending on platform) sequencing while the former is on the opposite [9,26].

In DNA storage, massively parallel sequencing is widely used for data retrieval ever since it's firstly employed by Church *et al*. in 2012. Two main reasons can explain this prevalence. First, the length of the synthetic DNA generated from encoding is relatively short, which is more cost-effective to sequence with massively parallel sequencing. Second, the throughput and accuracy (~99.9%) of massively parallel sequencing still far surpass its counterparts [9]. However, this technique also comes with limitation. Most massively parallel sequencing platforms require *in vitro* template amplification with primers to generate a complex template library for sequencing. During this process, copying errors, sequence-dependent biases (for example, in high- and low-GC regions and at long mononucleotide repeats) and information loss (for example, methylation) are produced [9].

Nevertheless, sequencing with minimal biases and random errors in respect to accuracy and contiguity is possible given that rapid progress is now achieved in real-time, single-molecule sequencing. It is reported that this rising technique can tolerant high GC content and only generates random errors [27], which is ideal in data retrieval. When it can also achieve high-fidelity, the storing potential of DNA may be further unlocked.

DNA storage in beyond

In spite of all progresses been achieved, current DNA-based storage is still at the early stage of its substantial applications. Moreover, its development will necessarily benefit from the progress of the coding/decoding methods.

It could be foreseen that in the near future, DNA oligo synthesis could break the limitation of 200-mers, providing us longer primary storage unit. This will clearly improve the net coding efficiency with same length of PCR primer and shorter index sequences. A simulation was performed for DNA storage of 1GB file under theoretical limitation, i.e. one DNA base would represent two binary bits. For each DNA oligo, the length of forward and reverse primers was set as 20. Therefore, we could get:

$$i + d + 20 \times 2 = l$$

Equation (1)

where *i* is the length of index, *d* is the length of data payload and *l* is the length of DNA oligo.

As the file size is 1GB, we could get:

$$d \times 2^i = \frac{1024^3 \times 8bits}{2}$$

Equation (2)

With combination of equation (1), therefore:

$$log_2(l - 40 - i) + i = 32$$

Equation (3)

Hence, we could get an optimal index length with fixed DNA oligo length.

As Figure 4 shows, with the increasing of DNA oligo length, the index length decreases while net coding efficiency increases. This calculation indicates that the efficiency of DNA storage could be remarkably improved with the improvement of DNA oligo synthesis techniques.

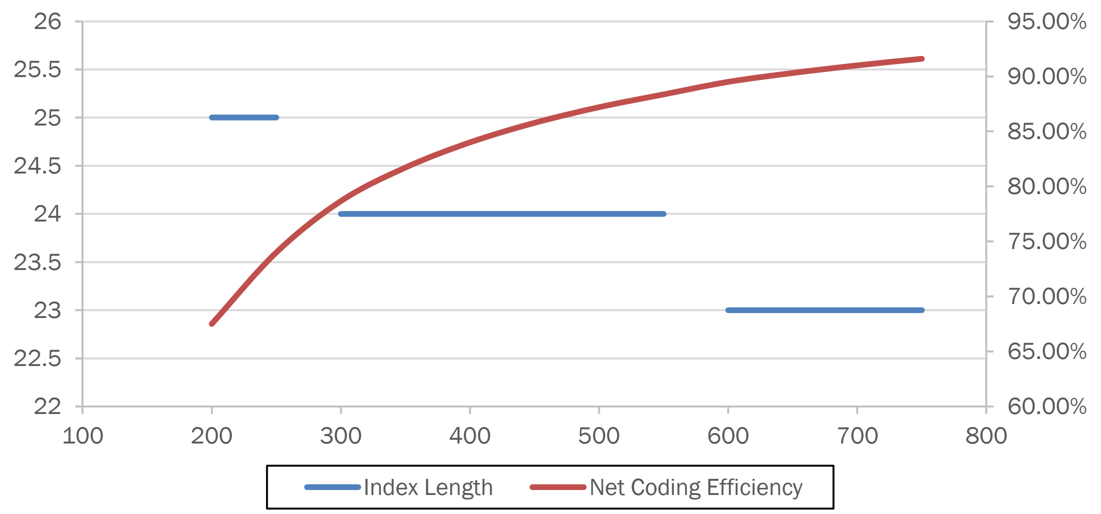

Figure 4 A simulation of net coding efficiency for DNA storage of 1GB file. The x-axis represents the length of oligo to be synthesized, y-axis (left) represents the minimum length of index needed to record the information coordinates, y-axis (right) represents the net coding efficiency.

In addition, the scale of DNA synthesis also affects the information capacity of DNA storage per unit mass. High-throughput oligo synthesis is currently directed to microscale level with the development of chip-based DNA synthesis technology. In DNA storage, the information capacity of certain mass of DNA sequences also relates to the copy number of each DNA molecule. To date, the copy number of oligos is around 10^7 molecules in microchip high throughput synthesis without dilution according to Erlich *et. al* [17], which will give an information capacity level at ~10^{13} bytes/g according to Equation (4). If the copy number decreased to 10^4 molecules per oligo, the information capacity will increase to ~10^{16} bytes/g. Additionally, synthesis in microscale also reduces the cost by several orders of magnitude and saves the step of dilution.

$$C = n \times (N_m \mu \delta \gamma)^{-1}$$

Equation (4)

where *C* represents the information capacity; *n* represents the number of bytes each oligo carries, normally 10 – 20 bytes/molecule according to different coding schemes; N_m is the

number of molecules, μ is the number of nucleotides per molecule, δ is 320 Dalton/nucleotide; γ is 1.67×10^{-24} g/Dalton,

On the other hand, development of sequencing technique also significantly effects DNA storage. By summarizing the frequent-used sequencing platforms in DNA storage, we noticed that accuracy and cost of sequencing are no longer the only considerations for DNA storage. Portable, yet error-prone sequencing platforms like Oxford Nanopore MinION is gaining attention due to its potential for high-compactness and stand-alone DNA data storage systems [13]. This trend will be largely attributed to the emergence of a growing number of error-tolerant coding schemes, which enable recovery of data even using the error-prone sequencing platform. If the accuracy of these sequencing platforms can be further improved, a paradigm shift from next-generation sequencing to third generation sequencing may eventually take place in DNA storage.

Platform	Error Rate	Runtime	Instrument Cost(US$)	Cost per Gb (US$)	Study
**Illumina MiSeq**	0.1%	21-56h*	$99K	$110-250	[12]Bornhol et al.,2016/[15]Grass et al.,2015/[17]Erlich Y and Zielinski D,2017/[20]Shipman et al.,2017
**Illumina HiSeq 2000**	2.0%	3-10d *	$654K	**$41**	[8]Church et al.,2012/[11]Goldman et al.,2013
**Illumina HiSeq 2500**	0.1%	7h-11d *	$690	**$30-250**	[16]Blawat et al.,2016
**Illumina NextSeq**	0.1%	<3d	$1K	**$7**	[13]Organick et al.,2018
**Oxford Nanopore MinION**	12.0%	up to 48h	$1K	**$750**	[13]Organick et al.,2018/[23]Yazdi et al.,2017

d, days; Gb, gigabase pairs; h, hours; K, thousand; * varied by read length and version of reagent kit

Table 1. Summary of frequent-used sequencing platforms in DNA storage (data retrieved from [26]).

Taken together, DNA storage provides us the possibility to manipulate DNA as a carbon-based archive with an excellent storage density and stability. By combining the within-reached development of DNA synthesis and sequencing techniques, DNA might eventually

transform into the next generation digital information storage media with ideal volatility, capacity, accessibility and reliability.

Acknowledgement

This work was supported by Guangdong Provincial Academician Workstation of BGI Synthetic Genomics (No. 2017B090904014).

References

1. Neiman MS. Some fundamental issues of microminiaturization. *Radiotekhnika*. 1964;No. 1:3-12.
2. Joe Davis a. Microvenus. Art Journal. 1996; 1:70. doi:10.2307/777811.
3. Bonnet J, Colotte M, Coudy D, Couallier V, Portier J, Morin B, *et a*l. Chain and conformation stability of solid-state DNA: implications for room temperature storage. *Nucleic Acids Research*. 2010;38 5:1531-46. doi:10.1093/nar/gkp1060.
4. Pääbo S, Poinar H, Serre D, Jaenicke-Després V, Hebler J, Rohland N, *et al*. GENETIC ANALYSES FROM ANCIENT DNA. *Annual Review of Genetics*. 2004;38:645-79. doi:10.1146/annurev.genet.37.110801.143214.
5. Kool ET. Hydrogen bonding, base stacking, and steric effects in DNA replication. Annual Review of Biophysics & Biomolecular Structure. 2001;30 1:1-22.

6. Nelson DL, Cox MM and Lehninger AL. *Lehninger principles of biochemistry*. New York ; Basingstoke : W.H. Freeman, c2008. 5th ed.; 2008

7. Pierce BA. Genetics : a conceptual approach. New York, NY : W.H. Freeman, c2012. 4th ed., International ed.; 2012.

8. Church GM, Gao Y and Kosuri S. Next-generation digital information storage in DNA. *Science*. 2012;337 6102:1628. doi:10.1126/science.1226355.

9. Shendure J, Balasubramanian S, Church GM, Gilbert W, Rogers J, Schloss JA, et al. DNA sequencing at 40: past, present and future. *Nature*. 2017;550 7676:345-53. doi:10.1038/nature24286.

10. De Silva PY and Ganegoda GU. New Trends of Digital Data Storage in DNA. *Biomed Res Int*. 2016;2016:8072463. doi:10.1155/2016/8072463.

11. Goldman N, Bertone P, Chen S, Dessimoz C, LeProust EM, Sipos B, et al. Towards practical, high-capacity, low-maintenance information storage in synthesized DNA. *Nature*. 2013;494 7435:77-80. doi:10.1038/nature11875.

12. Bornholt J, Lopez R, Carmean DM, Ceze L, Seelig G and Strauss K. A DNA-Based Archival Storage System. *SIGPLAN Not*. 2016;51 4:637-49. doi:10.1145/2954679.2872397.

13. Organick L, Ang SD, Chen YJ, Lopez R, Yekhanin S, Makarychev K, et al. Random access in large-scale DNA data storage. *Nat Biotechnol*. 2018;36 3:242-8. doi:10.1038/nbt.4079.

14. Reed I and Solomon G. Polynomial Codes Over Certain Finite Fields. *Journal of the Society for Industrial and Applied Mathematics*. 1960;8 2:300-4. doi:10.1137/0108018.

15. Grass RN, Heckel R, Puddu M, Paunescu D and Stark WJ. Robust chemical preservation of digital information on DNA in silica with error-correcting codes. *Angew Chem Int Ed Engl*. 2015;54 8:2552-5. doi:10.1002/anie.201411378.

16. Blawat M, Gaedke K, Hütter I, Chen X-M, Turczyk B, Inverso S, et al. Forward Error Correction for DNA Data Storage. *Procedia Computer Science*. 2016;80:1011-22. doi:https://doi.org/10.1016/j.procs.2016.05.398.

17. Erlich Y and Zielinski D. DNA Fountain enables a robust and efficient storage architecture. *Science*. 2017; 6328:950. doi:10.1126/science.aaj2038.

18. Byers JW, Luby M, Mitzenmacher M and Rege A. A digital fountain approach to reliable distribution of bulk data. *Proceedings of the ACM SIGCOMM '98 conference on Applications, technologies, architectures, and protocols for computer communication*. Vancouver, British Columbia, Canada: ACM, 1998, p. 56-67.

19. MacKay DJ. Fountain codes. *IEE Proceedings-Communications*. 2005;152 6:1062-8.

20. Shipman SL, Nivala J, Macklis JD and Church GM. CRISPR-Cas encoding of a digital movie into the genomes of a population of living bacteria. *Nature*. 2017;547 7663:345-9. doi:10.1038/nature23017.

21. Kosuri S and Church GM. Large-scale de novo DNA synthesis: technologies and applications. *Nature methods*. 2014;11 5:499-507. doi:10.1038/nmeth.2918.

22. Ma S, Tang N and Tian J. DNA synthesis, assembly and applications in synthetic biology. *Curr Opin Chem Biol*. 2012;16 3-4:260-7. doi:10.1016/j.cbpa.2012.05.001.

23. Yazdi S, Gabrys R and Milenkovic O. Portable and Error-Free DNA-Based Data Storage. *Sci Rep*. 2017;7 1:5011. doi:10.1038/s41598-017-05188-1.

24. Song L and Zeng AP. Orthogonal Information Encoding in Living Cells with High Error-Tolerance, Safety, and Fidelity. *ACS Synth Biol*. 2018;7 3:866-74. doi:10.1021/acssynbio.7b00382.

25. Lee HH, Kalhor R, Goela N, Bolot J and Church GM. Enzymatic DNA synthesis for digital information storage. *bioRxiv*. 2018; doi:10.1101/348987.

26. Goodwin S, McPherson JD and McCombie WR. Coming of age: ten years of next-generation sequencing technologies. *Nat Rev Genet*. 2016;17 6:333-51. doi:10.1038/nrg.2016.49.

27. Ross MG, Russ C, Costello M, Hollinger A, Lennon NJ, Hegarty R, *et al*. Characterizing and measuring bias in sequence data. *Genome Biol*. 2013;14 5:R51. doi:10.1186/gb-2013-14-5-r51.

28. van Dijk EL, Jaszczyszyn Y, Naquin D and Thermes C. The Third Revolution in Sequencing Technology. *Trends Genet*. 2018;34 9:666-81. doi:10.1016/j.tig.2018.05.008.

Table 1

Click here to download Table Table 1.tif

$141
$50-250
$7
$750

Figure 1 Click here to download Figure Figure 1.tif

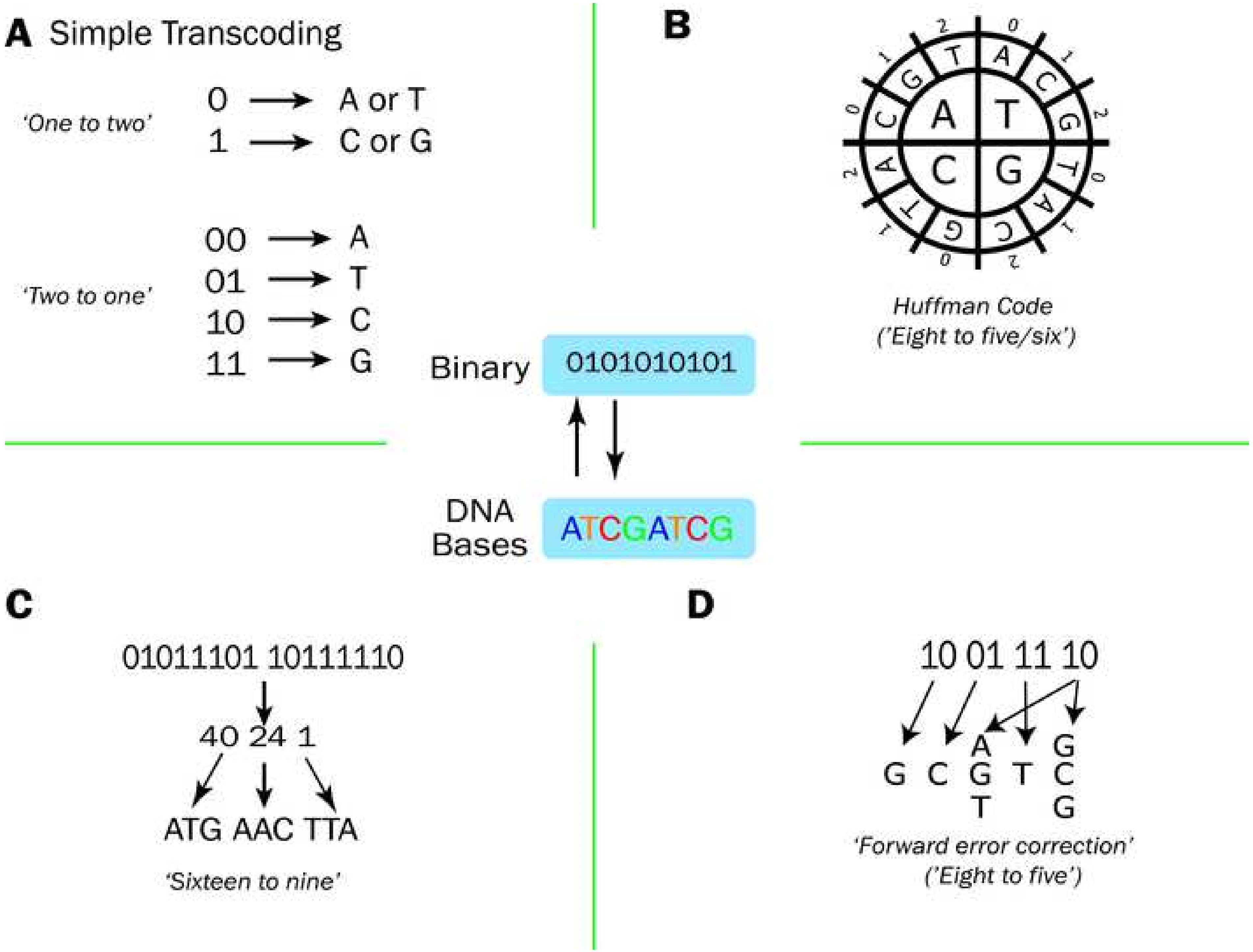

Figure 2

Click here to download Figure Figure 2.tif

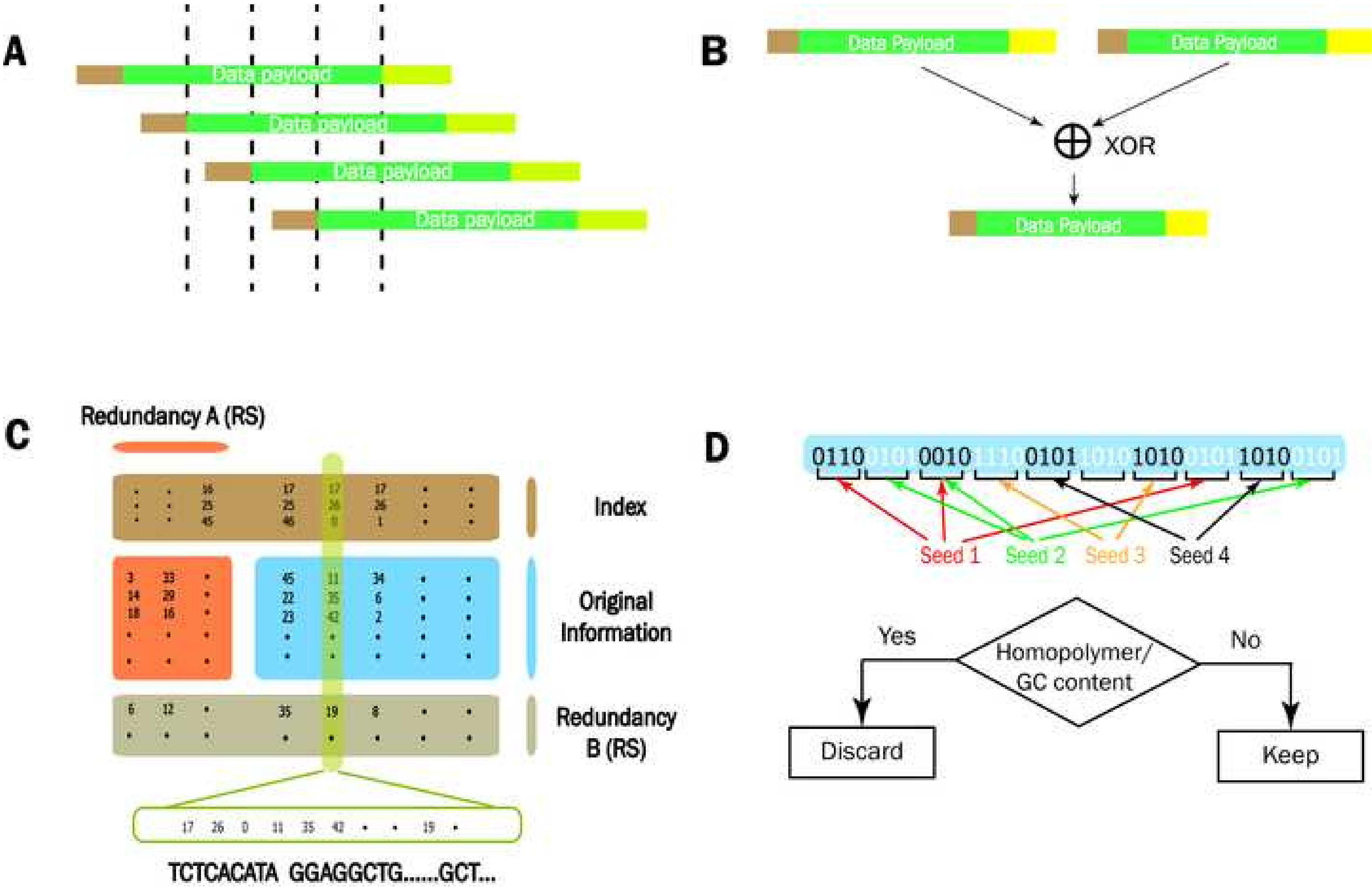

Figure 3

Click here to download Figure Figure 3.tif

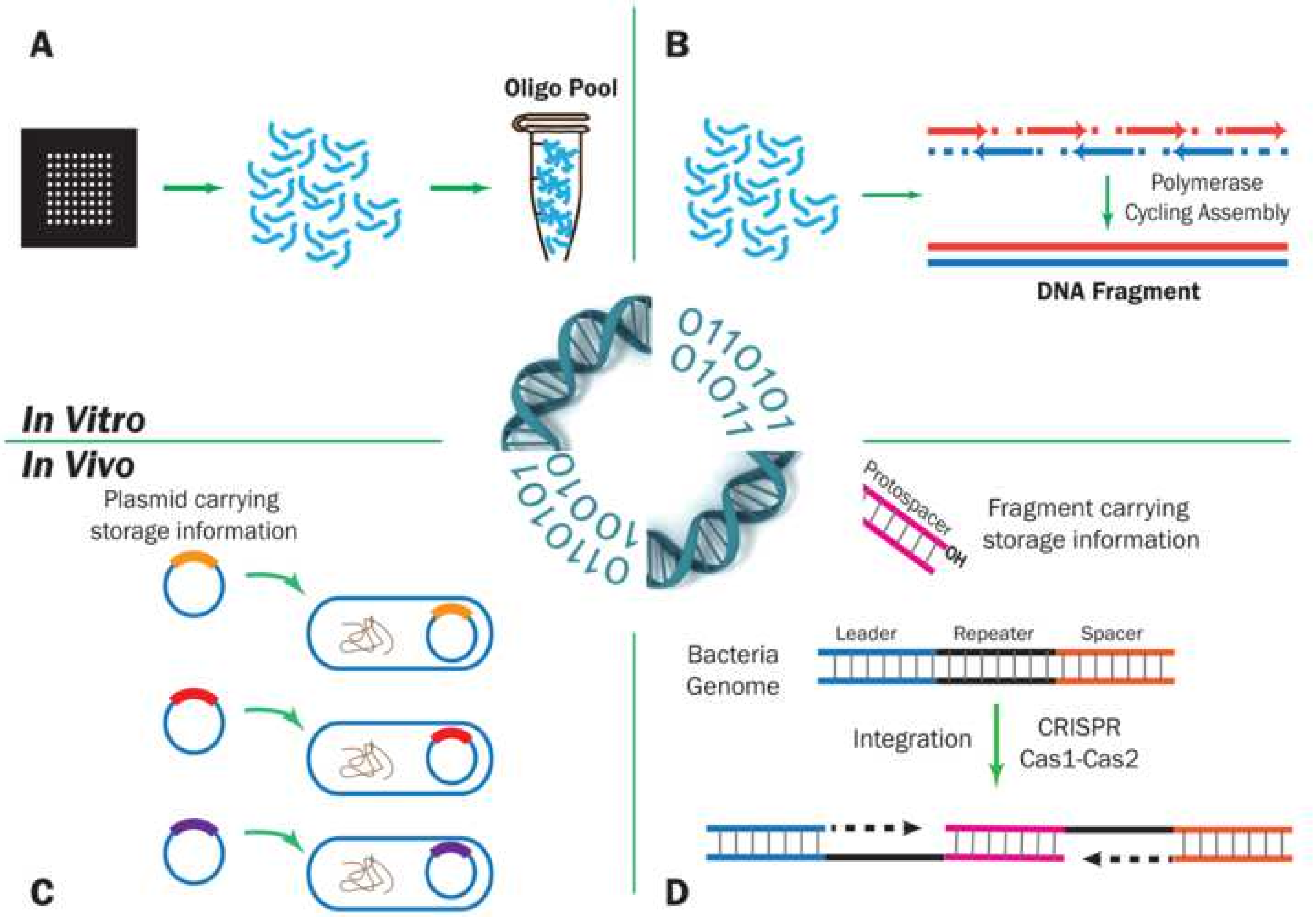

Figure 4

Click here to download Figure Figure 4.tif

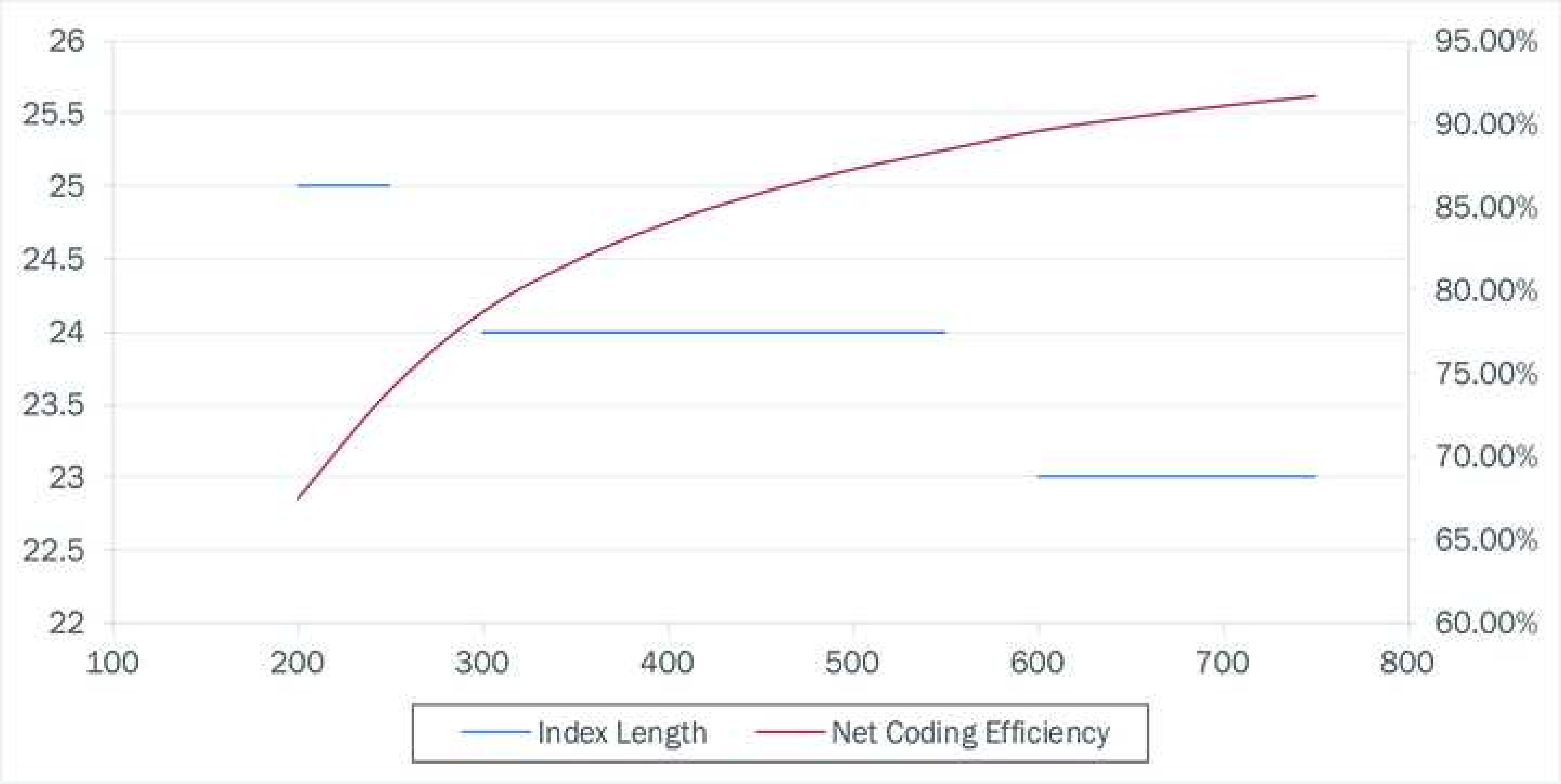

Click here to download Personal Cover Cover letter (Carbon-based Archiving)-old.docx

Dear Dr. Edmunds:

We submit our manuscript entitled “Carbon-based archiving: the current progress and future prospects of DNA-based data storage" to GigaScience for publication.

This manuscript is a timely review of DNA-based storage with focus on coding scheme and media type. We provide scalable measurements and technical opinions of this field, which we believe will be a great add on to people’s current understanding and help promote its better development. As DNA-based storage is a promising bio-approach for large scale digital information storage, we consider it is well in scope of the GigaScience’s publication criteria.

All authors have read and have abided by the publication ethics as set out by the Commission on Publication Ethics (COPE) for manuscripts submitted to GigaScience.

All authors declared that they have no conflicts of interest to this work.

The work described has not been submitted elsewhere for publication, in whole or in part, and all the authors listed have approved the manuscript that is enclosed.

Thank you very much for your attention and consideration.

Yours sincerely,

Yue (Chantal) Shen

Sha Joe Zhu
