# Supplementary material for: Carbon-based archiving: current progress and future prospects of DNA-based data storage
Source: Gigascience. 2019 Jun 20;8(6):giz075. doi: 10.1093/gigascience/giz075 (PMC6586197; doi:10.1093/gigascience/giz075)
Supplement: giz075_GIGA-D-18-00466_Revision_1 [file giz075_giga-d-18-00466_revision_1.pdf]

GigaScience

Carbon-based archiving: the current progress and future prospects of DNA-based data storage

--Manuscript Draft--

**Manuscript Number:**	GIGA-D-18-00466R1
**Full Title:**	Carbon-based archiving: the current progress and future prospects of DNA-based data storage
**Article Type:**	Review
**Funding Information:**	Guangdong Provincial Academician Workstation of BGI Synthetic Genomics (2017B090904014) — Dr. YUE SHEN
**Abstract:**	The information explosion has led to a rapid increase in the amount of data to be physically stored. But, the existing storage method (magnetic and optical media) will not be sufficient to store this exponentially growing data in near future. Therefore, the data scientists are continuously looking for better alternatives to store these hefty amounts of data in a space-efficient and stable way. Due to its unique biological properties, the highly dense "DNA" holds a great potential to become the future storage material. In fact, DNA-based data storage has recently emerged as a promising approach for long-term digital information storage. This review summarizes the state-of-the-art methods including digital-to-DNA coding schemes and the media types used in DNA-based data storage, and provide a general overview of the most recent progress achieved in this field and its exciting future.
**Corresponding Author:**	YUE SHEN<br><br>CHINA
**Corresponding Author Secondary Information:**	
**Corresponding Author's Institution:**	
**Corresponding Author's Secondary Institution:**	
**First Author:**	Zhi Ping
**First Author Secondary Information:**	
**Order of Authors:**	Zhi Ping
	Dongzhao Ma
	Xiaoluo Huang
	Shihong Chen
	Longying Liu
	YUE SHEN
	Sha Joe Zhu
**Order of Authors Secondary Information:**	
**Response to Reviewers:**	Dear Dr. Edmund,<br><br>Thank you for the precious advises and we did corrections and revisions on the whole manuscript accordingly.<br><br>For the information from twitter you provided in your comments, we tried our best to find some official announcements of this 22 Gb per flow cell, but we could not found it. So we decided not to provide this information in our manuscript.<br><br>Thank you again for you kind suggestions.

Powered by Editorial Manager® and ProduXion Manager® from Aries Systems Corporation

	Best,<br><br>Zhi PING, Ph.D<br>Institute of Genome Synthesis and Editing, China National GeneBank<br>BGI-Research<br>Address: China National GeneBank (CNGB), Jinsha Road, Dapeng District, Shenzhen, Guangdong, China
**Additional Information:**	
**Question**	**Response**
Are you submitting this manuscript to a special series or article collection?	No
**Experimental design and statistics**<br><br>Full details of the experimental design and statistical methods used should be given in the Methods section, as detailed in our Minimum Standards Reporting Checklist. Information essential to interpreting the data presented should be made available in the figure legends.<br><br>Have you included all the information requested in your manuscript?	Yes
**Resources**<br><br>A description of all resources used, including antibodies, cell lines, animals and software tools, with enough information to allow them to be uniquely identified, should be included in the Methods section. Authors are strongly encouraged to cite Research Resource Identifiers (RRIDs) for antibodies, model organisms and tools, where possible.<br><br>Have you included the information requested as detailed in our Minimum Standards Reporting Checklist?	Yes
**Availability of data and materials**<br><br>All datasets and code on which the conclusions of the paper rely must be either included in your submission or	Yes

deposited in publicly available repositories (where available and ethically appropriate), referencing such data using a unique identifier in the references and in the “Availability of Data and Materials” section of your manuscript.<br><br>Have you have met the above requirement as detailed in our Minimum Standards Reporting Checklist?	

Manuscript
Click here to view linked References
Click here to access/download;Manuscript;Carbon-based Archiving -V2.docx

Carbon-based archiving: the current progress and future prospects of DNA-based data storage

Zhi Ping[1,†], Dongzhao Ma[1,†], Xiaoluo Huang[1,†], Shihong Chen[1], Longying Liu[1], Sha Joe Zhu[2,*], Yue Shen[1,*]

[*]Correspondence: shenyue@genomics.cn (Y.S.), joe.zhu@bdi.ox.ac.uk (S.J.Z.)

[†]These authors contributed equally to this work.

[1]BGI-Shenzhen, Shenzhen, 518083, China

[2]Big data Institute, University of Oxford, Li Ka Shing Centre for Health Information and Discovery, Old Road Campus, Oxford, OX3 7LF, United Kingdom

Abstract

The information explosion has led to a rapid increase in the amount of data to be physically stored. But, the existing storage method (magnetic and optical media) will not be sufficient to store this exponentially growing data in near future. Therefore, the data scientists are continuously looking for better alternatives to store these hefty amounts of data in a space-efficient and stable way. Due to its unique biological properties, the highly dense “DNA” holds a great potential to become the future storage material. In fact, DNA-based data storage has recently emerged as a promising approach for long-term digital information storage. This review summarizes the state-of-the-art methods including digital-to-DNA coding schemes and the media types used in DNA-based data storage, and provide a general overview of the most recent progress achieved in this field and its exciting future.

Keywords:

DNA digital storage, Binary-DNA encoding scheme, *in vivo*/*in vitro* DNA digital storage

Abbreviations

ASCII: American Standard Code for Information Interchange; bp: base pair; DNA oligos: DNA oligonucleotides; GB: Giga-bytes; Gb: Giga-base-pairs; GF: Galois field; IAS: Immediate Access Storage; KB: Kilo-bytes; MB: Mega-bytes; Mb: Mega-bases; nt: nucleotide; RS: Reed-Solomon.

Introduction to DNA-based data storage

The concept of DNA-based data storage was initially introduced by computer scientists and engineers in 1960s [1]. One of the pioneering attempts was made in 1988 by Joe Davis in his seminal artwork – "Microvenus" [2], Davis converted an icon into a string of binary digits, encoded them into a 28 base-pair (bp) synthetic DNA and later successfully sequenced it to retrieve the "icon" [2]. Although Microvenus was originally designed for interstellar communications, it demonstrated that non-biological information could also be stored in DNA. Now the question comes, what makes DNA so inimitable for data storage?

There are three unique biological-features that make DNA the focus of the next generation of digital information storage. Firstly, DNA is remarkably stable compared with other storage media. With its double-helix-structure and base stacking interaction, DNA can last for a thousand times longer than a silicon device [3] and thrive in harsh conditions over millennia [4,5,6,7]. Secondly, DNA possesses a high storage density. Theoretically, each gram (g) of single-stranded DNA can store up to 455 exabytes of data [8]. As the storage strategy is continuously being optimized, scientists have now achieved a density that is very close to this theoretical limit (will be reviewed in the following section). Last but not the least, the biological properties of DNA enable the current sequencing and chemical synthesis technologies to read and write the information stored in DNA, thereby making it an excellent material to store and retrieve the data [8]. The recently announced "the Lunar Library™ project" aims to make a DNA archive with the collection of 10,000 images and 20 books for long-term backup storage on the Moon. This highlights the advantage and immense potential of DNA as a medium for long-term digital data storage.

The accessibility of DNA-based data storage is mainly driven by two empowering techniques - DNA synthesis and DNA sequencing [9], of which the former serves for "encoding" and the later for "decoding". Typically, digital information is first transcoded into "ATCG"

sequence using a predeveloped coding scheme. These sequences are then synthesized into oligonucleotides (oligos) or long DNA fragments to allow long-term storage. To retrieve the data, DNA sequencing method is applied to obtain the original “ATCG” sequence from the synthesized DNA.

Overview of current coding schemes for DNA-based data storage

Based on the earlier studies, it can be summarized that an optimal coding scheme usually outperforms in achieving three main features: 1. High fidelity. During data retrieval, there is an obvious trade-off between accuracy and redundancy. Hence, to strike a balance, appropriate coding scheme and error correcting strategy are applied to avoid and rectify errors induced during DNA synthesis or sequencing. 2. High coding efficiency. By having four elementary bases, DNA has the theoretical coding potential to store information in quaternary scaffold at least twice as much as that of binary codes. 3. Flexible accessibility. From a computer science standpoint, the stored data is expected to have random access. Correspondingly, all the proposed coding schemes are usually designed to fulfill all the above features.

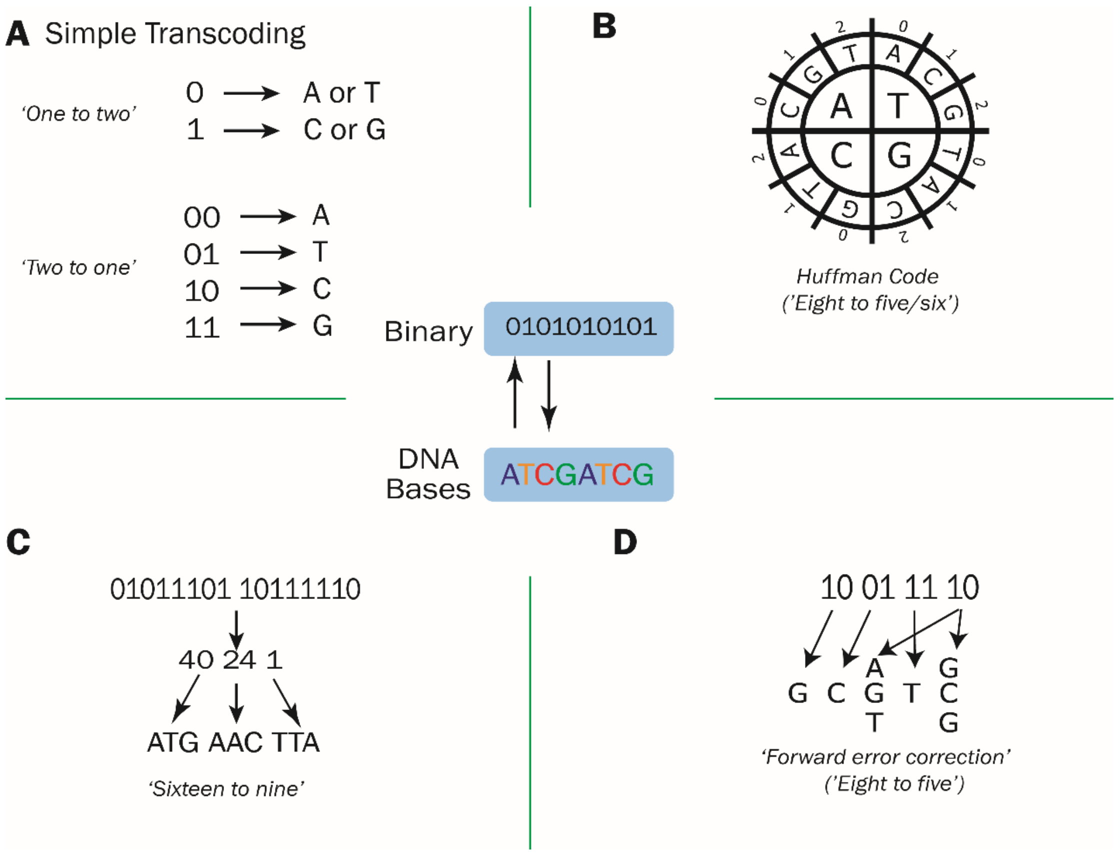

Figure 1 The different binary transcoding methods used in DNA-based data storage schemes. A) One binary bit is mapped to two optional bases [8]. B) Two binary bits are mapped to one fixed base [10]. C) Eight binary bits are transcoded through Huffman coding and then transcoded to five or six bases [11]. D) Two bytes (16 binary bits) are mapped to nine bases [12]. E) Eight binary bits are mapped to five bases [13].

- "Simple" code coding scheme

A "simple" code that aimed to tackle errors generated from DNA sequencing and synthesis (*e.g.* repeated sequences, secondary structure and abnormal GC content) was first proposed by Church et. al in 2012 [8]. By employing the free base swap strategy, Church and his colleagues encoded approximately 0.65 MB data into ~8.8 Mb DNA oligos of 159 nt in length. It is considered as a milestone study in DNA-based data storage given that a large amount of digital data was successfully stored in DNA [14], which also demonstrated the potential of DNA-based data storage in coping with the challenge of information explosion. However, to allow its base swapping flexibility, this coding scheme sacrifices the information density where each binary code is transcoded into a base (Fig. 1A). Researchers have later

developed other coding strategies to overcome this issue while maintaining the comparable performance.

- Huffman coding scheme

In 2013, Goldman and colleagues adopted Huffman code in their coding scheme, which effectively improved the coding potential to 1.58 bits/nt [11]. Before transcoding into DNA nucleotides, binary data was first converted into ternary Huffman code and then transcoded to DNA sequence by referring to a rotating encoding table (Fig. 1B). Every Byte of the resulting data would be substituted by five or six ternary digits (comprises “0”, “1”, “2” only), which can prevent generating mononucleotide repeats and compress the original data by 25% to 37.5%. Besides, for ASCII text format files, compression further outperforms by mapping the most common characters to five-digits ternary strings [11]. In addition, this coding scheme employs simple parity-check coding for error detection and maintains a four-fold coverage redundancy to prevent error and data loss (Fig. 2A). Nevertheless, it should be noted that the simple parity-check coding can only detect the errors, but doesn’t correct them. Moreover, the increased redundancy inevitably lowers the coding efficiency.

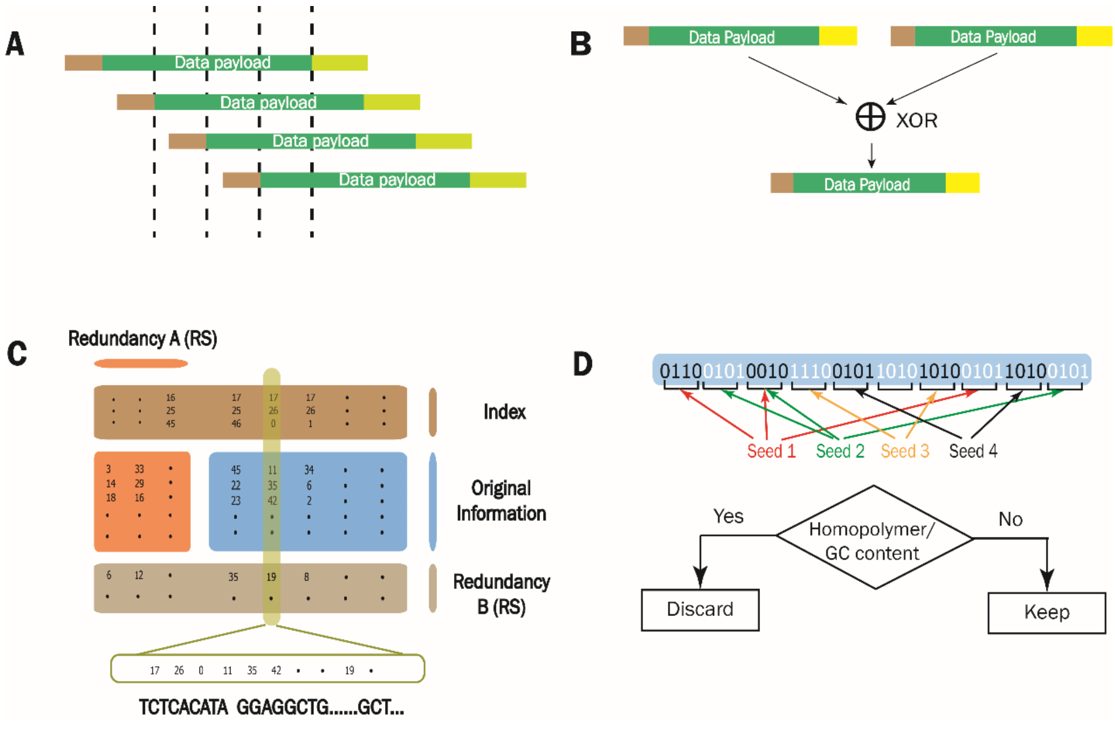

Figure 2. The different redundancy types used in the DNA-based data storage schemes: A) Increasing redundancy by repetition; B) Increasing redundancy by an exclusive-or (XOR) calculation; C) Increasing redundancy by using Reed-Solomon code for two rounds; D) Increasing redundancy by using fountain code.

- Improved Huffman coding scheme

In 2016, Bornholt et. al improved Goldman's encoding scheme by an XOR encoding principle [12], which employed an exclusive-or (XOR, '⊕') operation to yield redundancy. As shown in Fig. 2B, every two original sequences, A and B, will generate a redundant sequence C by A⊕B. Therefore, with any two sequences (AB, AC or BC), one can easily recover the third sequence. Moreover, this coding scheme also provides the flexibility in providing redundancy according to the level of significance of particular data strands, namely "tunable redundancy". This coding scheme successfully encodes 4 files with the total size of 151 KB and recovers 3 out 4 files without manual intervention [12].

Moreover, the need for amplifying target files in a large-scale database suggests the necessity of random-access in DNA-based data storage. Therefore, in 2018, the same team put forward

another error-free coding scheme that allowed the users to randomly reach and recover individual files in a large-scale system. In this coding scheme, unique polymerase chain reaction (PCR) primers are assigned to individual files after rigorous screening, therefore, it allows users to randomly access their target file(s). A total of 200 MB data was successfully stored and recovered in their study, which set a new milestone by complementing the feasibility of storing large-scale data in DNA [13].

- A coding scheme based on Galois Field and Reed-Solomon Code

With special emphasis on error detection and correction, a coding scheme based on the Galois field and Reed-Solomon (RS) code [14] was proposed by Grass and colleagues in 2015 [15]. Meanwhile, the potential data density was improved to ~1.78bits/nt. With the two-byte (8×2 bits) fundamental information block, this coding scheme introduced a finite field (Galois field or GF) of DNA nucleotide triplets as its elements (Fig. 1C). To prevent mononucleotide repeat > 3nt during encoding, the last two nucleotides of the triplet are varied, which can give 48 different triplets. They indeed employed a GF (47), as 47 is the largest prime number smaller than 48. The information block is then mapped to the three elements in GF (47), *i.e.* 256^2 to 47^3. In order to conduct error detection and corrections, RS code is applied in this scheme. As shown in Fig. 2C, two rounds of RS coding are applied horizontally and vertically to the matrix generated by GF transcoding respectively.

In this pilot study, 83 kilobytes of text data were encoded *in silico* [15]. Although the data size was not quite impressive, it underlined the necessity of applying error-correction coding and significantly enhanced the coding efficiency.

- A "forward error correction" coding scheme

Blawat and colleagues proposed a coding scheme to particularly tackle the errors generated during DNA sequencing, amplification and synthesis (*e.g.* insertion, deletion and swapping). The potential coding density was 1.6 bits/nt. Two reference coding tables are specified in advance. The one-byte (8 bits) fundamental information block is assigned to a 5 nt DNA

sequence and the 3rd and 4th nucleotide are swapped (Fig. 1D). The two other criteria are also applied to prevent mononucleotide repeat during this process: 1) the first three nucleotides should not be the same; 2) the last two nucleotides should not be the same. Consequently, an 8-bits data block (*i.e.* 2^8 = 256 permutations for binary data) is transcoded into 704 different DNA blocks (4^5- 4^3- 4^4) [16]. They can be categorized into three clusters: clusters A & B of complete blocks (256 each), and cluster C of 192 incomplete blocks. Data can then be mapped to the DNA blocks A and B as required, e.g. alternately mapped to A or B.

In their study, 22 Mb of data was successfully encoded and stored in an oligo pool. The data were retrieved without any error, thereby proving the feasibility of "forward error correction" coding scheme. But this was not the case for detecting and correcting single-mutation. For example, "11100011" could be mapped to a DNA block "TGTAG". However, if an A-to-T transversion occurs, the DNA block will be changed to "TGTTG", which will give an error byte "11101111" after decoding.

- Fountain code-based DNA-based data storage coding scheme

In 2017, Erilich and Zielinski employed fountain code in their coding scheme [17]. Fountain code is a widespread coding method of the information communication system, and is well known for its robustness and high efficiency [18]. Fountain code is also known as a rateless erasure code, in which data to be stored is divided into *k* segments, namely resource packets. A potentially limitless number of encoded packets could be derived from the resource packets. When it returns *n* ($n > k$) encoded packets, the original resource data will be perfectly recovered. In practice, *n* only need to be slightly larger than *k* to yield a greater coding efficiency as well as robustness for the information communication [19].

Similarly, binary data-nucleotide sequence encryption is also carried out. A fundamental two-bit to one-nucleotide transcoding table is adopted, in which [00, 01, 10, 11] is mapped to [A, C, G, T], respectively (Fig. 1A). At first, original binary information is segmented to small blocks. These blocks are chosen according to a pre-designed pseudorandom sequence of

numbers. A new data block is then created by the bitwise addition of the selected blocks with random seeds attached and transcoded to nucleotide blocks according to the transcoding table. Mononucleotide repeats and abnormal GC content are prevented by a final verification (Fig. 2D) [17].

The oligos in this coding scheme are correlated and have grid-like topology to realize extremely low but necessary redundancy. Their study increased the theoretical limit of coding potential to an unprecedentedly high value of 1.98bits/nt and remarkably reduced the desired redundancy for an error-free recovery of the source file. Moreover, the mechanism of random selection and validity verification ensured that long single-nucleotide homopolymers would not appear in the encoded sequence. However, in this coding scheme, the complexity level of encoding and decoding is not linearly correlated to the data size. Thus, decoding could be complicated and may require more resource and longer time for computation. However, although it is claimed that a 4% loss of total packets would not affect the recovery of the original file in the report, in terms of the features of DNA Fountain code, loss of more packet may cause the complete failure of recovery. If the ultimate aim is to permanently store the data, the amount of redundancy must be raised to ensure the information integrity.

If we solely consider DNA-based data storage as a storage process with high fidelity, the DNA fountain coding appears to be the one and only communication-based coding scheme. In DNA-based data storage and retrieval, the most common error is caused by a single nucleotide mutation. To address this issue, the most coding schemes will create high redundancy in order to tackle the battered condition of current communication channels, however, these error correction algorithms require complex decoding procedures and much computing time. Here, fountain-coding scheme firstly shows that it is unnecessary to employ error detection/correction algorithms, which provide us with an alternative solution towards improving the performance of DNA coding.

Overview of DNA-based data storage mediums:

Currently, the DNA-based data storage employs different mediums to store the encoded DNA sequences. There are mainly two types of storage mediums: *in vivo* and *in vitro*.

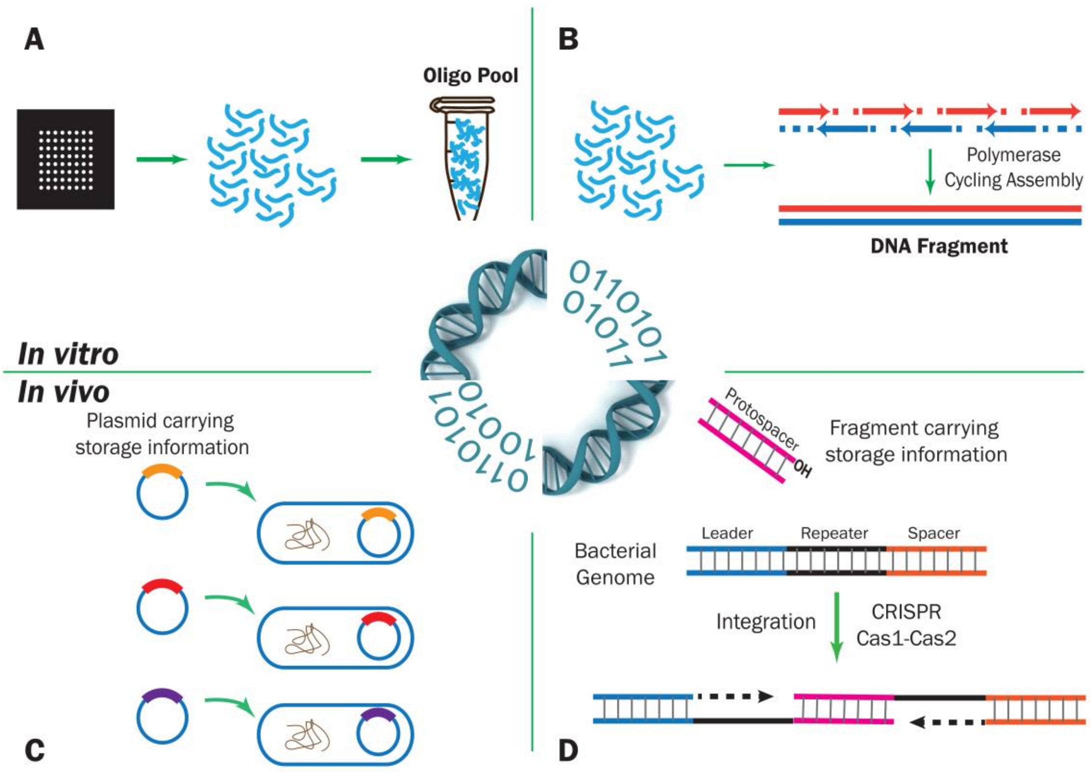

Figure 3. Two categories of DNA-based data storage application. Panel A) and B) demonstrate the two ways of *in vitro* DNA-based data storage; panel C) and D) demonstrate two ways of *in vivo* DNA-based data storage. A) Chip-based high throughput DNA oligo analysis. DNA oligos carrying digital information are stored in the form of oligo pool. B) DNA fragments synthesized by polymerase cycling assembly (PCA), the fragments will carry the information to be stored. C) Digital information inserted into a plasmid and then the plasmids are transferred into bacterial cells. D) DNA fragments carrying digital information is inserted into bacterial genome by employing the CRISPR system using Cas1-Cas2 integrase.

In vivo DNA-based data storage

In vivo DNA-based data storage was a commonly adopted approach in the pioneering works of DNA-based data storage, such as the *Microvenus* project, which used bacteria as the storage medium [2]. Typically, encoded DNA sequences are first cloned into a plasmid and

then transferred into the bacteria. Therefore, the DNA sequences and so does the information it carries can be maintained in the tiny bacteria and their billions of descendants.

Nevertheless, the capacity of bacteria for carrying plasmid is limited by the type of plasmids and their corresponding size. In addition, the mutation of plasmid in bacteria is quite common. During bacterial replication, the spontaneous mutation may ultimately alter the information stored in them after few years.

Recently, Church *et. al* demonstrated a novel method to encode an image and a short movie clip into the bacterial genome using the CRISPR-Cas system with Cas1-Cas2 integrase [20]. Although it is reported that the CRISPR-Cas system is not equally efficient to all the sequences, this work greatly improved the capability of *in vivo* DNA-based data storage.

In vitro DNA-based data storage

Apart from *in vivo* DNA-based data storage, the *in vitro* DNA-based data storage is seen more frequently in the recent studies. One of the most popular form is the oligo library. This is largely due to the maturation of chip-based high-throughput oligo synthesis technique [21], making the synthesis of a large amount of DNA oligos more cost-effective.

During the synthesis process, each oligo is assigned a short tag, or index, as all the oligos are completely mixed for high throughput synthesis and sequencing. Current oligo synthesis technique is able to generate at most 200-mers with relatively high accuracy and purity [22]. Hence, the index should be as short as possible to save the information capacity in each oligo. Apparently, much more indices will be needed if more DNA oligo sequences are generated and mixed. However, similar to *in vivo* DNA-based data storage, the larger data size demands more DNA oligos for *in vitro* DNA-based data storage. This increases the size of indices in an oligo and thus lower the storing capacity and efficiency.

To overcome these problems, longer DNA fragments can be used instead of DNA oligos. In 2017, Yadzi *et. al* successfully encoded 3633 bytes of information (two images) into 17 DNA

fragments and recovered the image using homopolymer error correction [23]. Nevertheless, the current cost of DNA fragment synthesis is higher than that of oligo synthesis, which increases the overall cost of DNA fragment-based storage.

Some other pioneering work also goes beyond our aforementioned DNA-based data storage system. Song and Zeng proposed a strategy which is claimed to be able to detect and correct error in each byte [24]. They transformed a short message into *E.coli* stellar competent cells and proved the reliability of their strategy. Lee *et. al* have incorporated enzymatic DNA synthesis and DNA-based data storage principles, reporting an enzymatic-based DNA-based data storage strategy [25]. All this research has laid a sound foundation for the global application of this novel storage medium.

Challenges of DNA-based data storage

The limited size of synthetic DNA

As mentioned above, the information encoded in DNA depends on DNA synthesis. While DNA oligos usually serve as the basic building blocks for gene synthesis, the DNA synthesis often includes oligo synthesis (≤ 200 mer) and gene synthesis (200-3,000 bp or above) depending upon the final product size., For cost saving purposes and to reduce the complexity of DNA synthesis, primary storage unit size is often limited below 200nt [21].

Due to this lower limit, information needs to be fragmented and indexed before encoding into DNA to allow oligo synthesis (encoding) and pool sequencing (decoding) to reconstruct data in the correct order. Thus, when the amount of information grows, not only do the number of fragments increase, but the indexing information also accumulates subsequently. Except for optimizing the index length (see “The future of DNA-based data archiving” below), techniques for synthesizing longer oligo are considered to be the major challenge before we can push the envelope.

DNA sequencing-induced errors

Currently, there are two major types of DNA sequencing techniques: real-time, single-molecule sequencing and massively parallel (or next generation) sequencing. The latter is a high-throughput sequencing method and is dominant for short-read (<700bp, depending on the platform) sequencing while the former is on the opposite [9,26].

In DNA-based data storage, massively parallel sequencing is widely used for data retrieval ever since it was first employed by Church *et al*. in 2012. Two main reasons can explain this prevalence. Firstly, the length of the synthetic DNA generated from encoding is relatively short, meaning it is more cost-effective to sequence with massively parallel sequencing. Secondly, the throughput and accuracy (~99.9%) of massively parallel sequencing still far surpass its counterparts [9]. However, this technique also comes with a limitation. Most massively parallel sequencing platforms require an *in vitro* template amplification with primers, to generate a complex template library for sequencing. During this process, copying errors, sequence-dependent biases (for example, in high- and low-GC regions and at long mononucleotide repeats) and information loss (for example, methylation) are produced [9].

Nevertheless, sequencing with minimal biases and random errors in respect to accuracy and contiguity is possible, given that rapid progress is now achieved in real-time, single-molecule sequencing. It is reported that this rising technique can tolerant high GC content and only generates random errors [27], which is ideal in data retrieval. So, once it also achieves the high-fidelity, the storage potential of DNA may be further unlocked.

Other considerations regarding DNA sequencing

Apart from the accuracy, the speed and the total cost of DNA sequencing are also major considerations. Table 1 summarizes the frequently-used sequencing platforms in DNA-based data storage. We can see that sequencing is still costly and time-consuming. One less frequently mentioned reason is that although the core sequencing process is automated, there

are manual steps in between (e.g. sample preparation), which significantly slow down the process. Therefore, a higher level of automation may help to speed up the run and also bring down the cost per Gb.

Interestingly, this table also shows that an error-prone sequencing platform-Oxford Nanopore MinION has become increasingly popular. This is probably due to its potential for high-compactness and stand-alone DNA data storage systems [13, 29], although the emergence of a growing number of error-tolerant coding schemes is also a contributor [13, 30]. This year, Oxford Nanopore also launched a high-throughput sequencing platform-PromethION, which has the potential to yield up to 15 Tb of data in 48 hours [31]. As its performance is getting closer to its next-generation sequencing counterparts, it may play a bigger role in the future study of DNA-based data storage.

Platform	Error Rate	Runtime	Instrument Cost(US$)	Cost per Gb (US$)	Reference
Illumina MiSeq	0.1%	4-56h*	$99K	$110-1000*	[12]Bornhol et al.,2016<br>[15]Grass et al.,2015<br>[17]Erlich Y and Zielinski D,2017<br>[20]Shipman et al.,2017
Illumina HiSeq 2000	2.0%	3-10d *	$654K	$41	[8]Church et al.,2012<br>[11]Goldman et al.,2013
Illumina HiSeq 2500	0.1%	7h-11d *	$690	$30-230*	[16]Blawat et al.,2016
Illumina NextSeq	0.1%	11-29h*	$250	$33-43*	[13]Organick et al.,2018
Oxford Nanopore MinION	12.0 %	up to 48h	$1K	$750	[13]Organick et al.,2018<br>[23]Yazdi et al.,2017

d, days; Gb, gigabase pairs; h, hours; K, thousand; * varied by read length and version of the reagent kit

Table 1. The summary of frequently-used sequencing platforms in DNA-based data storage (data retrieved from [26]).

The future of DNA-based data archiving

Taken together, DNA-based data storage provides us the immense possibility to manipulate DNA as a carbon-based archive with an excellent storage density and stability. Imperfect as it is, it might be the ultimate solution to the current data storage market for long-term archiving. We are also excited to see that multidisciplinary research companies have already joined this revolution to make DNA-based archiving as a commercially viable approach.

Enterprises with a strong DNA-synthesis background are most commonly seen, given that DNA-based data storage can significantly benefit from the breakthroughs achieved in DNA synthesis. It could be foreseen that with the continuously improving enzymatic DNA synthesis technique, DNA oligo synthesis could break the limitation of 200-mers in the near future, providing us longer primary storage unit. This will undoubtedly improve the net coding efficiency with the same length of PCR primer and shorter index sequences. A modelling was performed for DNA-based data storage of 1GB file under theoretical limitation, where one DNA base represented two binary bits. For each DNA oligo, the length of forward and reverse primers was set as 20. In this case, we can deduce the equation representing the relationship between index length i and DNA oligo length l: $log_2(l-40-i)+i=32$ (Equation 1). Hence, we could get the correlation between an optimal index length and DNA oligo length.

As Figure 4 shows, with the increase in DNA oligo length, the index length also decreases, while net coding efficiency increases. It is reported that some startup companies around the world are now aiming to develop industrial enzymatic DNA synthesis technology. If they can successfully synthesize oligos over 200-mers, the efficiency of DNA-based data storage is expected to be remarkably improved.

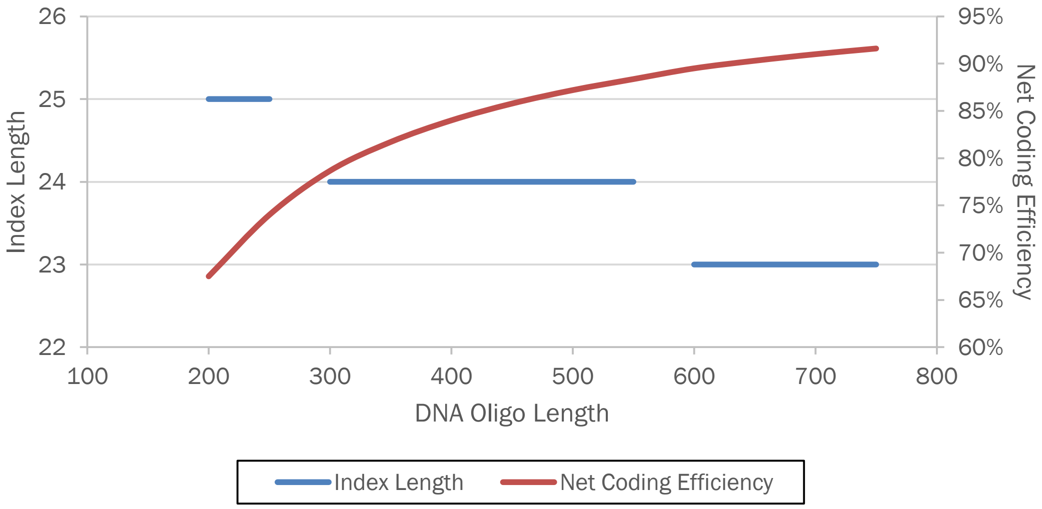

Figure 4 The inter-relationship between DNA oligo length, the optimal index length and net coding efficiency during the modelling of 1GB digital file transcoding.

In addition, the scale of DNA synthesis also affects the information capacity of DNA-based data storage per unit mass. High-throughput oligo synthesis is currently directed to microscale level with the development of chip-based DNA synthesis technology. In DNA-based data storage, the information capacity of a certain mass of DNA sequences also relates to the copy number of each DNA molecule. The correlation between information capacity C and copy number N_m of each oligo can be calculated from: $C = n \times (N_m \mu \delta \gamma)^{-1}$ (Equation. 2) where n represents the number of bytes each oligo carries, normally 10 – 20 bytes/molecule according to different coding schemes; μ is the number of nucleotides per molecule, δ is 320 Dalton/nucleotide; γ is 1.67×10^{-24} g/Dalton. To date, the copy number of oligos is around 10^7 molecules in the microchip-based high throughput synthesis (without dilution) [17] and according to the Equation (2), this will give an information capacity level of ~10^{13} bytes/g. If the copy number is decreased to 10^4 molecules per oligo, the information capacity will increase to ~10^{16} bytes/g. Additionally, synthesis in microscale will also reduce the cost by several orders of magnitude and save the dilution step.

At present, several DNA synthesis companies are taking the lead on this field based on their related expertise, and providing services related to DNA-based data storage. It is reported that

Twist Biosciences has already collaborated with Microsoft in their DNA-based data storage project, providing them oligo pool services [18], with their high-throughput, chip-based DNA synthesis technique. Given that these companies are starting to push this business forward, it will be interesting to see how commercial applications develop in the future.

Apart from companies with biological backgrounds, IT-based industries are also playing an important role in this revolution. As the coding schemes used in DNA-based data storage still need to be improved to yield higher coding efficiency and fidelity, efforts from the IT field could be of critical importance. For example, from random access data retrieval to scaling up data storage [13], Microsoft successfully implement its IT philosophy in DNA-based data storage and is marching steadily towards its goal announced in 2017: a proto-commercial system in three years storing some amount of data on DNA [32]. In its recent paper collaborated with a scientist from the University of Washington, an automated end-to-end DNA-based data storage device was described and 5-bytes of data were automatically processed by the write, store, and read cycle [29]. Further efforts that can speed up the coding and decoding process for daily storage applications are still essential.

In addition, we are expecting to see a lot more entities and research organizations to join this cohort in eventually making the carbon-based archiving a reality and go further to reach the fields of immediate access storage (IAS) or the biological computation. Nevertheless, keeping DNA-based data storage development under a safe and ethical framework is still of foremost priority. Since DNA is the basic building block of genetic information for living organisms, there might be situations where synthesized sequences are being introduced into host living organisms that might lead to biological incompatibility due to unknown toxicity or other growth stresses to host organisms. Hence, it is necessary to evaluate the safety of the sequences prior to its synthesis. We are craving to see the day when DNA become the next-generation digital information storage media with high safety, capacity and reliability.

Acknowledgements

This work was supported by Guangdong Provincial Academician Workstation of BGI Synthetic Genomics (No. 2017B090904014).

References

1. Neiman MS. Some fundamental issues of microminiaturization. *Radiotekhnika*. 1964;No. 1:3-12.
2. Joe Davis a. Microvenus. Art Journal. 1996; 1:70. doi:10.2307/777811.
3. Bonnet J, Colotte M, Coudy D, Couallier V, Portier J, Morin B, *et a*l. Chain and conformation stability of solid-state DNA: implications for room temperature storage. *Nucleic Acids Research*. 2010;38 5:1531-46. doi:10.1093/nar/gkp1060.
4. Pääbo S, Poinar H, Serre D, Jaenicke-Després V, Hebler J, Rohland N, *et al*. GENETIC ANALYSES FROM ANCIENT DNA. *Annual Review of Genetics*. 2004;38:645-79. doi:10.1146/annurev.genet.37.110801.143214.
5. Kool ET. Hydrogen bonding, base stacking, and steric effects in DNA replication. Annual Review of Biophysics & Biomolecular Structure. 2001;30 1:1-22.
6. Nelson DL, Cox MM and Lehninger AL. *Lehninger principles of biochemistry*. New York ; Basingstoke : W.H. Freeman, c2008. 5th ed.; 2008
7. Pierce BA. Genetics : a conceptual approach. New York, NY : W.H. Freeman, c2012. 4th ed., International ed.; 2012.
8. Church GM, Gao Y and Kosuri S. Next-generation digital information storage in DNA. *Science*. 2012;337 6102:1628. doi:10.1126/science.1226355.
9. Shendure J, Balasubramanian S, Church GM, Gilbert W, Rogers J, Schloss JA, et al. DNA sequencing at 40: past, present and future. *Nature*. 2017;550 7676:345-53. doi:10.1038/nature24286.
10. De Silva PY and Ganegoda GU. New Trends of Digital Data Storage in DNA. *Biomed Res Int*. 2016;2016:8072463. doi:10.1155/2016/8072463.
11. Goldman N, Bertone P, Chen S, Dessimoz C, LeProust EM, Sipos B, et al. Towards practical, high-capacity, low-maintenance information storage in synthesized DNA. *Nature*. 2013;494 7435:77-80. doi:10.1038/nature11875.

12. Bornholt J, Lopez R, Carmean DM, Ceze L, Seelig G and Strauss K. A DNA-Based Archival Storage System. *SIGPLAN Not*. 2016;51 4:637-49. doi:10.1145/2954679.2872397.

13. Organick L, Ang SD, Chen YJ, Lopez R, Yekhanin S, Makarychev K, et al. Random access in large-scale DNA data storage. *Nat Biotechnol*. 2018;36 3:242-8. doi:10.1038/nbt.4079.

14. Reed I and Solomon G. Polynomial Codes Over Certain Finite Fields. *Journal of the Society for Industrial and Applied Mathematics*. 1960;8 2:300-4. doi:10.1137/0108018.

15. Grass RN, Heckel R, Puddu M, Paunescu D and Stark WJ. Robust chemical preservation of digital information on DNA in silica with error-correcting codes. *Angew Chem Int Ed Engl*. 2015;54 8:2552-5. doi:10.1002/anie.201411378.

16. Blawat M, Gaedke K, Hütter I, Chen X-M, Turczyk B, Inverso S, et al. Forward Error Correction for DNA Data Storage. *Procedia Computer Science*. 2016;80:1011-22. doi:10.1016/j.procs.2016.05.398.

17. Erlich Y and Zielinski D. DNA Fountain enables a robust and efficient storage architecture. *Science*. 2017; 6328:950. doi:10.1126/science.aaj2038.

18. Byers JW, Luby M, Mitzenmacher M and Rege A. A digital fountain approach to reliable distribution of bulk data. *Proceedings of the ACM SIGCOMM '98 conference on Applications, technologies, architectures, and protocols for computer communication*. Vancouver, British Columbia, Canada: ACM, 1998, p. 56-67.

19. MacKay DJ. Fountain codes. *IEE Proceedings-Communications*. 2005;152 6:1062-8.

20. Shipman SL, Nivala J, Macklis JD and Church GM. CRISPR-Cas encoding of a digital movie into the genomes of a population of living bacteria. *Nature*. 2017;547 7663:345-9. doi:10.1038/nature23017.

21. Kosuri S and Church GM. Large-scale de novo DNA synthesis: technologies and applications. *Nature methods*. 2014;11 5:499-507. doi:10.1038/nmeth.2918.

22. Ma S, Tang N and Tian J. DNA synthesis, assembly and applications in synthetic biology. *Curr Opin Chem Biol*. 2012;16 3-4:260-7. doi:10.1016/j.cbpa.2012.05.001.

23. Yazdi S, Gabrys R and Milenkovic O. Portable and Error-Free DNA-Based Data Storage. *Sci Rep*. 2017;7 1:5011. doi:10.1038/s41598-017-05188-1.

24. Song L and Zeng AP. Orthogonal Information Encoding in Living Cells with High Error-Tolerance, Safety, and Fidelity. *ACS Synth Biol*. 2018;7 3:866-74. doi:10.1021/acssynbio.7b00382.

25. Lee HH, Kalhor R, Goela N, Bolot J and Church GM. Enzymatic DNA synthesis for digital information storage. *bioRxiv*. 2018; doi:10.1101/348987.

26. Goodwin S, McPherson JD and McCombie WR. Coming of age: ten years of next-generation sequencing technologies. *Nat Rev Genet*. 2016;17 6:333-51. doi:10.1038/nrg.2016.49.

27. Ross MG, Russ C, Costello M, Hollinger A, Lennon NJ, Hegarty R, *et al*. Characterizing and measuring bias in sequence data. *Genome Biol*. 2013;14 5:R51. doi:10.1186/gb-2013-14-5-r51.

28. van Dijk EL, Jaszczyszyn Y, Naquin D and Thermes C. The Third Revolution in Sequencing Technology. *Trends Genet*. 2018;34 9:666-81. doi:10.1016/j.tig.2018.05.008.

29. Takahashi CN, Nguyen BH, Strauss K and Ceze LH. Demonstration of End-to-End Automation of DNA Data Storage. bioRxiv. 2018; doi:10.1101/439521.

30. De Coster W, De Roeck A, De Pooter T, D'Hert S, De Rijk P, Strazisar M, et al. Structural variants identified by Oxford Nanopore PromethION sequencing of the human genome. bioRxiv. 2018; doi:10.1101/434118.

31. PromethION. https://nanoporetech.com/products/promethion.

32. Antonio Regalado. Microsoft Has a Plan to Add DNA Data Storage to Its Cloud, MIT Technology Review, 2017.

Figure 1 Click here to access/download;Figure;Figure 1.tif

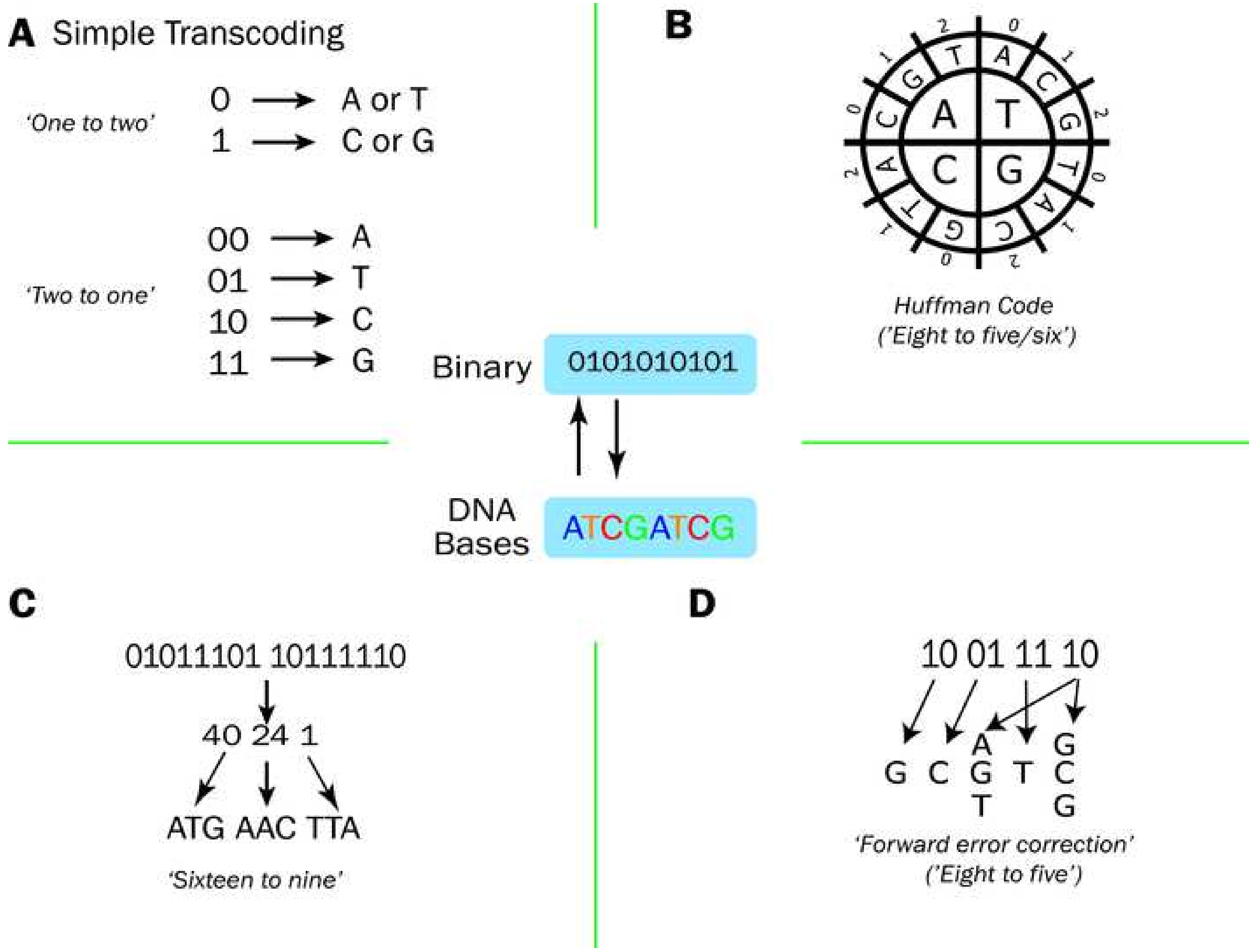

Figure 2

Click here to access/download;Figure;Figure 2.tif

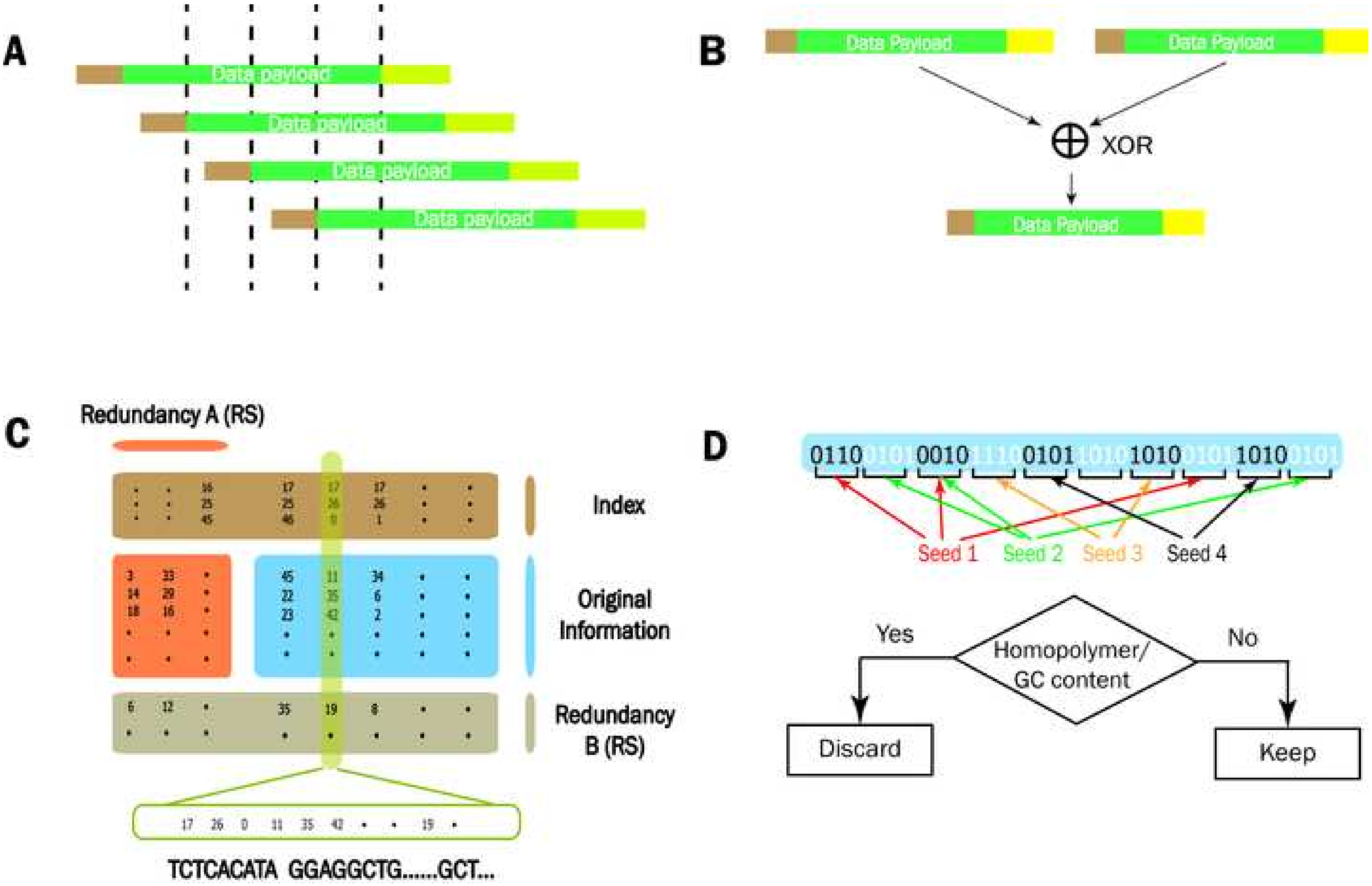

Figure 3

Click here to access/download;Figure;Figure 3.tif

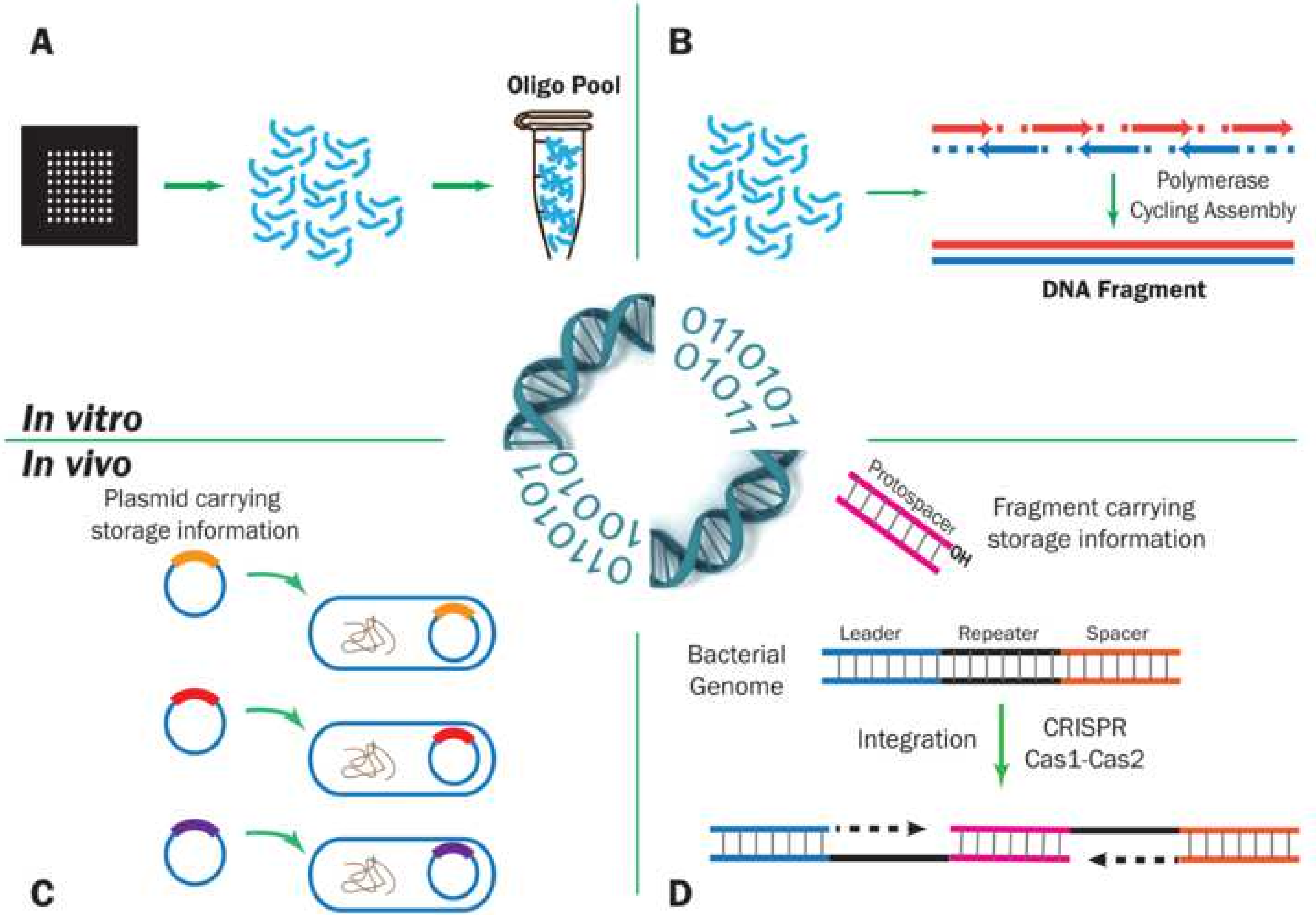

Figure 4

Click here to access/download;Figure;Figure 4.tif

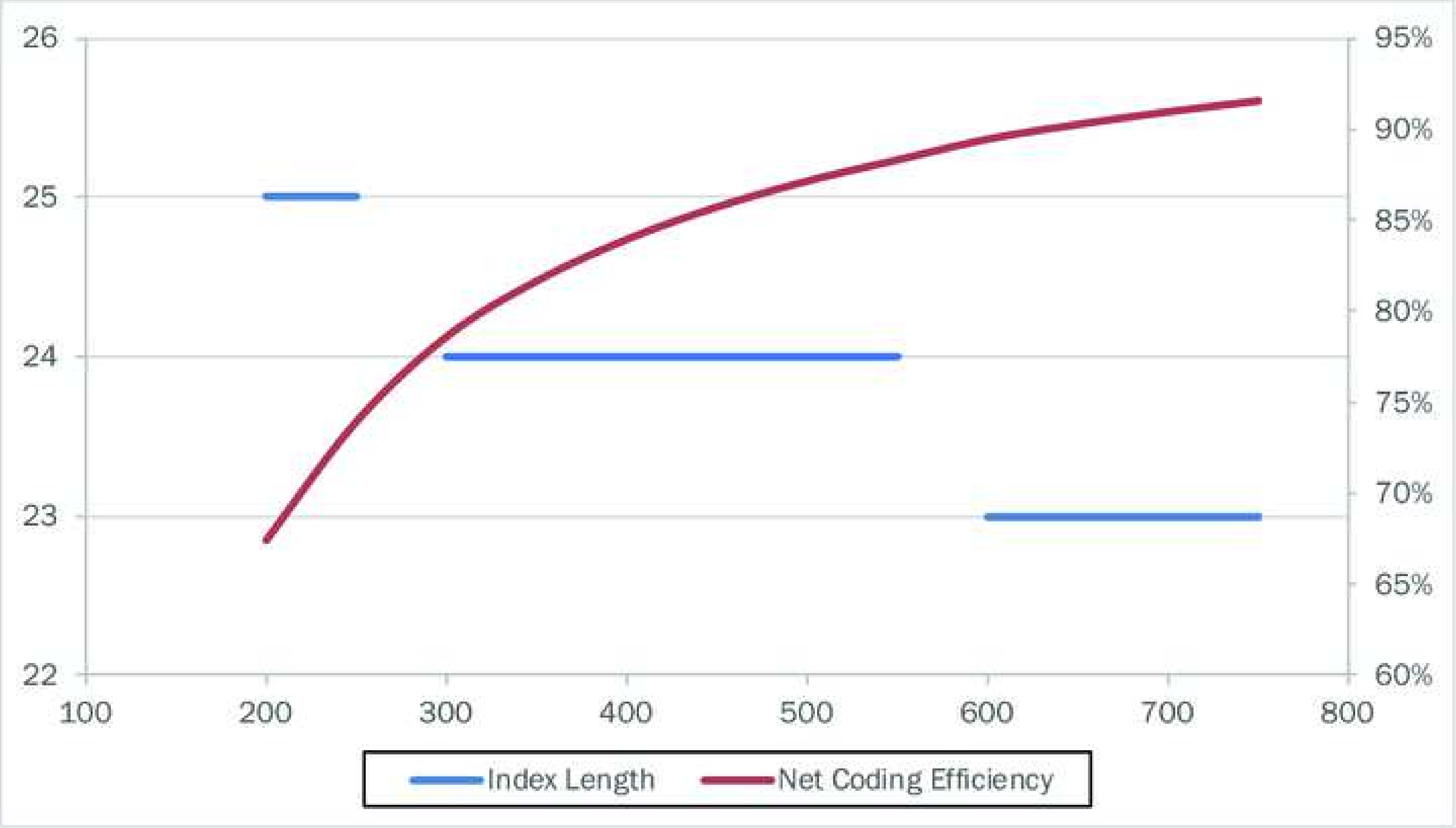

Cover letter

Click here to access/download;Personal Cover;Cover letter (Carbon-based Archiving).docx

Dear Editor of GigaScience:

We submit our manuscript entitled “Carbon-based archiving: the current progress and future prospects of DNA-based data storage" to GigaScience for publication.

This manuscript is a review of DNA-based storage with focus on current progress summary on coding scheme and media type. We provide scalable measurements and technical opinions of this field, which we believe will be a great add on to people’s current understanding and help promote its better development. As DNA-based storage is a promising bio-approach for large scale and long term digital information storage, we consider it is well in scope of the GigaScience’s publication criteria.

All authors have read and have abided by the publication ethics as set out by the Commission on Publication Ethics (COPE) for manuscripts submitted to GigaScience.

All authors declared that they have no conflicts of interest to this work.

The work described has not been submitted elsewhere for publication, in whole or in part, and all the authors listed have approved the manuscript that is enclosed.

Thank you very much for your attention and consideration.

Yours sincerely,

Yue (Chantal) Shen

Sha Joe Zhu
