# Supplementary material for: Carbon-based archiving: current progress and future prospects of DNA-based data storage
Source: Gigascience. 2019 Jun 20;8(6):giz075. doi: 10.1093/gigascience/giz075 (PMC6586197; doi:10.1093/gigascience/giz075)
Supplement: giz075_GIGA-D-18-00466_Revision_2 [file giz075_giga-d-18-00466_revision_2.pdf]

GigaScience

Carbon-based archiving: the current progress and future prospects of DNA-based data storage

--Manuscript Draft--

**Manuscript Number:**	GIGA-D-18-00466R2
**Full Title:**	Carbon-based archiving: the current progress and future prospects of DNA-based data storage
**Article Type:**	Review
**Funding Information:**	Guangdong Provincial Academician Workstation of BGI Synthetic Genomics (2017B090904014) — Dr. YUE SHEN<br>Guangdong Provincial Key Laboratory of Genome Read and Write (2017B030301011) — Dr. YUE SHEN<br>Shenzhen Engineering Laboratory for Innovative Molecular Diagnostics (DRC-SZ[2016]884) — Dr. YUE SHEN
**Abstract:**	The information explosion has led to a rapid increase in the amount of data to be physically stored. However, the existing storage method (magnetic and optical media) will not be sufficient to store this exponentially growing data in the near future. Therefore, data scientists are continuously looking for better alternatives to store these hefty amounts of data in a space-efficient and stable fashion. Because of its unique biological properties, the highly densed "DNA" holds a great potential to become the future storage material. In fact, DNA-based data storage has recently emerged as a promising approach for long-term digital information storage. This review summarizes the state-of-the-art methods including digital-to-DNA coding schemes and the media types used in DNA-based data storage, and provide a general overview of the most recent progress achieved in this field and its exciting future.
**Corresponding Author:**	YUE SHEN<br><br>CHINA
**Corresponding Author Secondary Information:**	
**Corresponding Author's Institution:**	
**Corresponding Author's Secondary Institution:**	
**First Author:**	Zhi Ping
**First Author Secondary Information:**	
**Order of Authors:**	Zhi Ping
	Dongzhao Ma
	Xiaoluo Huang
	Shihong Chen
	Longying Liu
	Fei Guo
	Sha Joe Zhu
	YUE SHEN
**Order of Authors Secondary Information:**	
**Response to Reviewers:**	Response to Reviewer #1:<br><br>Dear Reviewer #1,

Thank you for sparing your valuable time and providing useful suggestions. We have read your comments and made changes to the manuscript accordingly. We have carefully revised the manuscript, and we have also reconstructed some of the sections and added some new information as per your suggestions. Our specific responses are as follows:

1)[Page 3, Lines 9-22]: A 4th unique feature of DNA that might be included is the ease and rapidity with which DNA can be replicated using, for example, PCR.

Response: We thank you for your valuable suggestion. As the PCR technique is now well-developed and cost-effective, the replication of DNA sequences encoding digital files are easy and efficient. Meanwhile, for in vivo DNA storage, living cells could also replicate rapidly as long as it is active and has sufficient food supply. Therefore, the convenience of file replication and a backup would be another unique feature for DNA-based data storage. The description of this feature is mentioned in [Page 3, Lines18-21].

2)[Page 4: Line 8]: The authors write "there is a trade-off between accuracy and redundancy". In my interpretation, this is counter-intuitive, as additional redundancy should reduce errors.

Response: We thank you for your useful suggestion. The additional redundancy, including error-correction codes, are designed to ensure the fidelity of DNA-based data storage. However, the redundancy will use resources (i.e. bases in DNA sequence) and thus reduce the coding density. That is why we mentioned about “the trade-off between accuracy and redundancy”. To clarify this opinion, we have elaborated the details in [Page 4, Lines 10-14].

3)[Page 4: Lines 13-15]: Concerning random access, many experimental works demonstrating DNA data storage do not have random access. Thus it may not necessarily be a requirement. Can the authors discuss this further?

Response: We thank you for your valuable suggestion. It is true that many experimental works did not consider random access in DNA-based data storage. However, in the large-scale orthodox storage system (e.g. computer system), random access is one of the most basic features for data retrieval. As a result, the research team from the University of Washington and Microsoft reported the significance of their work on random access for DNA-based data storage. We emphasize the importance of random access in [Page 4, Lines 17-19].

4)[Page 12: lines 5-6]: The amount of time is less informative than citing a number of bacterial divisions/replications over which the data is expected to mutate significantly.

Response: We thank you for your useful suggestion. The spontaneous mutation rate in bacterial replication is extremely low depends on the form of storage. The related statements are added in [Page 12, Lines 11-15].

5)[Pages 11-12]: Concerning in-vivo storage, the authors fail to cite a number of early works in DNA data storage that included an in-vivo storage component. For instance: Bancroft 2001, Wong 2003, and Arita 2004.

Response: We thank you for the nice suggestion. The early reference of in vivo DNA-based data storage has been cited accordingly. These works are mentioned in [Page 3, Lines 8-9 and Page 12, Lines 3-5].

6)[Pages 11-12]: The authors might also want to mention other methods of storing data in vivo, for instance with recombinases, and other molecular recorders like Cas9.

Response: We thank you for the useful suggestion. In some recent works, molecular tools like CRISPR-Cas has been described for writing information in vivo. The corresponding work is mentioned in [Page 12, Lines 15-18]. Similarly, the possible application of CRISPR and recombinase in DNA-based data storage is mentioned in [Page 14, Lines 8-10].

7)[Page 13, lines 23-25]: Is length really the major challenge? Why not just write-throughput in general, which can be increased by synthesis of longer strands (as stated), and/or by writing more strands in parallel (which is not mentioned) for instance by making larger, more dense oligo synthesis arrays.

Response: We thank you for your valuable suggestion. We consider oligo length as one of the major challenges because in DNA-based data storage, in order to retrieve the data, we need indices (e.g. 1,2,3…) to record the address of oligo in a pool of oligo

mixture. With the increase in file size , more oligo will be needed and thus larger indices. Therefore, the index region in a data-encoded DNA sequence would be longer and reduce the coding efficiency. With longer oligo length, the number of oligos required to store a file with the same size will be reduced and thus the length of the index region. The explanation of the significance of oligo length is further explained in [Page 13, Lines 2-6].

8)[Page 14, lines 16-20]: This paragraph is confusing, and should be re-written for clarity.
Response: We thank you for highlighting this. This whole section is now re-written accordingly in [Page 14 – Page 17].

9)[Table 1]: Costs for HiSeq2500 and NextSeq are missing "K" symbols.
Response: We thank you for the useful suggestions. The symbols are now added accordingly.

Thank you again for the peer reviewing.

Best wishes,
Yue (Chantal) SHEN, Ph.D.
Genome Synthesis and Editing Platform, China National GeneBank
BGI-Research
Mobile: +86 150 1383 3483
Address: China National GeneBank (CNGB), Jinsha Road, Dapeng District, Shenzhen, Guangdong, China
Mail: shenyue@genomics.cn

Reply to Reviewer #2

Dear Reviewer #2,
Thank you for sparing your valuable time and providing useful suggestions. We have read your comments and made changes to the manuscript accordingly. We have carefully revised the manuscript, and we have also reconstructed some of the sections and added some new information as per your suggestions.
The objective of this manuscript is to help the readers to understand that coding scheme and storage medium are the two major research focus in DNA-based data storage field. Moreover, we would like to also introduce the challenges in current DNA-based data storage, which may inspire the related researchers to have some ideas for further studies. Therefore, for coding schemes, we tried to introduce some key yet well-accepted bit-to-base algorithms and stated their improvement with respect to coding density, the capability of error correction, and capability of random access. Since there are currently no systematic studies on storage media, we introduced some representative works which employed in vivo and in vitro strategy, and showed their comparative description.
Our specific responses are as follows:
1)Page 3, Advantage of using DNA for storage would also include: easy amplification in vivo by live cells at very low cost, and possible amplification in vitro by enzymatic reaction, e.g., PCR or linear amplification in silico. Both approaches can be used to scale up the backup copy production. One should also consider the possible employment of repair system for correcting errors.
Response: We thank you for your precious suggestion. As the PCR technique is now well-developed and cost-effective, the replication of DNA sequences encoding digital files are easy and efficient. Meanwhile, for in vivo DNA storage, living cells could also replicate rapidly as long as it is active and has sufficient food supply. Therefore, the convenience of file replication and a backup would be another unique feature for DNA-based data storage. The description of this feature is mentioned in [Page 3, Lines 18-21].

2)Page 5-10, the description of the coding schemes is quite sketchy. The outline for each approach was brief and was not well illustrated by the panels in the figure 1 and 2. Better schematics may help, without the need to go back to the original papers to make detailed comparison.
Response: We thank you for your valuable suggestion. In this section, we presented the differences between coding schemes by their different bit-to-base transcoding

Powered by Editorial Manager® and ProduXion Manager® from Aries Systems Corporation

	method for optimize coding efficiency and strategies to add redundancy to ensure fidelity. Although we chronologically presented various coding schemes, we tried to deliver the information that all the coding schemes made improvement based on these two ways. In order to further emphasize it, we have stated this opinion just before the description of coding schemes. Furthermore, some more details are given for each coding scheme, as well as the description of our current understanding and perspectives. We reconstructed the figure.1 and corrected the mislabeled figure caption in [Page 5] for better presentation.<br><br>3)Page 10-12, in vivo and in vitro storage of the information - a thorough comparison of the pros and cons would be helpful, instead of factually describing what methodology is available. The error generated in vivo by mutation should be contrasted to the error in DNA synthesis technology to evaluate the limitation of these tools.<br>Response: We thank you for this nice suggestion. The in vivo and vitro storage are two strategies that can be distinguished in many aspects. We compared the pros and cons, and added one additional paragraph to discuss the comparison using a table. This paragraph is in [Page 13, Lines 11-20].<br><br>4)Page 13, line 4-10. A very typical way of this review in describing methodology citing the previous reports without describing the details and contrasting the differences sufficiently. It does not serve the purpose of a proper analysis of how each method advances the development of storage.<br>Response: We thank you for your useful suggestion. Since the concept of DNA digital storage was put forward in 2012, many strategies has been reported. In this review, we mainly reviewed the major event of this field in coding scheme and storage medium aspect, instead of molecular tools, synthesis techniques, etc. Some other strategies, including Song et al., and Lee et al., did not made much improvement in coding efficiency or application demonstration. But they also gave some inspirations for this field of research which should not be neglected in our point of view. Therefore, we mentioned these studies briefly for readers who are interested in this field. In addition, we provided brief opinions instead of mere describing the methodology in this part. The amended paragraph is in [Page 14, Lines 2-10].<br><br>5)Page 14, sequencing accuracy issue was discussed concerning the data retrieval process. While table 1 summarizes the factual information of the technology available, no clear evaluation of the future direction is given, same as pointed out in (4) above.<br>Response: We thank you for your precious suggestion. High-throughput sequencing is one of the most significant tools for DNA-based data storage. We summarized the current techniques by their cost and throughput. We also added some evaluation of the future development direction of the sequencing technique. Besides we also reconstructed the section of “Challenges of DNA-based data storage”. The amended section is in [Page 14, Line 11- Page 17, Line 13].<br><br>6)Fig. 4, the appending figure has no label of Y axis.<br>Response: We thank you for your valuable suggestion. The corrected appending figure has been now re-uploaded accordingly.<br><br>Thank you again for the peer reviewing.<br><br>Best wishes,<br>Yue (Chantal) SHEN, Ph.D.<br>Genome Synthesis and Editing Platform, China National GeneBank<br>BGI-Research<br>Mobile: +86 150 1383 3483<br>Address: China National GeneBank (CNGB), Jinsha Road, Dapeng District, Shenzhen, Guangdong, China<br>Mail: shenyue@genomics.cn
**Additional Information:**	
**Question**	**Response**
Are you submitting this manuscript to a special series or article collection?	No

Powered by Editorial Manager® and ProduXion Manager® from Aries Systems Corporation

**Experimental design and statistics**<br><br>Full details of the experimental design and statistical methods used should be given in the Methods section, as detailed in our Minimum Standards Reporting Checklist. Information essential to interpreting the data presented should be made available in the figure legends.<br><br>Have you included all the information requested in your manuscript?	Yes
**Resources**<br><br>A description of all resources used, including antibodies, cell lines, animals and software tools, with enough information to allow them to be uniquely identified, should be included in the Methods section. Authors are strongly encouraged to cite Research Resource Identifiers (RRIDs) for antibodies, model organisms and tools, where possible.<br><br>Have you included the information requested as detailed in our Minimum Standards Reporting Checklist?	Yes
**Availability of data and materials**<br><br>All datasets and code on which the conclusions of the paper rely must be either included in your submission or deposited in publicly available repositories (where available and ethically appropriate), referencing such data using a unique identifier in the references and in the "Availability of Data and Materials" section of your manuscript.<br><br>Have you have met the above requirement as detailed in our Minimum Standards Reporting Checklist?	Yes

Powered by Editorial Manager® and ProduXion Manager® from Aries Systems Corporation

Manuscript
Click here to access/download;Manuscript;Carbon-base - V8_SCE_PZ_edit 20190603.docx
Click here to view linked References

Carbon-based archiving: current progress and future prospects of DNA-based data storage

Zhi Ping[1,†], Dongzhao Ma[1,†], Xiaoluo Huang[1†], Shihong Chen[1], Longying Liu[1], Fei Guo[1], Sha Joe Zhu[2*], Yue Shen[1*]

[1]Guangdong Provincial Key Laboratory of Genome Read and Write, Shenzhen Engineering Laboratory for Innovative Molecular Diagnostics, Guangdong Provincial Academician Workstation of BGI Synthetic Genomics, BGI-Shenzhen, Shenzhen, 518083, China

[2]Big Data Institute, University of Oxford, Li Ka Shing Centre for Health Information and Discovery, Old Road Campus, Oxford OX3 7LF, UK

[*]Correspondence address: Yue Shen, Guangdong Provincial Key Laboratory of Genome Read and Write, Shenzhen Engineering Laboratory for Innovative Molecular Diagnostics, Guangdong Provincial Academician Workstation of BGI Synthetic Genomics, BGI-Shenzhen, Shenzhen, 518083, China; Tel: [+86-755-36307888]; Email: shenyue@genomics.cn

Sha Joe Zhu, Big Data Institute, University of Oxford, Li Ka Shing Centre for Health Information and Discovery, Old Road Campus, Oxford OX3 7LF, UK; Tel: [+44-0-1865 287770]; Email: sha.joe.zhu@gmail.com

ORCIDs :

Zhi Ping: 0000-0001-7114-1124; Dongzhao Ma: 0000-0002-4606-4339; Shihong Chen: 0000-0003-2999-0426; Sha Joe Zhu: 0000-0001-7566-2787; Yue Shen: 0000-0002-3276-7295

[†]These authors contributed equally to this work.

Abstract

The information explosion has led to a rapid increase in the amount of data requiring physically storage. However, in the near future, existing storage methods (i.e., magnetic and optical media) will be insufficient to store these exponentially growing data. Therefore, data scientists are continuously looking for better, more stable and space-efficient alternatives to store these huge datasets. Because of its unique biological properties, highly condensed DNA has great potential to become a storage material for the future. Indeed, DNA-based data storage has recently emerged as a promising approach for long-term digital information storage. This review summarizes state-of-the-art methods, including digital-to-DNA coding schemes and the media types used in DNA-based data storage, and provides an overview of recent progress achieved in this field and its exciting future.

Keywords: DNA digital storage; Binary-DNA encoding scheme; in vivo DNA digital storage; in vitro DNA digital storage

Introduction to DNA-based data storage

The concept of DNA-based data storage was introduced by computer scientists and engineers in the 1960s [1]. In one pioneering attempt, made in 1988 by Joe Davis in his seminal artwork “Microvenus” [2], an icon was converted into a string of binary digits, encoded into a 28 base-pair (bp) synthetic DNA molecule, and was later successfully sequenced to retrieve the icon [2]. Although Microvenus was originally designed for interstellar communications, it demonstrated that non-biological information could also be stored in DNA. Later, in the early 2000s, Bancroft et al. proposed a simple way to use codon triplets for encoding alphabets, suggesting great potential for DNA as a storage medium [3]. Now we ask the question: what makes DNA so inimitable for data storage?

Four unique biological features make DNA the focus of the next generation of digital information storage. Firstly, DNA is remarkably stable compared with other storage media. With its double-helix structure and base-stacking interactions, DNA can persist a thousand times longer than a silicon device [4], and survive for millennia, even in harsh conditions [5–8]. Secondly, DNA possesses a high storage density. Theoretically, each gram of single-stranded DNA can store up to 455 exabytes of data [9]. As storage strategies continue to improve, scientists have now achieved a density that could reach this theoretical limit. Thirdly, DNA can be easily and rapidly replicated through the polymerase chain reaction (PCR), thereby providing the possibility for large-scale data backup. It should not be neglected that living cells are also perfect tools for in vivo information replication and backup. Last but not least, the biological properties of DNA enable current sequencing and chemical synthesis technologies to read and write the information stored in DNA, thereby making it an excellent material to store and retrieve data [9].

The recently announced Lunar Library™ project aims to create a DNA archive of a collection of 10,000 images and 20 books for long-term backup storage on the Moon. This highlights the advantage and immense potential of DNA as a medium for long-term digital data storage.

The accessibility of DNA-based data storage is mainly driven by two empowering techniques: DNA synthesis for ‘encoding’, and DNA sequencing for ‘decoding’[10]. Typically, digital information is first transcoded into ATCG sequences using a predeveloped coding scheme. These sequences are then synthesized into oligonucleotides (oligos) or long DNA fragments to allow long-term storage. To retrieve the data, a DNA sequencing method is applied to obtain the original ATCG sequence from the synthesized DNA.

Overview of current coding schemes for DNA-based data storage

Summarizing the findings of earlier studies, an optimal coding scheme usually outperforms in achieving three main features:

1) High fidelity – during data retrieval, there is a trade-off between accuracy and redundancy. While additional redundancy helps to improve accuracy, it also increases data size. Hence, to strike a balance, appropriate coding scheme and error correction strategies are applied to avoid and rectify errors induced during DNA synthesis or sequencing.
2) High coding efficiency – by having four elementary bases, DNA has the theoretical coding potential to store at least twice as much information in quaternary scaffolds as binary codes.
3) Flexible accessibility – from a computer science standpoint, stored data is expected to have random access. Lack of random access hampers attempts to scale up the data size because it will be impractical to sequence and decode the whole dataset each time when we only want to retrieve a small amount of data.

Correspondingly, proposed coding schemes are usually designed to fulfill all of the above characteristics. Generally, DNA-based data storage coding schemes can be differentiated by their binary transcoding methods (Fig. 1), or by the ways in which they add redundancy to increase fidelity (Fig. 2).

‘Simple’ code coding scheme

In 2012, Church et al. proposed a simple code to tackle errors generated by DNA sequencing and synthesis (e.g. repeated sequences, secondary structure and abnormal GC content) [9]. By employing the free base swap strategy (a ‘one-to-two’ binary transcoding method, Fig.1A), Church and colleagues encoded approximately 0.65 MB data into ~8.8 Mb DNA oligos of 159 nucleotides (nt) in length. Given the large amount of digital data that were successfully

stored in DNA, this was considered to be a milestone study [15], and it also demonstrated the potential of DNA-based data storage to cope with the challenge of the information explosion. However, to allow its base swapping flexibility, this coding scheme sacrifices information density by transcoding each binary code into one base. Later researchers have developed other coding strategies to overcome this issue while maintaining comparable performance.

Huffman coding scheme

Huffman code, developed by David Huffman in the 1950s, is considered to be an optimal prefixed code that is commonly used for lossless data compression. In 2013, Goldman and colleagues adopted the Huffman code in their coding scheme, which effectively improved the coding potential to 1.58 bits/nt [12]. Before transcoding into DNA nucleotides, binary data were first converted into ternary Huffman code, and then transcoded to DNA sequences by referring to a rotating encoding table (Fig. 1B). Each byte of the resulting data was substituted by five or six ternary digits (comprising the digits '0', '1', and '2' only) by Huffman's algorithm [16]. Encoding in this way, as per the rotating table, eliminates the generation of mononucleotide repeats and can compress the original data by 25–37.5%. For ASCII (American Standard Code for Information Interchange) text format files, this type of compression further outperforms by mapping the most common characters to five-digit ternary strings [12]. However, the transcoding algorithm cannot prevent abnormal GC distribution when dealing with certain binary patterns. In addition, this coding scheme employs simple parity check coding to detect errors, and maintains a four-fold coverage redundancy to prevent error and data loss (Fig. 2A). However, while the simple parity check coding can detect errors, it cannot correct them. Moreover, increased redundancy inevitably lowers the coding efficiency. Although not perfect, this work not only improved coding efficiency and prevented nucleotide homopolymers, but also introduced a strategy to ensure fidelity by adding redundancy.

Improved Huffman coding scheme

In 2016, Bornholt et al. improved Goldman's encoding scheme with an exclusive-or (XOR) encoding principle [13], using an XOR (⊕) operation to yield redundancy. As shown in Fig. 2B, every two original sequences, A and B, will generate a redundant sequence C by A⊕B. Therefore, with any two sequences (AB, AC or BC), one can easily recover the third sequence. This coding scheme also provides the flexibility of redundancy according to the level of significance of particular data strands, namely 'tunable redundancy'. It decreased the redundancy of the original data from three-fold to half, providing an efficient way to ensure fidelity. In practice, this coding scheme successfully encodes four files with a total size of 151 KB, and recovers three out of four files without manual intervention [13].

The need to amplify target files in a large-scale database suggests a necessity for random access in DNA-based data storage. Therefore, in 2018, the Bornholt et al. put forward another error-free coding scheme that allowed users to randomly reach and recover individual files in a large-scale system. In this coding scheme, unique PCR primers are assigned to individual files after rigorous screening, thereby allowing users to randomly access their target file(s). A total of 200 MB data was successfully stored and recovered in their study, which set a new milestone by complementing the feasibility of storing large-scale data in DNA [14].

A coding scheme based on Galois Field and Reed–Solomon Code

With special emphasis on error detection and correction, a coding scheme based on the Galois field and Reed–Solomon (RS) code [15] was proposed by Grass and colleagues in 2015 [17], improving potential data density to ~1.78 bits/nt. With the two-byte (8×2 bits) fundamental information block, this coding scheme introduced a finite field (Galois field; GF) of DNA nucleotide triplets as its elements (Fig. 1C). To prevent mononucleotide repeats of greater than 3 nt during encoding, the last two nucleotides of the triplet are varied, which can give 48

different triplets. A GF of 47 was used because 47 is the largest prime number smaller than 48. The information block is then mapped to the three elements in GF (47), i.e., 256^2 to 47^3. The RS code is applied in this scheme to detect and correct errors. As shown in Fig. 2C, two rounds of RS coding are applied horizontally and vertically to the matrix generated by GF transcoding, respectively.

In this pilot study, 83 KB of text data were encoded in silico [17]. Although the data size was not impressive, it underlined the necessity to apply error-correction coding, and significantly enhanced coding efficiency. Moreover, error-correction code from the information communication field was applied to DNA-based data storage for the first time.

A ‘forward error correction’ coding scheme

Blawat and colleagues proposed a coding scheme to particularly tackle the errors generated during DNA sequencing, amplification and synthesis (e.g., insertion, deletion and substitution) [18]. The potential coding density was 1.6 bits/nt. Two reference coding tables are specified in advance. A one-byte (8 bits) fundamental information block is assigned to a 5-nt DNA sequence, and the third and fourth nucleotide are swapped (Fig. 1D). Two other criteria are also applied to prevent mononucleotide repeats during this process: 1) the first three nucleotides should not be the same; and 2) the last two nucleotides should not be the same. Consequently, an 8-bit data block (i.e., $2^8 = 256$ permutations for binary data) is transcoded into 704 different DNA blocks (4^5- 4^3- 4^4) [18]. These can be categorized into three clusters: clusters A and B of complete blocks (256 each), and cluster C of 192 incomplete blocks. Data can then be mapped to DNA blocks A and B as required, e.g., alternately mapped to A or B.

In this study, 22 Mb of data was successfully encoded and stored in an oligo pool. Those data were retrieved without error, thereby proving the feasibility of the ‘forward error correction’ coding scheme. However, this was not the case for detecting and correcting single mutations. For example, ‘11100011’ could be mapped to a DNA block ‘TGTAG’. but if an A-to-T

transversion occurs, the DNA block will be changed to ‘TGTTG’, which will give an error byte ‘11101111’ after decoding.

Fountain code-based DNA-based data storage coding scheme

In 2017, Erilich and Zielinski used fountain code in their coding scheme [19]. Fountain code is a widespread method of coding information in communication systems, and is well known for its robustness and high efficiency [20]. Fountain code is also known as a rateless erasure code, in which data to be stored are divided into k segments, namely resource packets. A potentially limitless number of encoded packets can be derived from these resource packets. When it returns n ($n > k$) encoded packets, the original resource data will be perfectly recovered. In practice, n only needs to be slightly larger than k to yield greater coding efficiency and robustness for information communication [21].

Binary data nucleotide sequence transcoding is also carried out. A fundamental two-bit to one-nucleotide transcoding table is adopted, in which [00, 01, 10, 11] is mapped to [A, C, G, T], respectively (Fig. 1A). Firstly, original binary information is segmented to small blocks. These blocks are chosen according to a pre-designed pseudorandom sequence of numbers. A new data block is then created by the bitwise addition of selected blocks with random seeds attached and transcoded to nucleotide blocks according to the transcoding table. Mononucleotide repeats and abnormal GC content are prevented by a final verification step (Fig. 2D) [19].

The oligos in this coding scheme are correlated and have grid-like topology to realize extremely low but necessary redundancy. This study increased the theoretical limit of coding potential to an unprecedentedly high value of 1.98 bits/nt, and remarkably reduced the desired redundancy for error-free recovery of the source file. Moreover, the mechanism of random selection and validity verification ensures that long single-nucleotide homopolymers do not appear in the encoded sequence. However, in this coding scheme, the complexity level of

encoding and decoding is not linearly correlated to the data size. Thus, decoding can be complicated and may require more resource and a longer computation time. However, although it is claimed that a 4% loss of total packets would not affect the recovery of the original file in the report, in terms of the features of DNA fountain code, loss of more packets may cause complete failure of recovery. If the ultimate aim is to permanently store the data, the amount of redundancy must be increased to ensure information integrity.

If we consider DNA-based data storage solely as an archiving process with high fidelity, then DNA fountain coding appears to be the only communication-based coding scheme. In DNA-based data storage and retrieval, the most common error is caused by a single nucleotide mutation. To address this issue, most coding schemes create high redundancy to tackle the challenging conditions of current communication channels. However, these error correction algorithms require complex decoding procedures and large amounts of computing resources. Here, the use of a fountain coding scheme firstly shows that it is unnecessary to employ error detection/correction algorithms, and this provides us with an alternative solution for improving the performance of DNA coding.

Overview of DNA-based data storage mediums

Currently, DNA-based data storage uses two main types of media to store encoded DNA sequences: in vivo and in vitro.

In vivo DNA-based data storage

In vivo DNA-based data storage was commonly adopted in pioneering DNA-based data storage work, such as the Microvenus project, which used bacteria as the storage medium [2]. In the 2000s, other research teams also proposed simple techniques for in vivo DNA-based data storage, e.g. the use of codon triplets to encode alphabets [22] or bits [23] by either

transferring plasmids or introducing site-directed mutagenesis. Typically, encoded DNA sequences are firstly cloned into a plasmid and then transferred into bacteria. Therefore, the DNA sequences, and the information they carry, can be maintained in tiny bacteria and their billions of descendants.

Nevertheless, the capacity of bacteria for carrying plasmids is limited by the type and size of plasmid. In addition, plasmid mutation is quite common in bacteria. During bacterial replication, take *Escherichia coli* as an example, the spontaneous mutation rate is 2.2×10^{-10} mutations per nucleotide per generation, or 1.0×10^{-3} mutations per genome per generation [24], with a generation time of 20–30 minutes, which – after a few years – might ultimately alter the information stored.

Recently, Church et al. demonstrated a novel method to encode an image and a short movie clip into the bacterial genome using the CRISPR-Cas system with Cas1-Cas2 integrase [25]. Although, reportedly, the CRISPR-Cas system is not equally efficient to all sequences, this work greatly improved the capability of in vivo DNA-based data storage.

In vitro DNA-based data storage

In vitro DNA-based data storage is seen more frequently than the in vivo version in recent studies. The oligo library is one of the most popular forms, primarily because of the maturation of the array-based high-throughput oligo synthesis technique [26], which makes the synthesis of large numbers of DNA oligos more cost-effective.

During the synthesis process, each oligo is assigned a short tag, or index, because all oligos are mixed together for high throughput synthesis and sequencing. The current oligo synthesis technique can generate, at most, 200-mers, with relatively high accuracy and purity [27]. Hence, the index should be as short as possible to save the information capacity in each oligo. Apparently, many more indices will be needed if more DNA oligo sequences are generated

and mixed. However, similar to in vivo DNA-based data storage, the larger data size demands more DNA oligos for in vitro DNA-based data storage. This increases the size of indices in oligo and thus lowers the storage capacity and efficiency.

To overcome these problems, longer DNA fragments can be used instead of DNA oligos. In 2017, Yadzi et al successfully encoded 3,633 bytes of information (two images) into 17 DNA fragments, and recovered the image using homopolymer error correction [28]. Nevertheless, the current cost of DNA fragment synthesis is higher than that of oligo synthesis, which increases the overall cost of DNA fragment-based storage.

Above all, both in vivo and in vitro strategies have been employed in current DNA-based data storage research. However, the nature of these two strategies demonstrates the usage of different techniques and different application scenarios (Table 1). Although in vivo storage is a more complicated procedure than oligo pool synthesis in terms of backup cost, in vivo DNA-based data storage is more cost-effective. The cost of the in vitro method has been reduced with the development of array-based oligo synthesis and high-throughput sequencing. Considering long-term storage, DNA in an in vivo condition will degrade more slowly than in vitro. Nevertheless, errors induced by mutations during replication in vivo are more significant than those induced by synthesis because of the high accuracy of current DNA synthesis technology.

Table 1. Comparison of in vivo and in vitro DNA-based data storage

	**In vivo**	**In vitro**
**Medium**	Plasmid<br>Bacterial genome	Oligo library<br>Long DNA fragment
**Information writing**	Cloning and gene editing	Oligo synthesis

**Main Cause for error generation**	Mutation<br>Sequencing	Error in synthesis/sequencing
**Advantage**	Long-term storage<br>Cost-effective backup	High-throughput<br>Low error rate<br>Easy for manipulation
**Disadvantage**	Limited DNA size<br>Mutation during replication	DNA degradation<br>Cost of index region

Other pioneering work goes beyond the aforementioned DNA-based data storage system. Song and Zeng proposed a strategy that they claim is able to detect and correct errors in each byte [29]. They transformed a short message into *E. coli* stellar competent cells and proved the reliability of their strategy; this was one of the first studies to evaluate the stability of in vivo storage. Lee et al. incorporated enzymatic DNA synthesis and DNA-based data storage principles, reporting an enzymatic DNA-based data storage strategy [30]. Nevertheless, the recent recombinase and CRISPR-Cas9 techniques cannot be neglected, since they might also drive in vivo DNA-based data storage in diversiform. All of this research has laid a sound foundation for the global application of this novel storage medium.

Challenges of DNA-based data storage

Although DNA sequencing and DNA synthesis techniques largely facilitated the increase in DNA-based data storage, challenges co-derived and spontaneously evolve as each paradigm shift occurs in these fields. Fig. 4 shows a timeline briefly summarizing the key breakthroughs in DNA synthesis and sequencing that have transformed the development of DNA-based data storage.

In the pre-high throughput period, column-based oligo synthesis [31] and Sanger sequencing [32, 33] represented the dominant DNA synthesis and DNA sequencing techniques, respectively. At this stage, the high cost ($0.05–0.15 USD per nucleotide in 100-nt synthesis; $1 USD per 600–700 bp per sequencing read) and time-consuming nature of DNA sequencing (an automated Sanger sequencing machine reads 1,000 bases per day) [10, 26] remain the major challenges for DNA-based data storage, preventing its application on larger datasets. Therefore, studies during that time were only conducted as a proof-of-concept on a relatively small scale [2].

From 2000 onwards, on the completion of the Human Genome Project, both DNA synthesis and DNA sequencing techniques were transformed to the high-throughput scale. Array-based oligo synthesis gradually superseded column-based oligo synthesis and was widely commercialized [34, 35, 36], largely because of its relatively low cost ($0.00001–0.001 USD per nucleotide synthesis [10]). However, as oligo length increases – presumably because of potential false cross-hybridization during synthesis – the error rate also increases. Moreover, the length of synthesized oligonucleotides is limited to below 200-mers; this is because the product yield drops as oligos are elongated thanks to limitations in the efficiency of chemical interactions. Although gene size (200–3,000 bp or above) array-based synthesis has been developed [37], these usually require additional steps for error correction, causing the final cost and time consumed to be high. Consequently, for cost-saving purposes and to reduce the complexity of DNA synthesis, the primary storage unit employed in DNA-based data storage is below 200 nt.

The concept of massively parallel sequencing (or next-generation sequencing; NGS), a high-throughput sequencing method, was proposed in 2000 [38]. In the following years, sequencing by ligation and by synthesis became major players in the sequencing field. Multiple NGS platforms became commercially available (e.g. 454, Solexa, Complete Genomics), which paved the way for high-throughput DNA-based data storage. However, this

emerging technique also comes with limitations. Most NGS platforms require in vitro template amplification with primers to generate a complex template library for sequencing. During this process, copying errors, sequence-dependent biases (for example, in high-GC and low-GC regions and at long mononucleotide repeats) and information loss (for example, methylation) are produced [9].

In 2012, Church and colleagues successfully demonstrated the first application of high-throughput DNA synthesis and NGS in DNA-based data storage [9]. It initiated rapid development of coding schemes incorporating NGS. Two of the most common goals at this stage were how to improve coding efficiency, and how to correct sequencing errors.

Table 2. Summary of frequently used sequencing platforms in DNA-based data storage (data retrieved from [40]).

Platform	Error rate (%)	Runtime	Instrument Cost (US$)	Cost per Gb (US$)	Reference
**Illumina MiSeq**	0.10	4–56 hours*	99,000	110–1000*	[12,15,18,25]
**Illumina HiSeq 2000**	0.26†	3–10 days*	654,000	41	[8,11]
**Illumina HiSeq 2500**	0.10	7 hours–6 days†,*	690,000	30–230*	[17]
**Illumina NextSeq**	0.20†	11–29 h*	250,000	33–43*	[13]
**Oxford Nanopore MinION**	8.0†	up to 48 h	1,000	70†	[13,28]

Gb, gigabase pairs; †, latest data retrieved from the industrial report (may be different from previous literature); * varied by read length and reagent kit version Gb, gigabase pairs; †, latest data retrieved from the industrial report (may be different from previous literature); * varied by read length and reagent kit version

While NGS remains dominant, real-time, single-molecule sequencing (or third generation sequencing) is continually evolving [39,41]. Despite its relatively high sequencing error rate (~10%), it is reportedly capable of long read-length sequencing, high-GC tolerant, and generates only random errors [28]. These characteristics mean it outperforms NGS counterparts and make it ideal for data retrieval in DNA-base data storage. In 2017, Yazdi et al. used Oxford Nanopore MinION technology to retrieve data stored in DNA, showing optimal robustness and high efficiency [28]. This study implies a possible shift from NGS to single-molecule sequencing because of its potential for compactness and stand-alone DNA data storage systems [13, 30]. Table 2 summarizes the frequently used sequencing platforms in DNA-based data storage. Recently, Oxford Nanopore Technologies announced plans to develop a 'DNA writing' technique using their nanopore technology. Using the same platform to both read and write, they claim it will be possible to selectively modify native bases and stimulate localized reactions, such as light pulses for encoding, which will provide real-time read and write capabilities for DNA-based data storage [42].

In 2018, Oxford Nanopore also launched a high-throughput sequencing platform, PromethION, stating that it has the potential to yield up to 20 Tb of data in 48 hours [43,44]. The first metagenomics data published using the PromethION demonstrated that it is already possible to obtain 150 Gb of data from two flowcells in a 64-hour run [45]. Further developments and improvements are in progress. Since the performance of this technology is getting closer to that of its NGS counterparts, it may play a more prominent role in the future study of DNA-based data storage.

Perspectives on DNA-based data storage

Taken together, DNA-based data storage techniques provide us with the great possibility to manipulate DNA as a carbon-based archive with excellent storage density and stability. Imperfect as it is, it may become the ultimate solution to the current data storage market for long-term archiving. We are also excited to see that multidisciplinary research companies have already joined this revolution to make DNA-based archiving commercially viable.

In terms of coding schemes, although the current theoretical limit of bit-base transcoding is 2 bits/base, newly discovered unnatural nucleic acids could expand the choice of bases for transcoding, and thus increase the theoretical limit. X and Y are two classical unnatural nucleic acids that demonstrated the capability to be integrated into normal cells, and in pairing, replication and amplification [46]. Moreover, recent synthetic biology research reported four new synthetic nucleic acids: Z, P, S and B [47]. These new nucleic acid candidates could help to increase the coding efficiency for DNA digital storage in the not-too-far future.

Enterprises with a strong DNA synthesis background are most commonly seen, given that DNA-based data storage can significantly benefit from the breakthroughs achieved in DNA synthesis. It could be foreseen that with continuously improving enzymatic DNA synthesis techniques, DNA oligo synthesis could break the limit of 200-mers in the near future, providing us with a longer primary storage unit. This will undoubtedly improve net coding efficiency with the same lengths of PCR primers and shorter index sequences. In one model for the DNA-based storage of a 1 GB file under theoretical limitation, one DNA base represented two binary bits. For each DNA oligo, the length of forward and reverse primers was set at 20. In this case, we can deduce the equation representing the relationship between index length i and DNA oligo length l: $log_2(l - 40 - i) + i = 32$ (Equation 1). Hence, we could obtain the correlation between an optimal index length and DNA oligo length.

As Figure 4 shows, as DNA oligo length increases, the index length decreases, while net coding efficiency increases. Some startup companies are now reportedly aiming to develop

industrial enzymatic DNA synthesis technology. If they can successfully synthesize oligos greater than 200-mers, the efficiency of DNA-based data storage will markedly improve.

In addition, the scale of DNA synthesis also affects the information capacity of DNA-based data storage per unit mass. With the development of array-based DNA synthesis technology, high-throughput oligo synthesis is currently directed to the microscale level. In DNA-based data storage, the information capacity of a certain mass of DNA sequences also relates to the copy number of each DNA molecule. The correlation between information capacity C and copy number N_m of each oligo can be calculated from: $C = n \times (N_m \mu \delta \gamma)^{-1}$ (Equation 2), where n represents the number of bytes carried by each oligo (normally 10–20 bytes/molecule according to different coding schemes); μ is the number of nucleotides per molecule, δ is 320 Dalton/nucleotide; and γ is 1.67×10^{-24} g/Dalton. To date, the copy number of oligos is around 10^7 molecules in on-chip high-throughput synthesis (without dilution) [19]. According to Equation 2, this will give an information capacity level of ~10^{13} bytes/g. If the copy number is decreased to 10^4 molecules per oligo, the information capacity will increase to ~10^{16} bytes/g. Additionally, synthesis in microscale will also reduce the cost by several orders of magnitude and save the dilution step.

At present, several DNA synthesis companies are taking the lead in this field, based on their related expertise, and providing services related to DNA-based data storage. Twist Biosciences has reportedly already collaborated with Microsoft in a DNA-based data storage project, providing them with oligo pool services [14] using their high-throughput, array-based DNA synthesis technique. Microsoft, together with the University of Washington, launched the 'Memories in DNA' project, and will collaborate with the Arch Mission Foundation to construct the first Molecular Collection of the aforementioned Lunar Library. Given that these companies are starting to push this business forward, it will be interesting to see how commercial and social applications develop in the future.

Apart from companies with biological backgrounds, information technology (IT)-based industries are also playing an important role in this revolution. As the coding schemes used in DNA-based data storage must yet be improved to yield higher coding efficiency and fidelity, efforts from the IT field could be of critical importance. For example, from random access data retrieval to scaling up data storage [13], Microsoft successfully implemented its IT philosophy in DNA-based data storage and is marching steadily towards its goal announced in 2017: a proto-commercial system in three years to storing some amount of data on DNA [48]. A recent paper written in collaboration with a scientist from the University of Washington described an automated end-to-end DNA-based data storage device, in which 5 bytes of data were automatically processed by the write, store, and read cycle [48]. Further efforts to speed up the coding and decoding process for daily storage applications are still essential.

We expect more entities and research organizations to join this cohort to eventually make carbon-based archiving a reality, and, further, to attain immediate access storage (IAS) or biological computation. Nevertheless, it remains a priority to maintain a safe and ethical framework for the development of DNA-based data storage. Since DNA is the basic building block of genetic information for living organisms, situations might arise in which synthesized sequences are introduced into living host organisms, and this could lead to biological incompatibility caused by unknown toxicity or other growth stresses. Hence, it is necessary to evaluate the safety of sequences prior to their synthesis. We long to see the day when the safety, capacity and reliability of DNA means it will become the next-generation digital information storage medium of choice.

List of abbreviations

bp, base pair; oligos, oligonucleotides; Gb, Giga base pairs; GF, Galois field; KB, Kilobytes; MB, Megabytes; Mb, Megabases; nt, nucleotide; PCR, Polymerase chain reaction; RS, Reed–Solomon.

Competing interests

Z.P., D.Z.M., X.L.H., S.H.C., L.Y.L., F.G., and Y.S. are employees of BGI Shenzhen. The authors otherwise declare that they have no competing interests.

Funding

This work was supported by the Guangdong Provincial Academician Workstation of BGI Synthetic Genomics (No. 2017B090904014), Guangdong Provincial Key Laboratory of Genome Read and Write（No. 2017B030301011), and Shenzhen Engineering Laboratory for Innovative Molecular Diagnostics（DRC-SZ[2016]884).

Author contributions

Z.P., D.Z.M., X.L.H.; Collected materials, reviewed literatures and co-wrote the paper. S.H.C., L.Y.L., F.G.; Supported materials collection and revised the paper. S.J.Z., Y.S.; Supervised this review and co-wrote the paper. All authors read and approved the final manuscript.

Acknowledgements

Not applicable.

References

1. Neiman MS. Some fundamental issues of microminiaturization. *Radiotekhnika*. 1964;No. 1:3-12.

2. Joe Davis a. Microvenus. Art Journal. 1996; 1:70. doi:10.2307/777811.

3. Bancroft C, Bowler T, Bloom B and Clelland CT. Long-term storage of information in DNA. Science. 2001;293 5536:1763-5.

4. Bonnet J, Colotte M, Coudy D, Couallier V, Portier J, Morin B, *et a*l. Chain and conformation stability of solid-state DNA: implications for room temperature storage. *Nucleic Acids Research*. 2010;38 5:1531-46. doi:10.1093/nar/gkp1060.

5. Pääbo S, Poinar H, Serre D, Jaenicke-Després V, Hebler J, Rohland N, *et al*. GENETIC ANALYSES FROM ANCIENT DNA. *Annual Review of Genetics*. 2004;38:645-79. doi:10.1146/annurev.genet.37.110801.143214.

6. Kool ET. Hydrogen bonding, base stacking, and steric effects in DNA replication. Annual Review of Biophysics & Biomolecular Structure. 2001;30 1:1-22.

7. Nelson DL, Cox MM and Lehninger AL. *Lehninger principles of biochemistry*. New York ; Basingstoke : W.H. Freeman, c2008. 5th ed.; 2008

8. Pierce BA. Genetics : a conceptual approach. New York, NY : W.H. Freeman, c2012. 4th ed., International ed.; 2012.

9. Church GM, Gao Y and Kosuri S. Next-generation digital information storage in DNA. *Science*. 2012;337 6102:1628. doi:10.1126/science.1226355.

10. Shendure J, Balasubramanian S, Church GM, Gilbert W, Rogers J, Schloss JA, et al. DNA sequencing at 40: past, present and future. *Nature*. 2017;550 7676:345-53. doi:10.1038/nature24286.

11. De Silva PY and Ganegoda GU. New Trends of Digital Data Storage in DNA. *Biomed Res Int*. 2016;2016:8072463. doi:10.1155/2016/8072463.

12. Goldman N, Bertone P, Chen S, Dessimoz C, LeProust EM, Sipos B, et al. Towards practical, high-capacity, low-maintenance information storage in synthesized DNA. *Nature*. 2013;494 7435:77-80. doi:10.1038/nature11875.

13. Bornholt J, Lopez R, Carmean DM, Ceze L, Seelig G and Strauss K. A DNA-Based Archival Storage System. *SIGPLAN Not*. 2016;51 4:637-49. doi:10.1145/2954679.2872397.

14. Organick L, Ang SD, Chen YJ, Lopez R, Yekhanin S, Makarychev K, et al. Random access in large-scale DNA data storage. *Nat Biotechnol*. 2018;36 3:242-8. doi:10.1038/nbt.4079.

15. Reed I and Solomon G. Polynomial Codes Over Certain Finite Fields. *Journal of the Society for Industrial and Applied Mathematics*. 1960;8 2:300-4. doi:10.1137/0108018.

16. Huffman DA. A Method for the Construction of Minimum-Redundancy Codes. *Proceedings of the IRE*. 1952;40 9:1098-101. doi:10.1109/JRPROC.1952.273898.

17. Grass RN, Heckel R, Puddu M, Paunescu D and Stark WJ. Robust chemical preservation of digital information on DNA in silica with error-correcting codes. *Angew Chem Int Ed Engl*. 2015;54 8:2552-5. doi:10.1002/anie.201411378.

18. Blawat M, Gaedke K, Hütter I, Chen X-M, Turczyk B, Inverso S, et al. Forward Error Correction for DNA Data Storage. *Procedia Computer Science*. 2016;80:1011-22. doi:10.1016/j.procs.2016.05.398.

19. Erlich Y and Zielinski D. DNA Fountain enables a robust and efficient storage architecture. *Science*. 2017; 6328:950. doi:10.1126/science.aaj2038.

20. Byers JW, Luby M, Mitzenmacher M and Rege A. A digital fountain approach to reliable distribution of bulk data. *Proceedings of the ACM SIGCOMM '98 conference on Applications, technologies, architectures, and protocols for computer communication*. Vancouver, British Columbia, Canada: ACM, 1998, p. 56-67.

21. MacKay DJ. Fountain codes. *IEE Proceedings-Communications*. 2005;152 6:1062-8.

22. Wong PC, Wong KK and Foote H. Organic data memory using the DNA approach. *Commun Acm*. 2003;46 1:95-8. doi:Doi 10.1145/602421.602426.

23. Arita M and Ohashi Y. Secret signatures inside genomic DNA. *Biotechnol Prog*. 2004;20 5:1605-7. doi:10.1021/bp049917i.

24. Lee H, Popodi E, Tang HX and Foster PL. Rate and molecular spectrum of spontaneous mutations in the bacterium Escherichia coli as determined by whole-genome sequencing. *P Natl Acad Sci USA*. 2012;109 41:E2774-E83. doi:10.1073/pnas.1210309109.

25. Shipman SL, Nivala J, Macklis JD and Church GM. CRISPR-Cas encoding of a digital movie into the genomes of a population of living bacteria. *Nature*. 2017;547 7663:345-9. doi:10.1038/nature23017.

26. Kosuri S and Church GM. Large-scale de novo DNA synthesis: technologies and applications. *Nature methods*. 2014;11 5:499-507. doi:10.1038/nmeth.2918.

27. Ma S, Tang N and Tian J. DNA synthesis, assembly and applications in synthetic biology. *Curr Opin Chem Biol*. 2012;16 3-4:260-7. doi:10.1016/j.cbpa.2012.05.001.

28. Yazdi S, Gabrys R and Milenkovic O. Portable and Error-Free DNA-Based Data Storage. *Sci Rep*. 2017;7 1:5011. doi:10.1038/s41598-017-05188-1.

29. Song L and Zeng AP. Orthogonal Information Encoding in Living Cells with High Error-Tolerance, Safety, and Fidelity. *ACS Synth Biol*. 2018;7 3:866-74. doi:10.1021/acssynbio.7b00382.

30. Lee HH, Kalhor R, Goela N, Bolot J and Church GM. Enzymatic DNA synthesis for digital information storage. *bioRxiv*. 2018; doi:10.1101/348987.

31. Beaucage SL and Caruthers MH. Deoxynucleoside phosphoramidites—A new class of key intermediates for deoxypolynucleotide synthesis. *Tetrahedron Letters.* 1981;22 20:1859-62. doi:https://doi.org/10.1016/S0040-4039(01)90461-7.

32. Sanger F, Nicklen S and Coulson AR. DNA sequencing with chain-terminating inhibitors. *Proc Natl Acad Sci USA*. 1977;74 12:5463-7. doi:10.1073/pnas.74.12.5463.

33. Maxam AM and Gilbert W. A new method for sequencing DNA. *Proc Natl Acad Sci U S A*. 1977;74 2:560-4. doi:10.1073/pnas.74.2.560.

34. Hughes TR, Mao M, Jones AR, Burchard J, Marton MJ, Shannon KW, et al. Expression profiling using microarrays fabricated by an ink-jet oligonucleotide synthesizer. *Nat Biotechnol.* 2001;19 4:342-7. doi:10.1038/86730.

35. Singh-Gasson S, Green RD, Yue Y, Nelson C, Blattner F, Sussman MR, et al. Maskless fabrication of light-directed oligonucleotide microarrays using a digital micromirror array. *Nat Biotechnol.* 1999;17 10:974-8. doi:10.1038/13664.

36. Gao X, LeProust E, Zhang H, Srivannavit O, Gulari E, Yu P, et al. A flexible light-directed DNA chip synthesis gated by deprotection using solution photogenerated acids. *Nucleic Acids Res.* 2001;29 22:4744-50. doi:10.1093/nar/29.22.4744.

37. Tian J, Gong H, Sheng N, Zhou X, Gulari E, Gao X, et al. Accurate multiplex gene synthesis from programmable DNA microchips. *Nature.* 2004;432 7020:1050-4. doi:10.1038/nature03151.

38. Brenner S, Johnson M, Bridgham J, Golda G, Lloyd DH, Johnson D, et al. Gene expression analysis by massively parallel signature sequencing (MPSS) on microbead arrays. *Nat Biotechnol.* 2000;18 6:630-4. doi:10.1038/76469.

39. Levene MJ, Korlach J, Turner SW, Foquet M, Craighead HG and Webb WW. Zero-Mode Waveguides for Single-Molecule Analysis at High Concentrations. *Science.* 2003;299 5607:682-6. doi:10.1126/science.1079700.

40. Goodwin S, McPherson JD and McCombie WR. Coming of age: ten years of next-generation sequencing technologies. *Nat Rev Genet*. 2016;17 6:333-51. doi:10.1038/nrg.2016.49.

41. Church G, Deamer DW, Branton D, Baldarelli R and Kasianowicz J. Characterization of individual polymer molecules based on monomer-interface interactions. Google Patents. U.S. Patents 5,795,782. 1998.

42. Oxford Nanopore Previews Upcoming Products, Outlines Nanopore-Based DNA Data Storage Tech. GenomeWeb 2019 https://www.genomeweb.com/sequencing/oxford-nanopore-previews-upcoming-products-outlines-nanopore-based-dna-data-storage-tech#.XOt_1qZS_EY

43. De Coster W, De Roeck A, De Pooter T, D'Hert S, De Rijk P, Strazisar M, et al. Structural variants identified by Oxford Nanopore PromethION sequencing of the human genome. bioRxiv. 2018; doi:10.1101/434118.

44. PromethION. https://nanoporetech.com/products/promethion.

45. Nicholls SM, Quick JC, Tang S and Loman NJ. Ultra-deep, long-read nanopore sequencing of mock microbial community standards. *Gigascience*. 2019;8 5 doi:10.1093/gigascience/giz043.

46. Malyshev DA, Dhami K, Lavergne T, Chen T, Dai N, Foster JM, et al. A semi-synthetic organism with an expanded genetic alphabet. *Nature*. 2014;509 7500:385-8. doi:10.1038/nature13314.

47. Hoshika S, Leal NA, Kim MJ, Kim MS, Karalkar NB, Kim HJ, et al. Hachimoji DNA and RNA: A genetic system with eight building blocks. *Science*. 2019;363 6429:884-7. doi:10.1126/science.aat0971.

48. Antonio Regalado. Microsoft Has a Plan to Add DNA Data Storage to Its Cloud, MIT Technology Review, 2017.

49. Takahashi CN, Nguyen BH, Strauss K and Ceze LH. Demonstration of End-to-End Automation of DNA Data Storage. bioRxiv. 2018; doi:10.1101/439521.

Figure legends

Figure 1 Binary transcoding methods used in DNA-based data storage schemes.

A) One binary bit is mapped to two optional bases [9]. Two binary bits are mapped to one fixed base [11]. B) Eight binary bits are transcoded through Huffman coding and then transcoded to 5 or 6 bases [12]. C) Two bytes (16 binary bits) are mapped to 9 bases [13]. D) Eight binary bits are mapped to 5 bases [14].

Figure 2. Redundancy types used in DNA-based data storage schemes.

A) Increasing redundancy by repetition. B) Increasing redundancy by an exclusive-or (XOR) calculation. C) Increasing redundancy using Reed-Solomon code for two rounds. D) Increasing redundancy using fountain code.

Figure 3. Two categories of DNA-based data storage application.

A) and B) demonstrate two methods of in vitro DNA-based data storage; C) and D) demonstrate two methods of in vivo DNA-based data storage. A) Array-based high-throughput DNA oligo analysis. DNA oligos carrying digital information are stored in the form of oligo pool. B) DNA fragments synthesized by polymerase cycling assembly (PCA) will carry the information to be stored. C) Digital information inserted into a plasmid; plasmids are then transferred into bacterial cells. D) DNA fragments carrying digital information are inserted into the bacterial genome using the CRISPR system using Cas1-Cas2 integrase.

Figure 4. Key events in DNA synthesis and DNA sequencing, and their key applications in DNA-based data storage.

Figure 5. Interrelationship between DNA oligo length, optimal index length and net coding efficiency in a model of 1-GB digital file transcoding.

 Click here to access/download;Table;Table-1.tif

	In vivo	*In vitro*
**Medium**	Plasmid<br>Bacterial genome	Oligo library<br>Long DNA fragment
**Information writing**	Cloning and gene editing	Oligo synthesis
**Main Cause for error generation**	Mutation<br>Sequencing	Error in synthesis/sequencing
**Advantage**	Long-term storage<br>Cost-effective backup	High-throughput<br>Low error rate<br>Easy for manipulation
**Disadvantage**	Limited DNA size<br>Mutation during replication	DNA degradation<br>Cost of index region

Click here to access/download;Table;Table-2.tif

Platform	Error Rate	Runtime	Instrument Cost(US$)	Cost per Gb (US$)	Reference
Illumina MiSeq	0.10%	4-56h*	$99K	$110-1000*	[12]Bornhol et al.,2016<br>[15]Grass et al.,2015<br>[18]Erlich Y and Zielinski D,2017<br>[25]Shipman et al.,2017
Illumina HiSeq 2000	0.26%†	3-10d *	$654K	$41	[8]Church et al.,2012<br>[11]Goldman et al.,2013
Illumina HiSeq 2500	0.10%	7h-6d †,*	$690K	$30-230*	[17]Blawat et al.,2016
Illumina NextSeq	0.20%†	11-29h*	$250K	$33-43*	[13]Organick et al.,2018
Oxford Nanopore MinION	8.0%†	up to 48h	$1K	$70†	[13]Organick et al.,2018<br>[28]Yazdi et al.,2017

d, days; Gb, gigabase pairs; h, hours; K, thousand; † Latest data retrieved from the industrial report, might be different from previous literature; * varied by read length and version of the reagent kit

Figure 1

Click here to access/download;Figure;Figure 1.tif

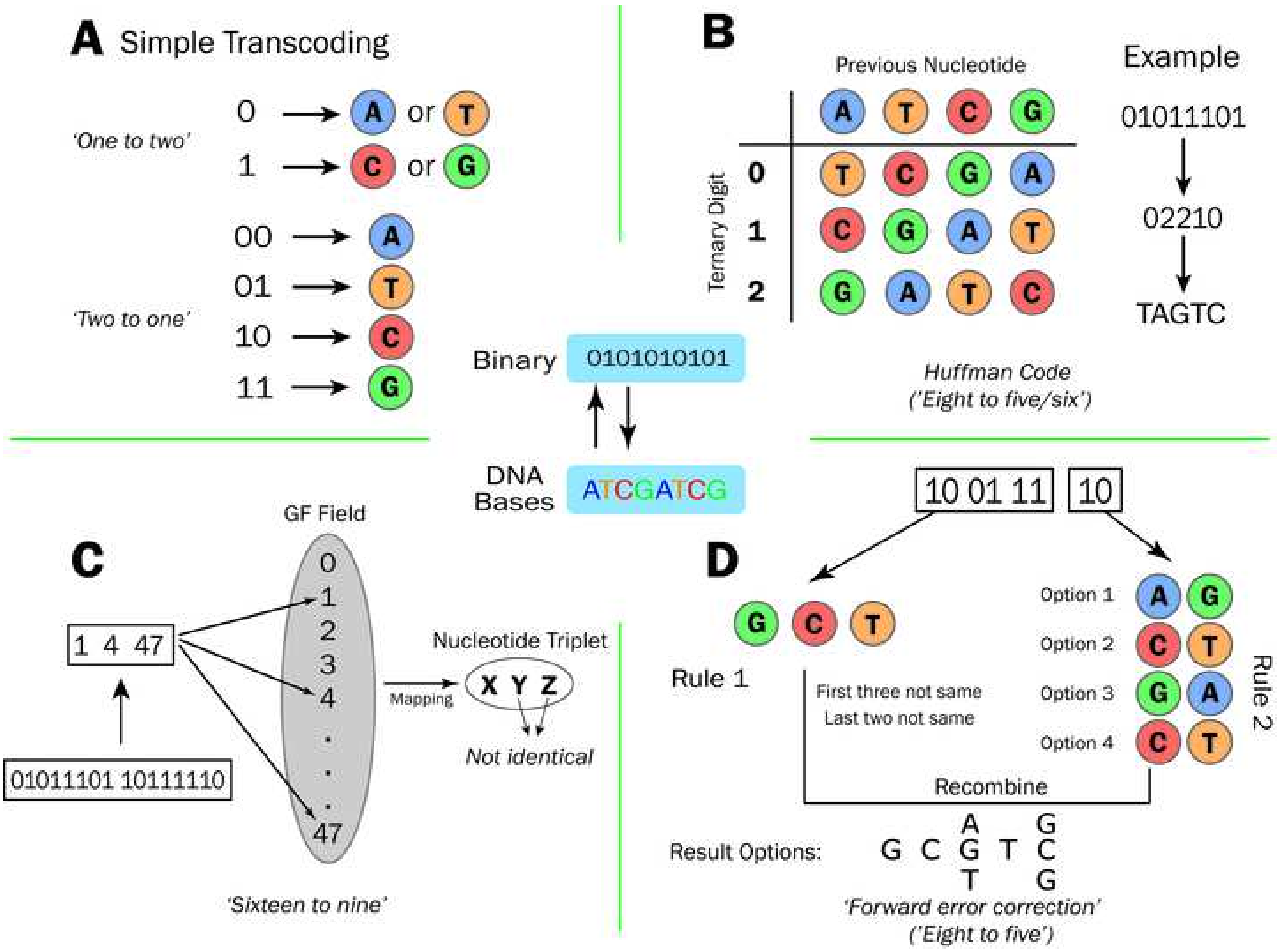

Figure 2

Click here to access/download;Figure;Figure 2.tif

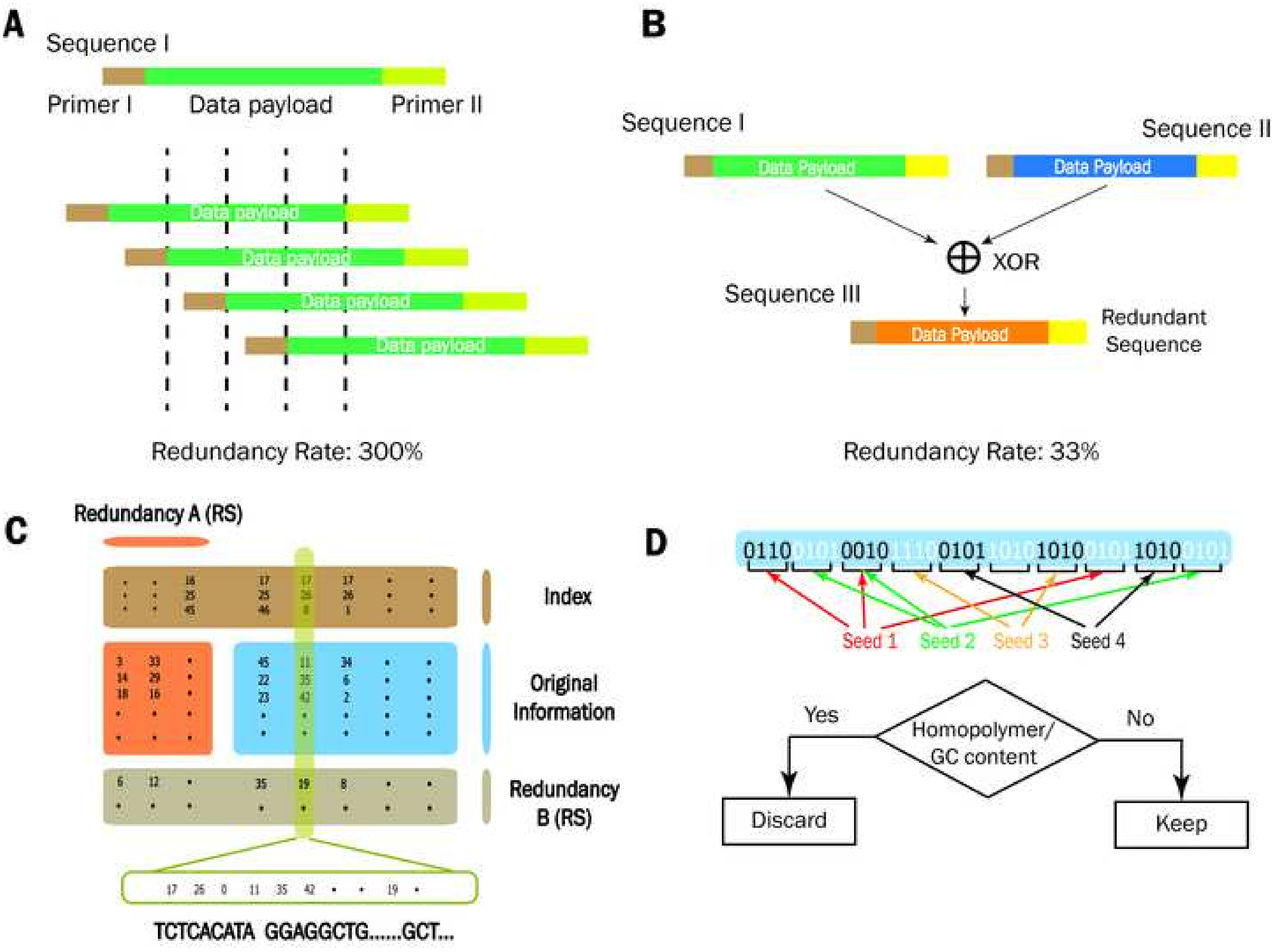

Figure 3

Click here to access/download;Figure;Figure 3.tiff

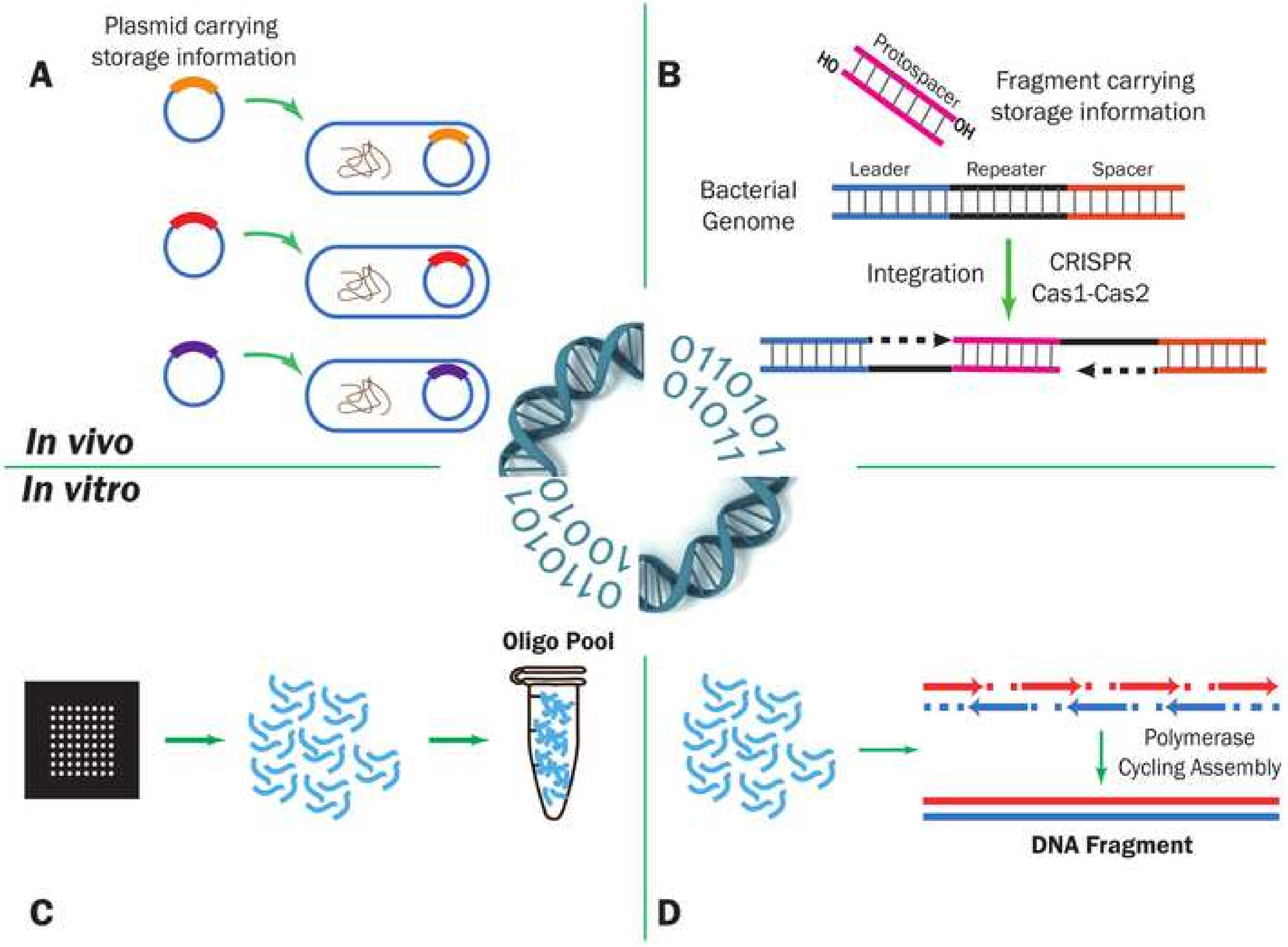

Figure 4

Click here to access/download;Figure;Figure 4.tif

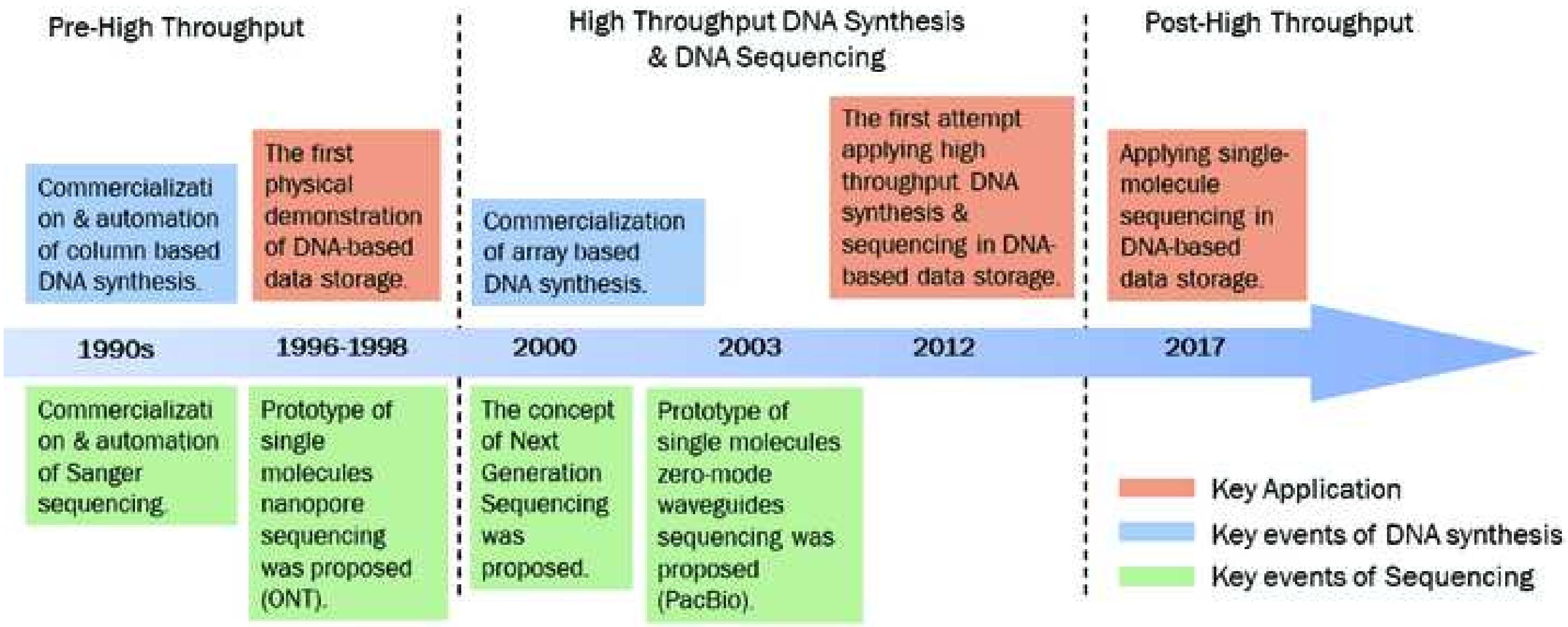

Figure 5

Click here to access/download;Figure;Figure 5.tif

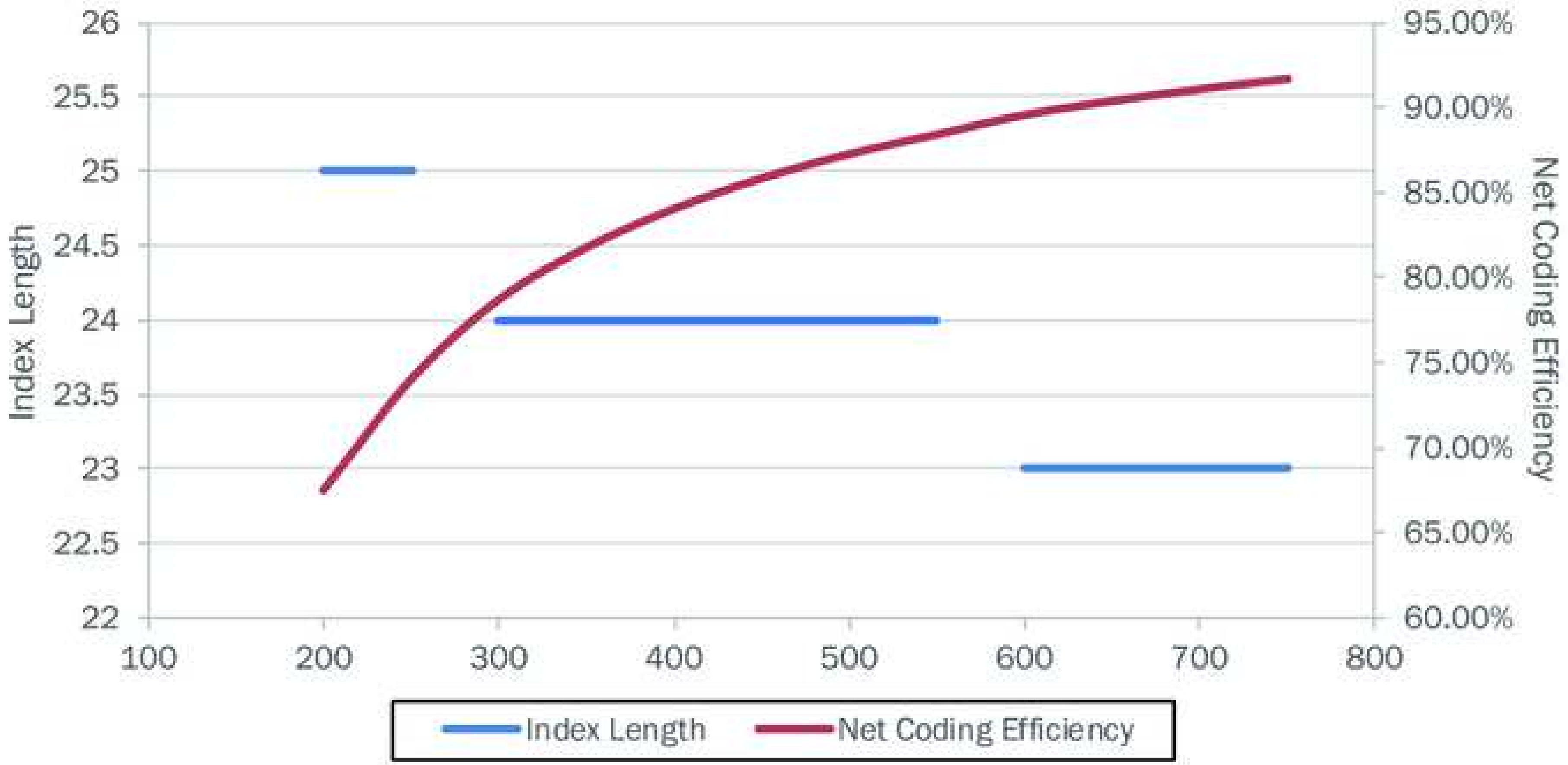

Reply letter to Reviewer #1

Click here to access/download
Supplementary Material
Reply to Refree1_20190521.docx

Reply letter to Reviewer #2

Click here to access/download
Supplementary Material
Reply to Refree2_20190521.docx

Cover letter

Click here to access/download;Personal Cover;Cover letter (Carbon-based Archiving).docx

Dear Editor of GigaScience:

We submit our manuscript entitled “Carbon-based archiving: the current progress and future prospects of DNA-based data storage" to GigaScience for publication.

This manuscript is a review of DNA-based storage with focus on current progress summary on coding scheme and media type. We provide scalable measurements and technical opinions of this field, which we believe will be a great add on to people’s current understanding and help promote its better development. As DNA-based storage is a promising bio-approach for large scale and long term digital information storage, we consider it is well in scope of the GigaScience’s publication criteria.

All authors have read and have abided by the publication ethics as set out by the Commission on Publication Ethics (COPE) for manuscripts submitted to GigaScience.

All authors declared that they have no conflicts of interest to this work.

The work described has not been submitted elsewhere for publication, in whole or in part, and all the authors listed have approved the manuscript that is enclosed.

Thank you very much for your attention and consideration.

Yours sincerely,

Yue (Chantal) Shen

Sha Joe Zhu
