# Supplementary material for: Carbon-based archiving: current progress and future prospects of DNA-based data storage
Source: Gigascience. 2019 Jun 20;8(6):giz075. doi: 10.1093/gigascience/giz075 (PMC6586197; doi:10.1093/gigascience/giz075)
Supplement: giz075_Supplemental_Files [file giz075_supplemental_files.zip › Reply to Refree1_20190521.docx]

Dear Reviewer #1,

Thank you for sparing your valuable time and providing useful suggestions. We have read your comments and made changes to the manuscript accordingly. We have carefully revised the manuscript, and we have also reconstructed some of the sections and added some new information as per your suggestions. Our specific responses are as follows:

1. [Page 3, Lines 9-22]: A 4th unique feature of DNA that might be included is the ease and rapidity with which DNA can be replicated using, for example, PCR.

**Response:** We thank you for your valuable suggestion. As the PCR technique is now well-developed and cost-effective, the replication of DNA sequences encoding digital files are easy and efficient. Meanwhile, for *in vivo* DNA storage, living cells could also replicate rapidly as long as it is active and has sufficient food supply. Therefore, the convenience of file replication and a backup would be another unique feature for DNA-based data storage. The description of this feature is mentioned in [Page 3, Lines18-21].

1. [Page 4: Line 8]: The authors write "there is a trade-off between accuracy and redundancy". In my interpretation, this is counter-intuitive, as additional redundancy should reduce errors.

**Response:** We thank you for your useful suggestion. The additional redundancy, including error-correction codes, are designed to ensure the fidelity of DNA-based data storage. However, the redundancy will use resources (i.e. bases in DNA sequence) and thus reduce the coding density. That is why we mentioned about “the trade-off between accuracy and redundancy”. To clarify this opinion, we have elaborated the details in [Page 4, Lines 10-14].

1. [Page 4: Lines 13-15]: Concerning random access, many experimental works demonstrating DNA data storage do not have random access. Thus it may not necessarily be a requirement. Can the authors discuss this further?

**Response:** We thank you for your valuable suggestion. It is true that many experimental works did not consider random access in DNA-based data storage. However, in the large-scale orthodox storage system (e.g. computer system), random access is one of the most basic features for data retrieval. As a result, the research team from the University of Washington and Microsoft reported the significance of their work on random access for DNA-based data storage. We emphasize the importance of random access in [Page 4, Lines 17-19].

1. [Page 12: lines 5-6]: The amount of time is less informative than citing a number of bacterial divisions/replications over which the data is expected to mutate significantly.

**Response:** We thank you for your useful suggestion. The spontaneous mutation rate in bacterial replication is extremely low depends on the form of storage. The related statements are added in [Page 12, Lines 11-15].

1. [Pages 11-12]: Concerning in-vivo storage, the authors fail to cite a number of early works in DNA data storage that included an in-vivo storage component. For instance: Bancroft 2001, Wong 2003, and Arita 2004.

**Response:** We thank you for the nice suggestion. The early reference of *in vivo* DNA-based data storage has been cited accordingly. These works are mentioned in [Page 3, Lines 8-9 and Page 12, Lines 3-5].

1. [Pages 11-12]: The authors might also want to mention other methods of storing data in vivo, for instance with recombinases, and other molecular recorders like Cas9.

**Response:** We thank you for the useful suggestion. In some recent works, molecular tools like CRISPR-Cas has been described for writing information *in vivo*. The corresponding work is mentioned in [Page 12, Lines 15-18]. Similarly, the possible application of CRISPR and recombinase in DNA-based data storage is mentioned in [Page 14, Lines 8-10].

1. [Page 13, lines 23-25]: Is length really the major challenge? Why not just write-throughput in general, which can be increased by synthesis of longer strands (as stated), and/or by writing more strands in parallel (which is not mentioned) for instance by making larger, more dense oligo synthesis arrays.

**Response:** We thank you for your valuable suggestion. We consider oligo length as one of the major challenges because in DNA-based data storage, in order to retrieve the data, we need indices (e.g. 1,2,3…) to record the address of oligo in a pool of oligo mixture. With the increase in file size , more oligo will be needed and thus larger indices. Therefore, the index region in a data-encoded DNA sequence would be longer and reduce the coding efficiency. With longer oligo length, the number of oligos required to store a file with the same size will be reduced and thus the length of the index region. The explanation of the significance of oligo length is further explained in [Page 13, Lines 2-6].

1. [Page 14, lines 16-20]: This paragraph is confusing, and should be re-written for clarity.

**Response:** We thank you for highlighting this. This whole section is now re-written accordingly in [Page 14 – Page 17].

1. [Table 1]: Costs for HiSeq2500 and NextSeq are missing "K" symbols.

**Response:** We thank you for the useful suggestions. The symbols are now added accordingly.

Thank you again for the peer reviewing.

Best wishes,

Yue (Chantal) SHEN, Ph.D.

Genome Synthesis and Editing Platform, China National GeneBank

BGI-Research

Mobile: +86 150 1383 3483

Address: China National GeneBank (CNGB), Jinsha Road, Dapeng District, Shenzhen, Guangdong, China

Mail: shenyue@genomics.cn
