# Supplementary material for: Carbon-based archiving: current progress and future prospects of DNA-based data storage
Source: Gigascience. 2019 Jun 20;8(6):giz075. doi: 10.1093/gigascience/giz075 (PMC6586197; doi:10.1093/gigascience/giz075)
Supplement: giz075_Response_to_Reviewer_Comments_Original_Submission [file giz075_response_to_reviewer_comments_original_submission.pdf]

Author's Response To Reviewer Comments

Close

Dear Dr. Edmund,

Thank you for the precious advises and we did corrections and revisions on the whole manuscript accordingly.

For the information from twitter you provided in your comments, we tried our best to find some official announcements of this 22 Gb per flow cell, but we could not found it. So we decided not to provide this information in our manuscript.

Thank you again for you kind suggestions.

Best,

Zhi PING, Ph.D
Institute of Genome Synthesis and Editing, China National GeneBank
BGI-Research
Address: China National GeneBank (CNGB), Jinsha Road, Dapeng District, Shenzhen, Guangdong, China

Close
