# Supplementary material for: Carbon-based archiving: current progress and future prospects of DNA-based data storage
Source: Gigascience. 2019 Jun 20;8(6):giz075. doi: 10.1093/gigascience/giz075 (PMC6586197; doi:10.1093/gigascience/giz075)
Supplement: giz075_Response_to_Reviewer_Comments_Revision_1 [file giz075_response_to_reviewer_comments_revision_1.pdf]

Author's Response To Reviewer Comments

Close

Response to Reviewer #1:

Dear Reviewer #1,
Thank you for sparing your valuable time and providing useful suggestions. We have read your comments and made changes to the manuscript accordingly. We have carefully revised the manuscript, and we have also reconstructed some of the sections and added some new information as per your suggestions. Our specific responses are as follows:
1) [Page 3, Lines 9-22]: A 4th unique feature of DNA that might be included is the ease and rapidity with which DNA can be replicated using, for example, PCR.
Response: We thank you for your valuable suggestion. As the PCR technique is now well-developed and cost-effective, the replication of DNA sequences encoding digital files are easy and efficient. Meanwhile, for in vivo DNA storage, living cells could also replicate rapidly as long as it is active and has sufficient food supply. Therefore, the convenience of file replication and a backup would be another unique feature for DNA-based data storage. The description of this feature is mentioned in [Page 3, Lines18-21].

2) [Page 4: Line 8]: The authors write "there is a trade-off between accuracy and redundancy". In my interpretation, this is counter-intuitive, as additional redundancy should reduce errors.
Response: We thank you for your useful suggestion. The additional redundancy, including error-correction codes, are designed to ensure the fidelity of DNA-based data storage. However, the redundancy will use resources (i.e. bases in DNA sequence) and thus reduce the coding density. That is why we mentioned about "the trade-off between accuracy and redundancy". To clarify this opinion, we have elaborated the details in [Page 4, Lines 10-14].

3) [Page 4: Lines 13-15]: Concerning random access, many experimental works demonstrating DNA data storage do not have random access. Thus it may not necessarily be a requirement. Can the authors discuss this further?
Response: We thank you for your valuable suggestion. It is true that many experimental works did not consider random access in DNA-based data storage. However, in the large-scale orthodox storage system (e.g. computer system), random access is one of the most basic features for data retrieval. As a result, the research team from the University of Washington and Microsoft reported the significance of their work on random access for DNA-based data storage. We emphasize the importance of random access in [Page 4, Lines 17-19].

4) [Page 12: lines 5-6]: The amount of time is less informative than citing a number of bacterial divisions/replications over which the data is expected to mutate significantly.
Response: We thank you for your useful suggestion. The spontaneous mutation rate in bacterial replication is extremely low depends on the form of storage. The related statements are added in [Page 12, Lines 11-15].

5) [Pages 11-12]: Concerning in-vivo storage, the authors fail to cite a number of early works in DNA data storage that included an in-vivo storage component. For instance: Bancroft 2001, Wong 2003, and Arita 2004.
Response: We thank you for the nice suggestion. The early reference of in vivo DNA-based data storage has been cited accordingly. These works are mentioned in [Page 3, Lines 8-9 and Page 12, Lines 3-5].

6) [Pages 11-12]: The authors might also want to mention other methods of storing data in vivo, for instance with recombinases, and other molecular recorders like Cas9.
Response: We thank you for the useful suggestion. In some recent works, molecular tools like CRISPR-Cas has been described for writing information in vivo. The corresponding work is mentioned in [Page 12, Lines 15-18]. Similarly, the possible application of CRISPR and recombinase in DNA-based data storage is mentioned in [Page 14, Lines 8-10].

7) [Page 13, lines 23-25]: Is length really the major challenge? Why not just write-throughput in

general, which can be increased by synthesis of longer strands (as stated), and/or by writing more strands in parallel (which is not mentioned) for instance by making larger, more dense oligo synthesis arrays.
Response: We thank you for your valuable suggestion. We consider oligo length as one of the major challenges because in DNA-based data storage, in order to retrieve the data, we need indices (e.g. 1,2,3…) to record the address of oligo in a pool of oligo mixture. With the increase in file size , more oligo will be needed and thus larger indices. Therefore, the index region in a data-encoded DNA sequence would be longer and reduce the coding efficiency. With longer oligo length, the number of oligos required to store a file with the same size will be reduced and thus the length of the index region. The explanation of the significance of oligo length is further explained in [Page 13, Lines 2-6].

8) [Page 14, lines 16-20]: This paragraph is confusing, and should be re-written for clarity.
Response: We thank you for highlighting this. This whole section is now re-written accordingly in [Page 14 – Page 17].

9) [Table 1]: Costs for HiSeq2500 and NextSeq are missing "K" symbols.
Response: We thank you for the useful suggestions. The symbols are now added accordingly.

Thank you again for the peer reviewing.

Best wishes,
Yue (Chantal) SHEN, Ph.D.
Genome Synthesis and Editing Platform, China National GeneBank
BGI-Research
Mobile: +86 150 1383 3483
Address: China National GeneBank (CNGB), Jinsha Road, Dapeng District, Shenzhen, Guangdong, China
Mail: shenyue@genomics.cn

Reply to Reviewer #2

Dear Reviewer #2,
Thank you for sparing your valuable time and providing useful suggestions. We have read your comments and made changes to the manuscript accordingly. We have carefully revised the manuscript, and we have also reconstructed some of the sections and added some new information as per your suggestions.
The objective of this manuscript is to help the readers to understand that coding scheme and storage medium are the two major research focus in DNA-based data storage field. Moreover, we would like to also introduce the challenges in current DNA-based data storage, which may inspire the related researchers to have some ideas for further studies. Therefore, for coding schemes, we tried to introduce some key yet well-accepted bit-to-base algorithms and stated their improvement with respect to coding density, the capability of error correction, and capability of random access. Since there are currently no systematic studies on storage media, we introduced some representative works which employed in vivo and in vitro strategy, and showed their comparative description.
Our specific responses are as follows:
1) Page 3, Advantage of using DNA for storage would also include: easy amplification in vivo by live cells at very low cost, and possible amplification in vitro by enzymatic reaction, e.g., PCR or linear amplification in silico. Both approaches can be used to scale up the backup copy production. One should also consider the possible employment of repair system for correcting errors.
Response: We thank you for your precious suggestion. As the PCR technique is now well-developed and cost-effective, the replication of DNA sequences encoding digital files are easy and efficient. Meanwhile, for in vivo DNA storage, living cells could also replicate rapidly as long as it is active and has sufficient food supply. Therefore, the convenience of file replication and a backup would be another unique feature for DNA-based data storage. The description of this feature is mentioned in [Page 3, Lines 18-21].

2) Page 5-10, the description of the coding schemes is quite sketchy. The outline for each approach was brief and was not well illustrated by the panels in the figure 1 and 2. Better schematics may help, without the need to go back to the original papers to make detailed comparison.
Response: We thank you for your valuable suggestion. In this section, we presented the differences between coding schemes by their different bit-to-base transcoding method for optimize coding efficiency and strategies to add redundancy to ensure fidelity. Although we chronologically presented various coding schemes, we tried to deliver the information that all the coding schemes made improvement based on these two ways. In order to further emphasize it, we have stated this opinion just before the

description of coding schemes. Furthermore, some more details are given for each coding scheme, as well as the description of our current understanding and perspectives. We reconstructed the figure.1 and corrected the mislabeled figure caption in [Page 5] for better presentation.

3) Page 10-12, in vivo and in vitro storage of the information - a thorough comparison of the pros and cons would be helpful, instead of factually describing what methodology is available. The error generated in vivo by mutation should be contrasted to the error in DNA synthesis technology to evaluate the limitation of these tools.
Response: We thank you for this nice suggestion. The in vivo and vitro storage are two strategies that can be distinguished in many aspects. We compared the pros and cons, and added one additional paragraph to discuss the comparison using a table. This paragraph is in [Page 13, Lines 11-20].

4) Page 13, line 4-10. A very typical way of this review in describing methodology citing the previous reports without describing the details and contrasting the differences sufficiently. It does not serve the purpose of a proper analysis of how each method advances the development of storage.
Response: We thank you for your useful suggestion. Since the concept of DNA digital storage was put forward in 2012, many strategies has been reported. In this review, we mainly reviewed the major event of this field in coding scheme and storage medium aspect, instead of molecular tools, synthesis techniques, etc. Some other strategies, including Song et al., and Lee et al., did not made much improvement in coding efficiency or application demonstration. But they also gave some inspirations for this field of research which should not be neglected in our point of view. Therefore, we mentioned these studies briefly for readers who are interested in this field. In addition, we provided brief opinions instead of mere describing the methodology in this part. The amended paragraph is in [Page 14, Lines 2-10].

5) Page 14, sequencing accuracy issue was discussed concerning the data retrieval process. While table 1 summarizes the factual information of the technology available, no clear evaluation of the future direction is given, same as pointed out in (4) above.
Response: We thank you for your precious suggestion. High-throughput sequencing is one of the most significant tools for DNA-based data storage. We summarized the current techniques by their cost and throughput. We also added some evaluation of the future development direction of the sequencing technique. Besides we also reconstructed the section of "Challenges of DNA-based data storage". The amended section is in [Page 14, Line 11- Page 17, Line 13].

6) Fig. 4, the appending figure has no label of Y axis.
Response: We thank you for your valuable suggestion. The corrected appending figure has been now re-uploaded accordingly.

Thank you again for the peer reviewing.

Best wishes,
Yue (Chantal) SHEN, Ph.D.
Genome Synthesis and Editing Platform, China National GeneBank
BGI-Research
Mobile: +86 150 1383 3483
Address: China National GeneBank (CNGB), Jinsha Road, Dapeng District, Shenzhen, Guangdong, China
Mail: shenyue@genomics.cn

Close
