# Supplementary material for: Carbon-based archiving: current progress and future prospects of DNA-based data storage
Source: Gigascience. 2019 Jun 20;8(6):giz075. doi: 10.1093/gigascience/giz075 (PMC6586197; doi:10.1093/gigascience/giz075)
Supplement: giz075_Reviewer_1_Report_Revision_1 — Jeff Nivala -- 1/22/2019 Reviewed [file giz075_reviewer_1_report_revision_1.pdf]

Reviewer Report

Title: Carbon-based archiving: the current progress and future prospects of DNA-based data storage

Version: Revision 1 **Date:** 1/22/2019

Reviewer name: Jeff Nivala

Reviewer Comments to Author:

This manuscript reviews the current state of digital data storage in DNA. Overall the manuscript covers the main aspects, key developments, and major works well. Appreciated are the differences in encoding schemes. Major and minor points that should be considered in the next draft of the manuscript are listed below. Also some of the text of the manuscript will require some editing for language and clarity.
-[Page 3, Lines 9-22]: A 4th unique feature of DNA that might be included is the ease and rapidity with which DNA can be replicated using, for example, PCR.
-[Page 4: Line 8]: The authors write "there is a trade-off between accuracy and redundancy". In my interpretation, this is counter-intuitive, as additional redundancy should reduce errors.
-[Page 4: Lines 13-15]: Concerning random access, many experimental works demonstrating DNA data storage do not have random access. Thus it may not necessarily be a requirement. Can the authors discuss this further?
-[Page 12: lines 5-6]: The amount of time is less informative than citing a number of bacterial divisions/replications over which the data is expected to mutate significantly.
-[Pages 11-12]: Concerning in-vivo storage, the authors fail to cite a number of early works in DNA data storage that included an in-vivo storage component. For instance: Bancroft 2001, Wong 2003, and Arita 2004.
-[Pages 11-12]: The authors might also want to mention other methods of storing data in vivo, for instance with recombinases, and other molecular recorders like Cas9.
-[Page 13, lines 23-25]: Is length really the major challenge? Why not just write-throughput in general, which can be increased by synthesis of longer strands (as stated), and/or by writing more strands in parallel (which is not mentioned) for instance by making larger, more dense oligo synthesis arrays.
-[Page 14, lines 16-20]: This paragraph is confusing, and should be re-written for clarity.
-[Table 1]: Costs for HiSeq2500 and NextSeq are missing "K" symbols.

Methods

Are the methods appropriate to the aims of the study, are they well described, and are necessary controls included? Choose an item.

Conclusions

Are the conclusions adequately supported by the data shown? Choose an item.

Reporting Standards

Does the manuscript adhere to the journal's guidelines on [minimum standards of reporting?]() Choose an item.

Choose an item.

Statistics

Are you able to assess all statistics in the manuscript, including the appropriateness of statistical tests used? Choose an item.

Quality of Written English

Please indicate the quality of language in the manuscript: Choose an item.

Declaration of Competing Interests

Please complete a declaration of competing interests, considering the following questions:

- Have you in the past five years received reimbursements, fees, funding, or salary from an organisation that may in any way gain or lose financially from the publication of this manuscript, either now or in the future?
- Do you hold any stocks or shares in an organisation that may in any way gain or lose financially from the publication of this manuscript, either now or in the future?
- Do you hold or are you currently applying for any patents relating to the content of the manuscript?
- Have you received reimbursements, fees, funding, or salary from an organization that holds or has applied for patents relating to the content of the manuscript?
- Do you have any other financial competing interests?
- Do you have any non-financial competing interests in relation to this paper?

If you can answer no to all of the above, write 'I declare that I have no competing interests' below. If your reply is yes to any, please give details below.

I'm a consultant to Oxford Nanopore Tech.

I agree to the open peer review policy of the journal. I understand that my name will be included on my report to the authors and, if the manuscript is accepted for publication, my named report including any attachments I upload will be posted on the website along with the authors' responses. I agree for my report to be made available under an Open Access Creative Commons CC-BY license (http://creativecommons.org/licenses/by/4.0/). I understand that any comments which I do not wish to be included in my named report can be included as confidential comments to the editors, which will not be published.

I agree to the open peer review policy of the journal

To further support our reviewers, we have joined with Publons, where you can gain additional credit to further highlight your hard work (see: https://publons.com/journal/530/gigascience). On publication of this paper, your review will be automatically added to Publons, you can then choose whether or not to claim your Publons credit. I understand this statement.

Yes Choose an item.
