# Supplementary material for: Carbon-based archiving: current progress and future prospects of DNA-based data storage
Source: Gigascience. 2019 Jun 20;8(6):giz075. doi: 10.1093/gigascience/giz075 (PMC6586197; doi:10.1093/gigascience/giz075)
Supplement: giz075_Reviewer_2_Report_Revision_1 — King L Chow, PhD -- 4/19/2019 Reviewed [file giz075_reviewer_2_report_revision_1.pdf]

Reviewer Report

Title: Carbon-based archiving: the current progress and future prospects of DNA-based data storage

Version: Revision 1 **Date:** 4/19/2019

Reviewer name: King L Chow, PhD

Reviewer Comments to Author:

The manuscript by Ping et al. aims to review the current status of DNA based data storage, archiving and retrieval. The review comes timely and covers some latest information available. The scope of coverage is appropriate. However, the content appears to be rather sketchy providing references, citing examples without properly describing the core principles and limitation of the methods. It seems to fit well for readers working closely in the community of DNA data storage areas, but for readers or GigaScience with a broader interest, the review may not provide sufficient depth for a good understanding of the progress of the field. Some suggestions include:

1. Page 3, Advantage of using DNA for storage would also include: easy amplification in vivo by live cells at very low cost, and possible amplification in vitro by enzymatic reaction, e.g., PCR or linear amplification in silico. Both approaches can be used to scale up the backup copy production. One should also consider the possible employment of repair system for correcting errors.
2. Page 5-10, the description of the coding schemes is quite sketchy. The outline for each approach was brief and was not well illustrated by the panels in the figure 1 and 2. Better schematics may help, without the need to go back to the original papers to make detailed comparison.
3. Page 10-12, in vivo and in vitro storage of the information - a thorough comparison of the pros and cons would be helpful, instead of factually describing what methodology is available. The error generated in vivo by mutation should be contrasted to the error in DNA synthesis technology to evaluate the limitation of these tools.
4. Page 13, line 4-10. A very typical way of this review in describing methodology citing the previous reports without describing the details and contrasting the differences sufficiently. It does not serve the purpose of a proper analysis of how each method advances the development of storage.
5. Page 14, sequencing accuracy issue was discussed concerning the data retrieval process. While table 1 summarizes the factual information of the technology available, no clear evaluation of the future direction is given, same as pointed out in (4) above.
6. Fig. 4, the appending figure has no label of Y axis.

Methods

Are the methods appropriate to the aims of the study, are they well described, and are necessary controls included? Choose an item.

Conclusions

Are the conclusions adequately supported by the data shown? Choose an item.

Reporting Standards

Does the manuscript adhere to the journal's guidelines on [minimum standards of reporting?] Choose an item.

Choose an item.

Statistics

Are you able to assess all statistics in the manuscript, including the appropriateness of statistical tests used? Choose an item.

Quality of Written English

Please indicate the quality of language in the manuscript: Choose an item.

Declaration of Competing Interests

Please complete a declaration of competing interests, considering the following questions:

- Have you in the past five years received reimbursements, fees, funding, or salary from an organisation that may in any way gain or lose financially from the publication of this manuscript, either now or in the future?
- Do you hold any stocks or shares in an organisation that may in any way gain or lose financially from the publication of this manuscript, either now or in the future?
- Do you hold or are you currently applying for any patents relating to the content of the manuscript?
- Have you received reimbursements, fees, funding, or salary from an organization that holds or has applied for patents relating to the content of the manuscript?
- Do you have any other financial competing interests?
- Do you have any non-financial competing interests in relation to this paper?

If you can answer no to all of the above, write 'I declare that I have no competing interests' below. If your reply is yes to any, please give details below.

I declare that I have no competing interests

I agree to the open peer review policy of the journal. I understand that my name will be included on my report to the authors and, if the manuscript is accepted for publication, my named report including any attachments I upload will be posted on the website along with the authors' responses. I agree for my report to be made available under an Open Access Creative Commons CC-BY license (http://creativecommons.org/licenses/by/4.0/). I understand that any comments which I do not wish to be included in my named report can be included as confidential comments to the editors, which will not be published.

I agree to the open peer review policy of the journal

To further support our reviewers, we have joined with Publons, where you can gain additional credit to further highlight your hard work (see: https://publons.com/journal/530/gigascience). On publication of this paper, your review will be automatically added to Publons, you can then choose whether or not to claim your Publons credit. I understand this statement.

Yes Choose an item.
